# New benzylresorcinol and benzylstilbene derivatives from the fruits of orchid *Galeola nudifolia* Lour. with anti-neuroinflammatory activity

**DOI:** 10.1039/d6ra06075g

**Published:** 2026-10-05

**Authors:** Duy-Hien Tran, Bien-Thuy Bui Nguyen, Yuh-Chiang Shen, Chia-Ching Liaw, Chih-Hua Chao, Hui-Chi Huang, I-Wen Lo, Chin-Yeu Chen, Mei-Hsien Lee, Yu-Chi Lin

**Affiliations:** a PhD Program in Clinical Drug Development of Herbal Medicine, College of Pharmacy, Taipei Medical University Taipei 11031 Taiwan lmh@tmu.edu.tw; b National Research Institute of Chinese Medicine, MOHW Taipei 112026 Taiwan yclin@nricm.edu.tw; c School of Pharmacy, University of Medicine and Pharmacy at Ho Chi Minh City Ho Chi Minh City 700000 Vietnam; d University of Health Sciences, Vietnam National University Ho Chi Minh City Ho Chi Minh City 700000 Vietnam; e Department of Pharmacy, School of Pharmaceutical Sciences, National Yang Ming Chiao Tung University Taipei 112304 Taiwan; f Graduate Institute of Natural Products, Kaohsiung Medical University Kaohsiung 807378 Taiwan; g School of Pharmacy, China Medical University Taichung 406040 Taiwan; h Chinese Medicine Research and Development Center, China Medical University Hospital Taichung 404327 Taiwan; i School of Chinese Medicine & Graduate Institute of Chinese Medicine, China Medical University Taichung 406040 Taiwan; j Department of Physical Therapy, Tzu-Hui Institute of Technology Pingtung 92641 Taiwan; k Graduate Institute of Pharmacognosy, College of Pharmacy, Taipei Medical University Taipei 11031 Taiwan

## Abstract

*Galeola nudifolia* is a medicinal orchid recognized as a valuable source of structurally diverse natural products with potential therapeutic applications. Its chemical composition and biological activities remain largely unexplored, particularly with respect to anti-neuroinflammatory activity. Bioassay-guided investigation of the non-polar fractions of *G. nudifolia* fruits led to the isolation of six new compounds, including three benzylresorcinol (1–3) and three benzylstilbene (4–6) derivatives, together with thirteen known compounds (7–19). Their structures were elucidated through comprehensive spectroscopic analyses, including HR-ESIMS, 1D and 2D NMR experiments. The anti-neuroinflammatory activities of all isolated compounds were evaluated in LPS-stimulated BV-2 microglial cells by measuring nitric oxide (NO) production. Compounds 1–6 and 8–13 exhibited significant inhibitory effects on NO production, with IC_50_ values ranging from 6.59 to 15.79 µM. Notably, compounds 6 (IC_50_ = 7.77 µM), 10 (IC_50_ = 7.48 µM), 11 (IC_50_ = 8.62 µM), and 12 (IC_50_ = 6.59 µM) showed superior inhibitory potencies compared to the positive control (grossamide, IC_50_ = 9.23 µM). Immunofluorescence analyses showed that compound 12 reduced iNOS expression and NF-κB p65 nuclear translocation, whereas molecular docking provided qualitative predictions of possible interactions of compounds 6 and 10–12 with iNOS and the IκBα/NF-κB complex. These findings expand the known chemical diversity of *G. nudifolia* and identify its benzylstilbene derivatives as natural products with anti-neuroinflammatory activity.

## Abstract 2

*Galeola nudifolia* fruits yielded new benzylresorcinol and benzylstilbene derivatives. Compound 12 reduced NO production, iNOS expression, and NF-κB p65 nuclear translocation in LPS-stimulated BV-2 cells.

## Graphical abstract

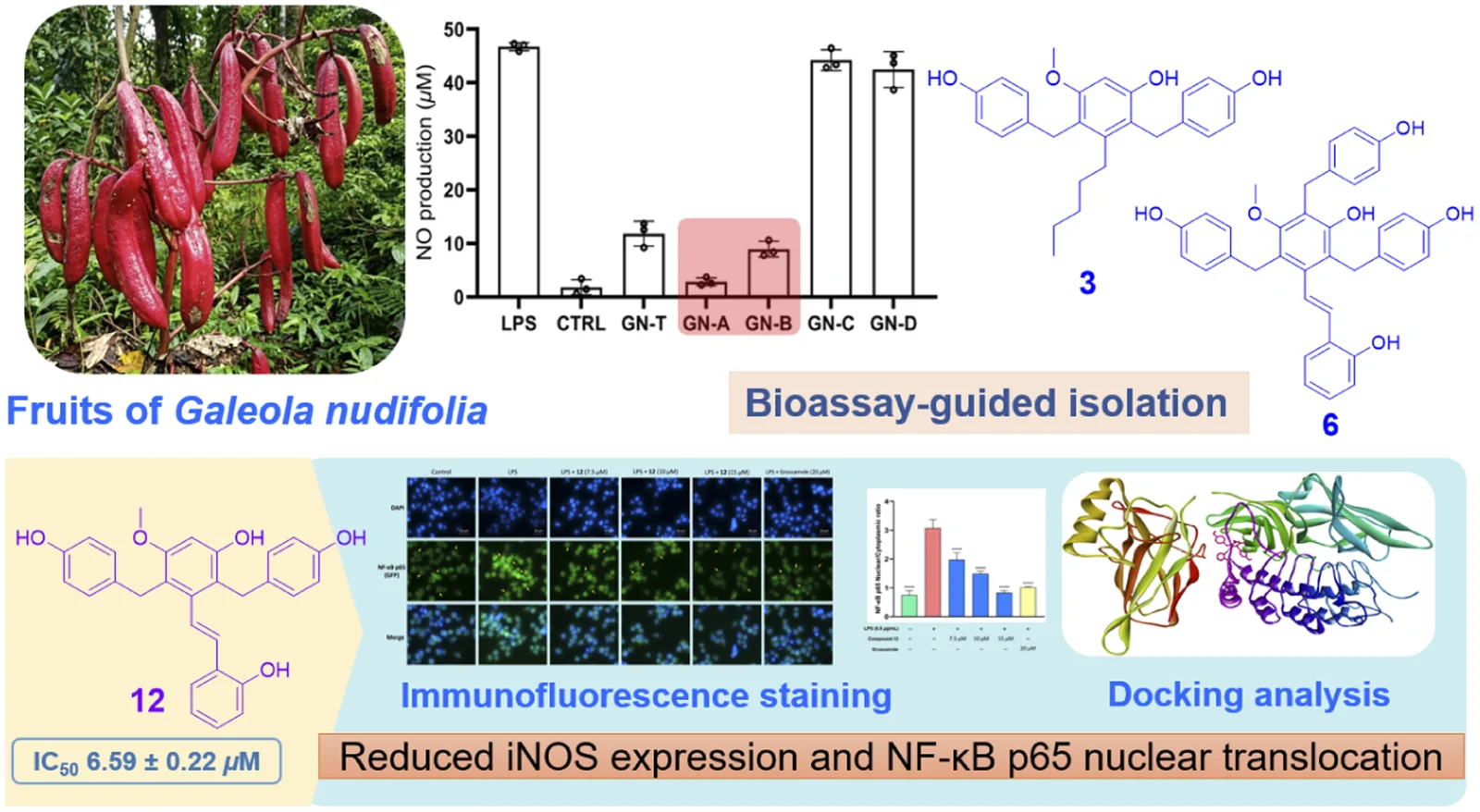

## Introduction

Neuroinflammation represents an intricate immunological cascade within the central nervous system (CNS) that critically drives the onset and advancement of various neurodegenerative pathologies, including Alzheimer's disease, Parkinson's disease, and other disorders associated with cognitive impairment. Chronic or deregulated neuroinflammatory responses precipitate progressive neuronal dysfunction and subsequent cell death.^1,2^ Microglia, the resident immune cells of the CNS, become activated in response to pathological stimuli such as lipopolysaccharide (LPS), resulting in excessive production of pro-inflammatory mediators, particularly nitric oxide (NO) and cytokines.^3^ Among the major inflammatory signaling pathways, NF-κB plays a central role in regulating microglial inflammatory responses. Upon activation, NF-κB translocates into the nucleus and induces the expression of inducible nitric oxide synthase (iNOS), leading to excessive NO production and subsequent oxidative and neuronal damage. Therefore, inhibition of the NF-κB/iNOS/NO axis represents an important strategy for suppressing neuroinflammation and protecting neuronal function.^4–6^ Plant-derived secondary metabolites have attracted considerable attention as potential anti-neuroinflammatory agents because of their ability to modulate NF-κB activation and suppress iNOS-mediated inflammatory responses.^7,8^

The Orchidaceae, one of the largest families of flowering plants with more than 25 000 species, is broadly distributed throughout tropical and subtropical regions worldwide. Phytochemical investigations have revealed that Orchidaceae species produce a wide range of secondary metabolites, including phenanthrenes, bibenzyls, flavonoids, lignans, and terpenoids.^9^ Furthermore, numerous orchid-derived compounds have demonstrated diverse biological activities, such as antioxidant, anti-inflammatory, antimicrobial, hepatoprotective, and anticancer effects, highlighting their potential for the development of health-promoting products and therapeutic agents.^10,11^

*Galeola nudifolia* Lour. (Orchidaceae) is a mycoheterotrophic orchid widely distributed across Southeast Asia, including Vietnam, Myanmar, Thailand, Laos, and the Philippines.^12^ Traditional medicinal practices among several ethnic minority groups in northern Vietnam have documented the use of medicinal liquor prepared from its fruits as a tonic for enhancing physical vitality and general well-being. A previous phytochemical study of *G. nudifolia* identified several phenolic constituents with acetylcholinesterase–inhibitory activity.^13^ In addition, species of the genus *Galeola* have been recognized as sources of structurally diverse bioactive metabolites.^14^ However, the anti-neuroinflammatory constituents of *G. nudifolia* have not been systematically investigated. Together with the traditional use of its fruits as a tonic, these findings provided the rationale for investigating the anti-neuroinflammatory constituents of *G. nudifolia* fruits.

In the present study, bioassay-guided isolation of the non-polar fractions of *G. nudifolia* fruits afforded six previously undescribed compounds, including three benzylresorcinol and three benzylstilbene derivatives, together with thirteen known compounds. Their anti-neuroinflammatory activities were evaluated by measuring NO production in LPS-stimulated BV-2 microglial cells. The most active compound was further evaluated by immunofluorescence analysis, while the four most active compounds were examined by molecular docking to explore possible interactions with iNOS and the IκBα/NF-κB complex.

## Experimental

### General experimental procedures

UV spectra were recorded on a UV-2450 spectrophotometer (Shimadzu, Kyoto, Japan). NMR spectra were acquired using an Avance III 600 MHz spectrometer (Bruker, Billerica, MA, USA), with residual solvent signals of CDCl_3_ (*δ*_H_ 7.26, *δ*_C_ 77.2) and CD_3_OD (*δ*_H_ 3.31, *δ*_C_ 49.0) used as internal standards. HR-ESIMS data were obtained on an LCMS-9030 Q-ToF mass spectrometer coupled with an LC-2050C 3D system (Shimadzu, Kyoto, Japan). Flash reversed-phase C18 chromatography and semipreparative HPLC separations were carried out on a Buchi C-850 Pure chromatography system (Büchi, Flawil, Switzerland) equipped with an EcoFlex C18 flash cartridge (80 g; Büchi) and a Cosmosil 5C_18_-AR-II column (5 µm, 20 mm i.d. × 250 mm; Nacalai Tesque, Kyoto, Japan), respectively. Open-column chromatography was performed using silica gel Chromatorex MB70-40/75 (60 µm; Fuji Silysia Chemical Ltd, Aichi, Japan) and Sephadex LH-20 (Cytiva, Marlborough, MA, USA). Absorbance measurements for biological assays were conducted using an Epoch 2 microplate spectrophotometer (BioTek Instruments, Agilent Technologies, Santa Clara, CA, USA). Primary antibodies (anti-NF-κB p65 and anti-iNOS) and secondary antibodies (Alexa Fluor^®^ 488 and 594) were purchased from Abcam (Cambridge, UK). Fluorescence intensity was acquired using a Cytation™ 5 cell imaging multi-mode reader (BioTek Instruments, Agilent Technologies, Santa Clara, CA, USA) with Gen 5 software (version 3.10).

### Plant material

Fruits of *Galeola nudifolia* Lour. were collected in Lai Chau province, Vietnam (September 2024). The plant material was authenticated by Dr Bien-Thuy Bui Nguyen, Faculty of Pharmacy, University of Health Sciences, Vietnam National University Ho Chi Minh City, Ho Chi Minh City, Vietnam. A voucher specimen (GNF-202409) was deposited in the Herbarium of the National Research Institute of Chinese Medicine, Taipei, Taiwan, for future reference.

### Extraction and bioassay – guided isolation

Dried fruits of *Galeola nudifolia* were powdered (1.0 kg) and extracted with 95% EtOH (5 L × 3) by ultrasonic-assisted extraction for 24 h per extraction cycle. The combined extracts were concentrated under reduced pressure to afford a crude ethanol extract (75 g). The crude extract was suspended in distilled water (1 L) and successively partitioned with *n*-hexane, EtOAc, and *n*-BuOH to yield the corresponding solvent fractions.

Evaluation of anti-neuroinflammatory activity revealed that the *n*-hexane (GN-A) and EtOAc (GN-B) fractions markedly inhibited NO production in LPS-stimulated BV-2 microglial cells, with inhibition rates of 93.9% and 80.9%, respectively, at a concentration of 50 µg mL^−1^ (Fig. S1). Based on their potent activities, these two fractions were selected for subsequent phytochemical investigation.

The *n*-hexane fraction (GN-A, 24.4 g) was subjected to silica gel flash column chromatography and eluted with a gradient of *n*-hexane–EtOAc (100 : 0 to 0 : 100, v/v) to afford 12 subfractions (A1–A12). These subfractions were subsequently evaluated for their anti-neuroinflammatory activity. Among them, subfractions A2, A4, A7, and A9 exhibited potent inhibitory effects on NO production, with inhibition rates of 91.0%, 96.7%, 97.1%, and 97.3%, respectively, at the tested concentration (Fig. S2).

Subfraction A2 (0.6 g) was purified by semipreparative RP-C18 HPLC using 40% CH_3_CN (v/v) as the mobile phase to yield compounds 1 (2.4 mg), 2 (2.2 mg), and 7 (1.5 mg). Subfraction A4 (2.3 g) was further fractionated on a Sephadex LH-20 column eluted with MeOH to produce four subfractions (A4.1–A4.4). Subsequent purification of subfraction A4.2 by semipreparative RP-C18 HPLC (60–65% MeOH, v/v) afforded compounds 4 (2.8 mg) and 18 (36.7 mg), whereas compound 3 (6.7 mg) was obtained from subfraction A4.3 by semipreparative RP-C18 HPLC using an isocratic system of 45% CH_3_CN (v/v). Subfraction A7 (1.5 g) was chromatographed over Sephadex LH-20 (MeOH), followed by semipreparative RP-C18 HPLC (40–45% CH_3_CN, v/v), to furnish compound 11 (29.8 mg). Subfraction A9 (3.4 g) was separated by flash RP-C18 column chromatography using a gradient of 30–60% MeOH (v/v), yielding three subfractions (A9.1–A9.3). Purification of subfraction A9.2 by semipreparative RP-C18 HPLC (40% CH_3_CN, v/v) afforded compounds 8 (3.2 mg) and 9 (3.6 mg). Further purification of subfraction A9.3 by semipreparative RP-C18 HPLC using 30–40% CH_3_CN (v/v) yielded compounds 6 (1.8 mg) and 19 (38.2 mg).

The EtOAc fraction (GN-B, 12.8 g) was subjected to silica gel flash column chromatography and eluted with an EtOAc–MeOH gradient (100 : 0 to 0 : 100, v/v) to afford nine subfractions (B1–B9). Bioactivity-guided screening revealed that subfractions B4, B5, and B8 exhibited strong inhibitory effects on NO production, with inhibition rates of 97.8%, 96.7%, and 91.2%, respectively (Fig. S3).

Subfraction B4 (0.8 g) was purified by semipreparative RP-C18 HPLC using a gradient of 25–40% MeOH (v/v), yielding compounds 5 (1.2 mg), 13 (11.5 mg), 14 (21.3 mg), and 15 (43.8 mg). Subfraction B5 (1.4 g) was further separated by semipreparative RP-C18 HPLC (20–30% CH_3_CN, v/v) to afford compound 12 (27.2 mg). Subfraction B8 (0.5 g) was purified by semipreparative RP-C18 HPLC with a gradient of 20–35% CH_3_CN (v/v), yielding compounds 10 (6.4 mg), 16 (4.6 mg), and 17 (1.7 mg) (Fig. 1).

**Fig. 1:**
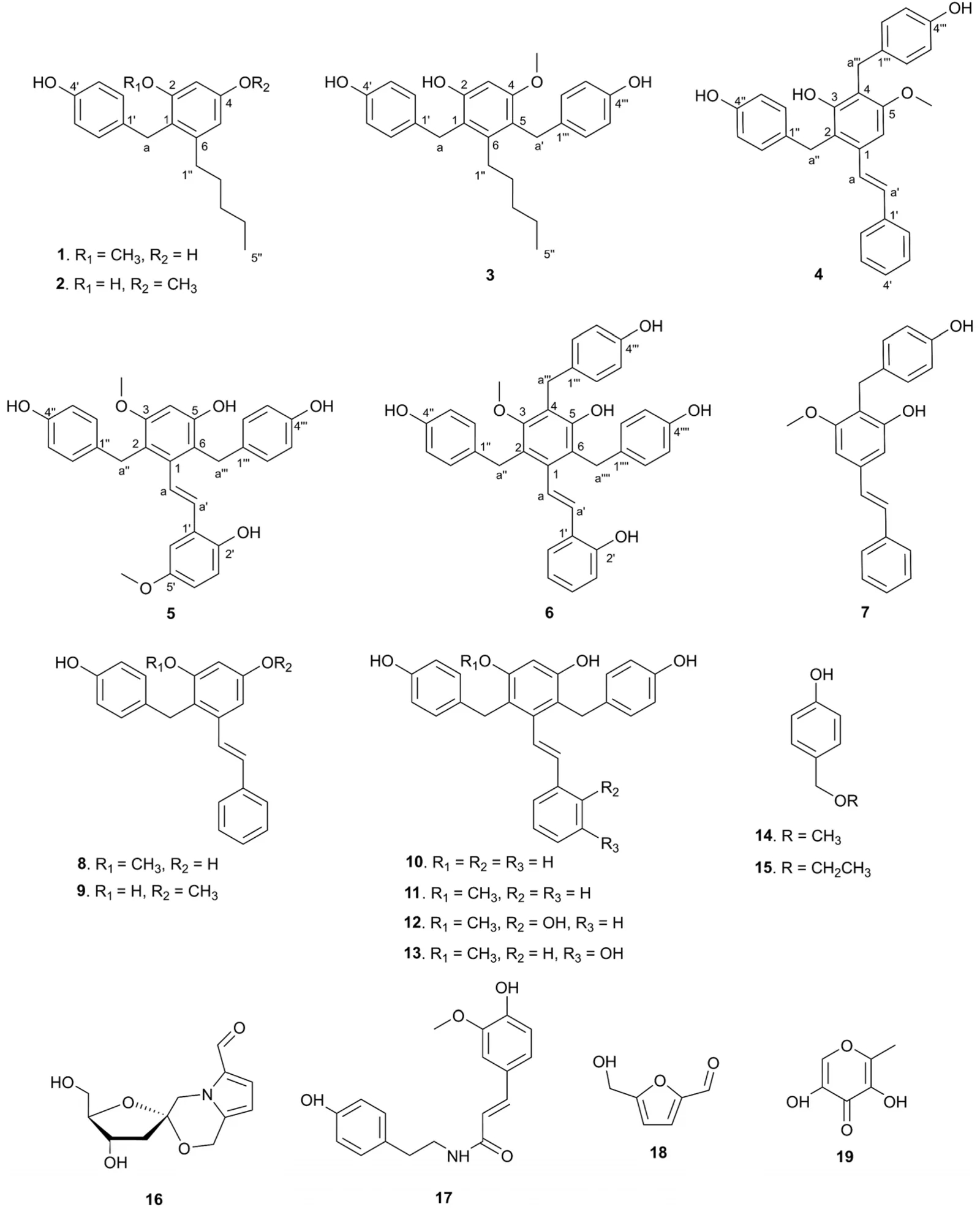
Chemical structures of compounds 1–19 isolated from *G. nudifolia*.

#### Galeonudin A (1)

Yellow amorphous powder, UV (MeOH) *λ*_max_ 198, 226, and 281 nm, ^1^H and ^13^C NMR data (600 MHz/150 MHz, CDCl_3_): see Table 1, HR-ESIMS *m*/*z* 301.1784 [M + H]^+^ (calcd for C_19_H_25_O_3_, *m*/*z* 301.1798).

**Table 1:** ^1^H (600 MHz) and ^13^C (150 MHz) NMR data of compounds 1–3

Position	1^a^		2^a^		3^b^	
	*δ*_C_, type	*δ*_H_ (*J* in Hz)	*δ*_C_, type	*δ*_H_ (*J* in Hz)	*δ*_C_, type	*δ*_H_ (*J* in Hz)
1	119.8, C	—	117.2, C	—	119.3, C	—
2	159.0, C	—	155.2, C	—	155.7, C	—
3	96.8, CH	6.31 d (2.4)	99.6, CH	6.29 d (2.4)	97.8, CH	6.43 s
4	154.8, C	—	158.9, C	—	158.2, C	—
5	108.1, CH	6.28 d (2.4)	107.8, CH	6.39 d (2.4)	120.0, C	—
6	144.0, C	—	144.1, C	—	143.8, C	—
2-OCH_3_	55.8, CH_3_	3.74 s	—	—	—	—
4-OCH_3_	—	—	55.3, CH_3_	3.77 s	55.9, CH_3_	3.73 s
a	29.9, CH_2_	3.89 s, 2H	30.3, CH_2_	3.91 s, 2H	31.2, CH_2_	3.88 s, 2H
a′	—	—	—	—	31.1, CH_2_	3.85 s, 2H
1′	134.1, C	—	132.4, C	—	134.4, C	—
2′/6′	129.2, CH	6.94 d (8.4)	129.3, CH	7.00 d (8.4)	130.0, CH	6.90 d (8.4)
3′/5′	115.1, CH	6.68 d (8.4)	115.6, CH	6.73 d (8.4)	115.8, CH	6.62 d (8.4)
4′	153.4, C	—	154.1, C	—	155.9, C	—
1″	33.2, CH_2_	2.47 m, 2H	33.9, CH_2_	2.54 m, 2H	31.0, CH_2_	2.41 m, 2H
2″	30.7, CH_2_	1.44 m, 2H	31.0, CH_2_	1.48 m, 2H	31.3, CH_2_	1.10 m, 2H
3″	32.0, CH_2_	1.26 m, 2H	32.0, CH_2_	1.29 m, 2H	33.6, CH_2_	1.18 m, 2H
4″	22.7, CH_2_	1.26 m, 2H	22.7, CH_2_	1.29 m, 2H	23.4, CH_2_	1.17 m, 2H
5″	14.1, CH_3_	0.85 t (7.2)	14.1, CH_3_	0.86 t (7.2)	14.4, CH_3_	0.79 t (7.2)
1‴					134.5, C	—
2‴/6‴					129.8, CH	6.82 d (8.4)
3‴/5‴					115.8, CH	6.59 d (8.4)
4‴					155.9, C	—

a Data were measured in CDCl_3_.

b Data were measured in CD_3_OD.

#### Galeonudin B (2)

Yellow amorphous powder, UV (MeOH) *λ*_max_ 197, 226, and 281 nm, ^1^H and ^13^C NMR data (600 MHz/150 MHz, CDCl_3_): see Table 1, HR-ESIMS *m*/*z* 301.1784 [M + H]^+^ (calcd for C_19_H_25_O_3_, *m*/*z* 301.1798).

#### Galeonudin C (3)

Yellow amorphous powder, UV (MeOH) *λ*_max_ 190, 226, and 283 nm, ^1^H and ^13^C NMR data (600 MHz/150 MHz, CD_3_OD): see Table 1, HR-ESIMS *m*/*z* 407.2197 [M + H]^+^ (calcd for C_26_H_31_O_4_, *m*/*z* 407.2217).

#### Galeonudin D (4)

Yellow amorphous powder, UV (MeOH) *λ*_max_ 194, 226, and 311 nm, ^1^H and ^13^C NMR data (600 MHz/150 MHz, CD_3_OD): see Table 2, HR-ESIMS *m*/*z* 439.1891 [M + H]^+^ (calcd for C_29_H_27_O_4_, *m*/*z* 439.1904).

**Table 2:** ^1^H (600 MHz) and ^13^C (150 MHz) NMR data of compounds 4–6

Position	4^a^		5^a^		6^a^	
	*δ*_C_, type	*δ*_H_ (*J* in Hz)	*δ*_C_, type	*δ*_H_ (*J* in Hz)	*δ*_C_, type	*δ*_H_ (*J* in Hz)
1	137.0, C	—	142.2, C	—	140.7, C	—
2	121.3, C	—	120.3, C	—	125.8, C	—
3	154.8, C	—	158.1, C	—	157.4, C	—
4	118.5, C	—	98.8, CH	6.51 s	122.0, C	—
5	158.1, C	—	155.5, C	—	153.8, C	—
6	101.5, CH	6.85 s	119.5, C	—	124.7, C	—
3-OCH_3_	—	—	55.9, CH_3_	3.77 s	62.2, CH_3_	3.49 s
5-OCH_3_	56.1, CH_3_	3.84 s	—	—	—	—
5′-OCH_3_	—	—	56.2, CH_3_	3.68 s	—	—
a	128.4, CH	7.33 d (16.2)	128.8, CH	6.87 d (16.8)	128.2, CH	6.83 d (16.8)
a′	130.5, CH	6.96 d (16.2)	130.4, CH	6.49 d (16.8)	130.5, CH	6.58 d (16.8)
a″	31.5, CH_2_	4.07 s, 2H	32.3, CH_2_	3.90 s, 2H	33.1, CH_2_	3.99 s, 2H
a‴	29.1, CH_2_	3.95 s, 2H	32.4, CH_2_	3.93 s, 2H	30.1, CH_2_	4.04 s, 2H
a′′′′	—	—	—	—	33.1, CH_2_	4.03 s, 2H
1′	139.2, C	—	126.3, C	—	125.9, C	—
2′	127.4, CH	7.40 d (7.8)	150.2, C	—	156.1, C	—
3′	129.6, CH	7.29 t (7.8)	117.4, CH	6.66 dd (9.0, 1.2)	116.5, CH	6.72 dd (7.8, 1.2)
4′	128.4, CH	7.19 m	115.5, CH	6.61 m	129.2, CH	7.00 td (7.8, 1.2)
5′	129.6, CH	7.29 t (7.8)	154.3, C	—	120.5, CH	6.70 m
6′	127.4, CH	7.40 d (7.8)	112.3, CH	6.62 m	128.0, CH	7.05 m
1″	133.7, C	—	134.6, C	—	134.2, C	—
2″/6″	130.2, CH	6.98 d (8.4)	130.2, CH	6.87 d (8.4)	130.3, CH	6.86 d (8.4)
3″/5″	116.0, CH	6.65 d (8.4)	115.7, CH	6.59 d (8.4)	115.8, CH	6.61 d (8.4)
4″	156.2, C	—	155.8, C	—	156.0, C	—
1‴	133.6, C	—	134.5, C	—	133.4, C	—
2‴/6‴	130.3, CH	7.03 d (8.4)	130.4, CH	6.94 d (8.4)	130.2, CH	7.04 d (8.4)
3‴/5‴	115.8, CH	6.63 d (8.4)	115.7, CH	6.61 d (8.4)	115.9, CH	6.67 d (8.4)
4‴	156.1, C	—	155.8, C	—	156.3, C	—
1′′′′					133.6, C	—
2′′′′/6′′′′					130.3, CH	6.91 d (8.4)
3′′′′/5′′′′					115.9, CH	6.63 d (8.4)
4′′′′					156.0, C	—

a Data were measured in CD_3_OD.

#### Galeonudin E (5)

Yellow amorphous powder, UV (MeOH) *λ*_max_ 194, 225, 275, and 322 nm, ^1^H and ^13^C NMR data (600 MHz/150 MHz, CD_3_OD): see Table 2, HR-ESIMS *m*/*z* 485.1942 [M + H]^+^ (calcd for C_30_H_29_O_6_, *m*/*z* 485.1959).

#### Galeonudin F (6)

Yellow amorphous powder, UV (MeOH) *λ*_max_ 194, 225, 277, and 307 nm, ^1^H and ^13^C NMR data (600 MHz/150 MHz, CD_3_OD): see Table 2, HR-ESIMS *m*/*z* 561.2247 [M + H]^+^ (calcd for C_36_H_33_O_6_, *m*/*z* 561.2272).

### Cell culture and anti-neuroinflammatory activity

The BV-2 murine microglial cell line was kindly provided by Dr Greer M. Murphy, Jr from the Department of Psychiatry, Stanford University School of Medicine, Stanford, CA, USA. The cells were cultured in Dulbecco's modified Eagle medium (DMEM; Gibco, USA) supplemented with 5% fetal bovine serum (FBS) and 1% penicillin–streptomycin. Cells were maintained at 37 °C in a humidified atmosphere containing 5% CO_2_. For the nitric oxide (NO) assay, BV-2 cells were seeded in 96-well plates at a density of 5 × 10^4^ cells per well and allowed to adhere for 4 h. The cells were then pretreated with test samples (50 µg mL^−1^ for extracts or fractions and 2.5–20 µM for isolated compounds) for 1 h, followed by stimulation with lipopolysaccharide (LPS, 0.5 µg mL^−1^) to induce an inflammatory response. After 24 h of incubation, NO production in the culture supernatant was determined using the Griess reaction, which consists of an equal volume mixture of 0.1% *N*-(1-naphthyl) ethylenediamine dihydrochloride (NED) and 1% sulfanilamide prepared in 6% phosphoric acid. Absorbance was measured at 550 nm, and nitrite concentrations were calculated using a sodium nitrite standard curve.^15^ Grossamide (TargetMol Chemicals Inc., USA) was used as a positive control.

### Cell viability assay

BV-2 cells were seeded in 96-well plates at a density of 3 × 10^4^ cells per well and allowed to adhere for 4 h. The cells were then co-treated with test samples and LPS (0.5 µg mL^−1^) for 24 h, using 50 µg mL^−1^ for extracts or fractions and 20 µM for isolated compounds as the highest tested concentrations. Cell viability was assessed using the MTS (3-(4,5-dimethylthiazol-2-yl)-5-(3-carboxymethoxyphenyl)-2-(4-sulfophenyl)-2*H*-tetrazolium; Abcam, UK) assay.^16^ Following treatment, 20 µL of MTS solution (2 mg mL^−1^) containing phenazine ethosulfate (PES, 0.2 mg mL^−1^) was added to each well, and the plates were incubated for an additional 4 h at 37 °C. Absorbance of the resulting formazan product was measured at 490 nm.

### Immunofluorescence staining

Immunofluorescence staining was performed to investigate the anti-neuroinflammatory mechanism of compound 12 through its effects on NF-κB p65 nuclear translocation and iNOS expression in BV-2 microglial cells.^17^ BV-2 cells were seeded into 24-well plates at a density of 2 × 10^5^ cells per well and allowed to adhere for 4 h. The cells were then pretreated with various concentrations of compound 12 for 1 h prior to stimulation with LPS (0.5 µg mL^−1^). Following LPS exposure for 1 h (for NF-κB p65 assay) or 24 h (for iNOS assay), the cells were fixed with 4% paraformaldehyde, permeabilized using 0.1% Triton X-100, and blocked with 3% bovine serum albumin (BSA). The cells were incubated overnight at 4 °C with primary antibodies against NF-κB p65 (Abcam, ab16502; 1 : 1000 dilution) or iNOS (Abcam, ab49999; 1 : 2000 dilution). After washing, the corresponding Alexa Fluor 488- or Alexa Fluor 594-conjugated secondary antibodies were applied for 1 h at room temperature. Cell nuclei were counterstained with DAPI. Fluorescence images were acquired using excitation/emission wavelengths of 469/525 nm for NF-κB p65 and 586/647 nm for iNOS. All experiments were performed in triplicate. Quantitative analyses were conducted using eight randomly selected microscopic fields from control, LPS-treated, and sample-treated groups. Fluorescence intensity and the nuclear-to-cytoplasmic translocation ratio were determined and used for statistical evaluation.

### Molecular docking analysis

The crystal structures of human inducible nitric oxide synthase (iNOS; PDB ID: 1NSI) and the IκBα/NF-κB complex (PDB ID: 1IKN) were retrieved from the RCSB protein data bank (https://www.rcsb.org). Water molecules and co-crystallized ligands were removed using BIOVIA Discovery Studio Visualizer (version 4.5). The 3D structures of the ligands were generated using ChemDraw 3D (version 22.0) and subsequently energy-minimized using the MMFF94 force field.

The molecular docking simulation was performed using AutoDock tools (version 1.5.7).^18^ Briefly, polar hydrogen atoms were added, Gasteiger charges were assigned, and all rotatable bonds were defined. The prepared protein and ligand structures were converted into PDBQT format for docking. Grid box parameters for iNOS^19^ and IκBα/NF-κB^20^ were set according to previously reported studies, including grid spacing and center coordinates as described in the literature. Docking calculations were carried out using the Lamarckian genetic algorithm with 100 independent runs. The resulting conformations were ranked based on predicted binding free energies, and the lowest-energy pose with the most favorable binding interactions was selected for further analysis. Protein–ligand interactions were visualized and analyzed using BIOVIA Discovery Studio Visualizer. 1400 W (*N*-3-aminomethyl benzyl acetamidine) and BAY 11-7082 ((*E*)3-(4-methylphenyl) sulfonyl-2-propenenitrile) were used as reference ligands for docking with iNOS and IκBα/NF-κB, respectively.

### Statistical analysis

Statistical assessment of the *in vitro* data was performed using GraphPad Prism (version 10.2.3). Results were expressed as the mean ± standard deviation (SD) of three independent replicates. Differences among multiple groups were assessed by one-way analysis of variance (ANOVA), followed by Dunnett's *post hoc* multiple comparison test. Dose–response relationships were evaluated using linear or nonlinear regression analysis of inhibition rates *versus* sample concentrations. A *p* value < 0.05 was considered statistically significant.

## Results and discussion

### Structure elucidation of isolated compounds

Compound 1 was obtained as a yellow amorphous powder. The HR-ESIMS spectrum of 1 demonstrated a positive ion peak at *m*/*z* 301.1784 [M + H]^+^ (calcd for C_19_H_25_O_3_, *m*/*z* 301.1798), corresponding to the molecular formula C_19_H_24_O_3_ with 8 degrees of unsaturation. The ^1^H NMR data displayed two *meta*-coupled aromatic protons at *δ*_H_ 6.31 (d, *J* = 2.4 Hz, H-3) and 6.28 (d, *J* = 2.4 Hz, H-5), together with a *para*-substituted A_2_B_2_ spin system at *δ*_H_ 6.94 (d, *J* = 8.4 Hz, H-2′/H-6′) and 6.68 (d, *J* = 8.4 Hz, H-3′/H-5′), a methoxy group (*δ*_H_ 3.74 s), and a methyl proton signal (*δ*_H_ 0.85 t, *J* = 7.2 Hz). In the ^13^C NMR spectrum, the 10 resolved aromatic signals accounted for 12 carbons due to the double intensity of the resonances at *δ*_C_ 129.2 and 115.1, which indicated the co-existence of a symmetric *para*-disubstituted benzene ring and another tetrasubstituted benzene moiety. The remaining six distinct aromatic signals were classified into four quaternary carbons (*δ*_C_ 159.0, 154.8, 144.0, and 119.8) and two methines (*δ*_C_ 108.1 and 96.8). In the aliphatic region, one methoxy at *δ*_C_ 55.8, as well as six aliphatic carbon signals at *δ*_C_ 33.2, 32.0, 30.7, 29.9, 22.7, and 14.1 were observed. The *p*-hydroxyphenyl ring was defined by the HMBC correlations from *δ*_H_ 6.94 (H-2′/6′) to the phenolic quaternary carbon C-4′ (*δ*_C_ 153.4) and from *δ*_H_ 6.68 (H-3′/5′) to C-1′ (*δ*_C_ 134.1). Concurrently, the resorcinol ring was established by the HMBC correlations from the aromatic protons at *δ*_H_ 6.31 (H-3) and 6.28 (H-5), which both showed long-range correlations to C-1 (*δ*_C_ 119.8) and C-4 (*δ*_C_ 154.8), together with the correlation from H-5 to C-3 (*δ*_C_ 96.8). Subsequently, the methoxy group was unambiguously assigned to C-2 based on the HMBC cross-peak from the methoxy protons at *δ*_H_ 3.74 to C-2 (*δ*_C_ 159.0). Furthermore, the central bridging methylene protons at *δ*_H_ 3.89 (H-*a*) exhibited key cross-ring HMBC correlations to C-1, C-2, and C-6 (*δ*_C_ 144.0) of the resorcinol core, and simultaneously to C-1′ and C-2′/6′ (*δ*_C_ 129.2) of the *p*-hydroxyphenyl unit, demonstrating that this methylene group directly connects the two discrete benzene ring systems *via* C-1 and C-1′, respectively. Notably, key ^1^H–^1^H COSY correlations led to the establishment of a continuous proton spin system consisting of H-1″ (*δ*_H_ 2.47)/H-2″ (*δ*_H_ 1.44)/H-3″(*δ*_H_ 1.26)/H-4″ (*δ*_H_ 1.26)/H-5″ (*δ*_H_ 0.85). The definitive attachment of this linear pentyl chain at C-6 of the resorcinol ring was established by key HMBC correlations from the methylene protons H-2″ to carbon signals at *δ*_C_ 119.8 (C-1), 108.1 (C-5), and 144.0 (C-6). Based on the detailed spectroscopic analyses, the chemical structure of compound 1 was elucidated and named galeonudin A.

Compound 2 was obtained as a yellow amorphous powder. Its molecular formula was determined to be C_19_H_24_O_3_ based on the positive HR-ESIMS spectrum exhibited a protonated molecular ion peak at *m*/*z* 301.1784 [M + H]^+^ (calcd for C_19_H_25_O_3_, *m*/*z* 301.1798), which was identical to that of 1 and implied a structural isomerism between these two compounds. The ^1^H and ^13^C NMR spectroscopic data of 2 (Table 1) closely resembled those of 1, displaying an identical symmetric *p*-hydroxyphenyl ring, a central bridging methylene group (*δ*_H_ 3.91, H-*a*), and a linear pentyl side chain, thereby suggesting they share the same core carbon framework. Detailed analysis of the HMBC spectrum revealed a key correlation from the methoxy protons at *δ*_H_ 3.77 to the downfield oxygenated quaternary carbon at *δ*_C_ 158.9 (C-4), establishing the methoxy group at C-4 rather than C-2 as in 1. Therefore, compound 2 was elucidated as a new benzylresorcinol derivative, representing a positional isomer of 1, and named galeonudin B.

Compound 3 was obtained as a yellow amorphous powder. The molecular formula of C_26_H_30_O_4_ was determined from the HR-ESIMS protonated ion peak at *m*/*z* 407.2197 [M + H]^+^ (calcd for C_26_H_31_O_4_, *m*/*z* 407.2217), indicating 12 degrees of hydrogen deficiency. The ^1^H NMR spectrum displayed signals attributable to two *para*-substituted A_2_B_2_ aromatic systems at *δ*_H_ 6.90 (d, *J* = 8.4 Hz, H-2′/6′), 6.82 (d, *J* = 8.4 Hz, H-2‴/6‴), 6.62 (d, *J* = 8.4 Hz, H-3′/5′), and 6.59 (d, *J* = 8.4 Hz, H-3‴/5‴), one methoxy group at *δ*_H_ 3.73, and a pentyl side-chain signals comparable to those observed for 1 and 2. The NMR spectroscopic data of 3 closely resembled those of 2, indicating that both compounds shared the same benzylresorcinol framework bearing a methoxy group and a pentyl side chain. The HMBC correlations from H-*a* (*δ*_H_ 3.88) to C-1′, C-2′/C-6′, C-1, C-2, and C-6, together with the correlation from 4-OMe (*δ*_H_ 3.73) to C-4 (*δ*_C_ 158.2), supported the same *p*-hydroxybenzyl and methoxy substitution pattern as in 2. The linear pentyl side chain was confirmed by the COSY sequence H-1″ (*δ*_H_ 2.41)/H-2″ (*δ*_H_ 1.10)/H-3″ (*δ*_H_ 1.18)/H-4″ (*δ*_H_ 1.17)/H-5″ (*δ*_H_ 0.79) and by HMBC correlations from H-1″ to C-5 (*δ*_C_ 120.0) and C-6 (*δ*_C_ 143.8). The major difference between 3 and 2 was the presence of an additional *p*-hydroxybenzyl unit, as indicated by an extra A_2_B_2_ aromatic spin system and an additional benzylic methylene signal H-*a*′ (*δ*_H_ 3.85). Key HMBC correlations from H-*a*′ to C-1‴ (*δ*_C_ 134.5), C-2‴/C-6‴ (*δ*_C_ 129.8) and to C-4 (*δ*_C_ 158.2)/C-5 (*δ*_C_ 120.0)/C-6 (*δ*_C_ 143.8) placed this additional *p*-hydroxybenzyl substituent at C-5 of the resorcinol ring. Thus, compound 3 was elucidated as a new dibenzyl-substituted benzylresorcinol derivative and named galeonudin C (Fig. 2).

**Fig. 2:**
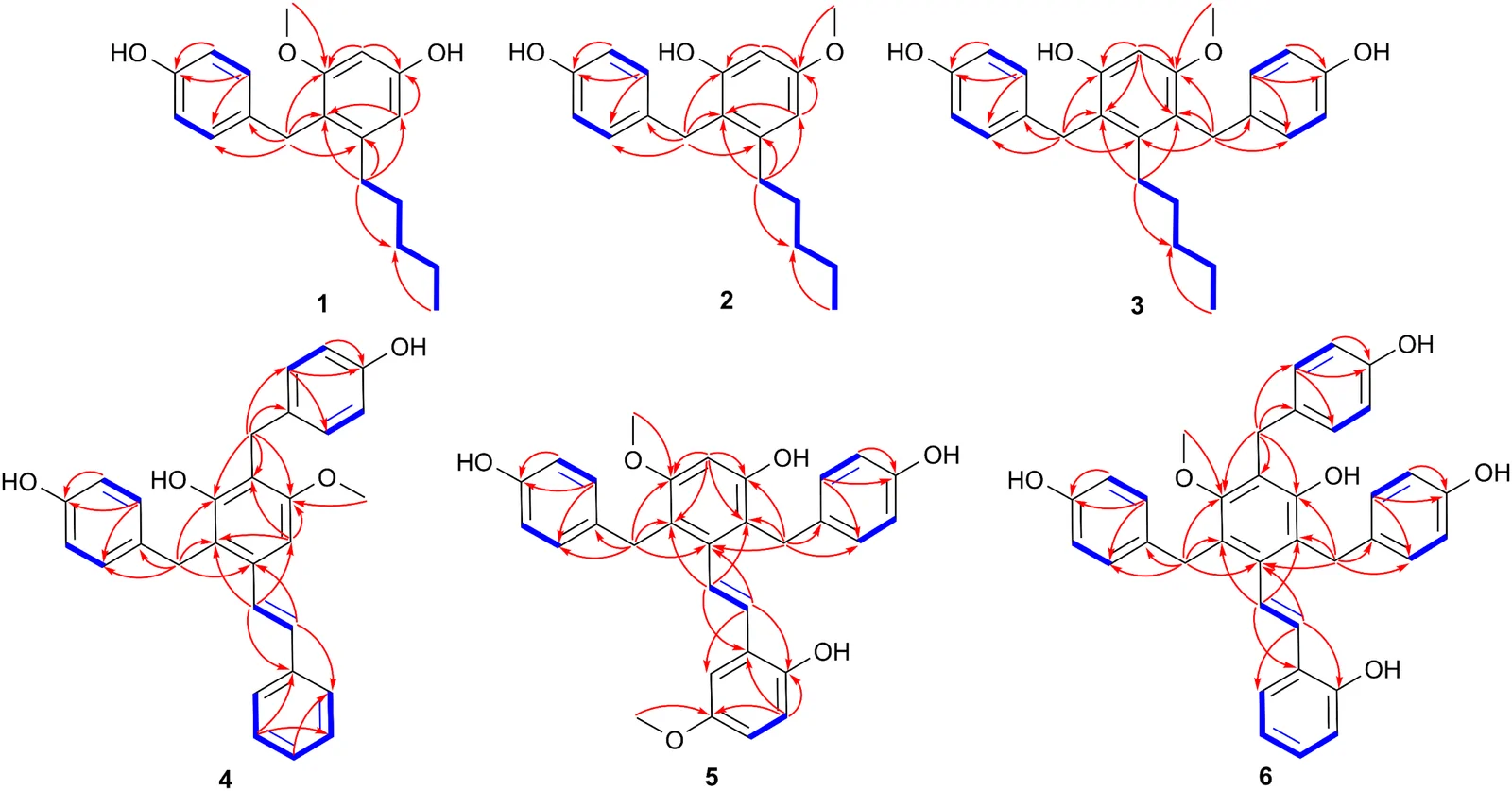
Key HMBC (red arrows) and COSY (blue bold bonds) correlations of compounds 1–6.

Compound 4 was obtained as a yellow amorphous powder. The HR-ESIMS showed an ion peak at *m*/*z* 439.1891 [M + H]^+^, consistent with the molecular formula C_29_H_26_O_4_ (calcd for C_29_H_27_O_4_, *m*/*z* 439.1904), indicating 17 degrees of unsaturation. The ^1^H NMR spectrum displayed signals characteristic of an *E*-configured olefinic bond at *δ*_H_ 7.33 (d, *J* = 16.2 Hz, H-*a*) and 6.96 (d, *J* = 16.2 Hz, H-*a*′), two A_2_B_2_ aromatic spin systems at *δ*_H_ 7.03 (d, *J* = 8.4 Hz, H-2‴/6‴), 6.98 (d, *J* = 8.4 Hz, H-2″/6″), 6.65 (d, *J* = 8.4 Hz, H-3″/5″), and 6.63 (d, *J* = 8.4 Hz, H-3‴/5‴), together with one methoxy group at *δ*_H_ 3.84. The ^13^C spectrum (Table 2) showed 26 sp^2^ carbons, together with two methylene carbons at *δ*_C_ 31.5 and 29.1, and one methoxy carbon at *δ*_C_ 56.1. The central resorcinol-type aromatic core was supported by HMBC correlations from H-6 (*δ*_H_ 6.85) to C-2 (*δ*_C_ 121.3), C-4 (*δ*_C_ 118.5), and C-5 (*δ*_C_ 158.1). The HMBC correlations from H-*a* to C-2, C-6 (*δ*_C_ 101.5), and C-1′ (*δ*_C_ 139.2), and from H-*a*′ to C-1 (*δ*_C_ 137.0) and C-2′/C-6′ (*δ*_C_ 127.4), together with the COSY fragment H-2′ (*δ*_H_ 7.40)/H-3′ (*δ*_H_ 7.29)/H-4′ (*δ*_H_ 7.19)/H-5′/H-6′, established the *E*-stilbene framework of 4. The remaining substituents at C-2 and C-4 were assigned as *p*-hydroxybenzyl groups based on the HMBC correlations of the corresponding methylene protons. The HMBC correlations from H-*a*″ (*δ*_H_ 4.07) to the central aromatic carbons C-1, C-2, and C-3 (*δ*_C_ 154.8), together with correlations to C-1″ (*δ*_C_ 133.7) and C-2″/C-6″ (*δ*_C_ 130.2), established the attachment of one *p*-hydroxybenzyl group at C-2. Likewise, H-*a*‴ (*δ*_H_ 3.95) showed HMBC correlations to C-3, C-4, and C-5, as well as to C-1‴ (*δ*_C_ 133.6) and C-2‴/C-6‴ (*δ*_C_ 130.3), placing the second *p*-hydroxybenzyl substituent at C-4. The methoxy group was assigned to C-5 by the HMBC correlation from 5-OCH_3_ (*δ*_H_ 3.84) to C-5. Accordingly, compound 4 was characterized as a new benzylstilbene derivative and named galeonudin D.

Compound 5 was obtained as a yellow amorphous powder. Its molecular formula, C_30_H_28_O_6_, was deduced from its HR-ESIMS data (*m*/*z* 485.1942 [M + H]^+^, calcd for C_30_H_29_O_6_, *m*/*z* 485.1959), demonstrating 17 degrees of unsaturation. ^1^H NMR spectrum displayed two olefinic protons at *δ*_H_ 6.87 (H-*a*) and 6.49 (H-*a*′) with *J* values of 16.8 Hz characterized for an *E*-configured double bond, four *ortho*-coupled doublets at *δ*_H_ 6.94 (d, *J* = 8.4 Hz, H-2‴/6‴), 6.87 (d, *J* = 8.4 Hz, H-2″/6″), 6.61 (d, *J* = 8.4 Hz, H-3‴/5‴), and 6.59 (d, *J* = 8.4 Hz, H-3″/5″), indicating two A_2_B_2_ spin systems attributable to two *para*-disubstituted phenyl rings, together with two methoxy singlets resonated at *δ*_H_ 3.77 and 3.68. The ^13^C spectrum showed 26 sp^2^ carbons, together with two methylene carbons at *δ*_C_ 32.4 and 32.3, along with two *O*-methyl groups at *δ*_C_ 56.2 and 55.9. Overall, the NMR data suggested that compound 5 shared a benzylstilbene skeleton closely related to that of 4. As in compound 4, the HMBC correlations from H-*a* and H-*a*′ supported the presence of an *E*-stilbene partial structure in 5. However, unlike the monosubstituted phenyl ring in 4, the C-1′–C-6′ aromatic ring of 5 was assigned as a 2′-hydroxy-5′-methoxyphenyl moiety based on HMBC correlations from H-*a*′ (*δ*_H_ 6.49) to C-2′ (*δ*_C_ 150.2) and C-6′ (*δ*_C_ 112.3) and from H-3′ (*δ*_H_ 6.66) to C-1′ (*δ*_C_ 126.3) and C-5′ (*δ*_C_ 154.3), together with the COSY fragment H-3′/H-4′ and the HMBC correlation from 5′-OCH_3_ (*δ*_H_ 3.68) to C-5′ (*δ*_C_ 154.3). The chemical shifts were in agreement with those reported for related 2′-hydroxy-5′-methoxyphenyl derivatives.^21^ In addition, the remaining substituents at C-2 and C-6 were assigned as *p*-hydroxybenzyl groups. This assignment was supported by HMBC correlations from H-*a*″ (*δ*_H_ 3.90) to C-1 (*δ*_C_ 142.2), C-2 (*δ*_C_ 120.3), and C-3 (*δ*_C_ 158.1), as well as to C-1″ and C-2″/C-6″, establishing one *p*-hydroxybenzyl group at C-2. Similarly, HMBC correlations from H-*a*‴ (*δ*_H_ 3.93) to C-1 (*δ*_C_ 142.2), C-5 (*δ*_C_ 155.5), and C-6 (*δ*_C_ 119.5), together with correlations to C-1‴ (*δ*_C_ 134.5) and C-2‴/C-6‴ (*δ*_C_ 130.4), placed the second *p*-hydroxybenzyl group at C-6. The remaining methoxy group was assigned to C-3 based on the HMBC correlation from 3-OCH_3_ (*δ*_H_ 3.77) to C-3. Consequently, compound 5 was characterized and given the name galeonudin E.

Compound 6 was obtained as a yellow amorphous powder. The molecular formula of 6 was assigned as C_36_H_32_O_6_ based on positive-ion HR-ESIMS at *m*/*z* 561.2247 [M + H]^+^ (calcd for C_36_H_33_O_6_, *m*/*z* 561.2272), corresponding to 21 degrees of unsaturation. The ^1^H NMR spectrum of 6 exhibited resonances attributable to an *E*-configured olefinic unit (*δ*_H_ 6.83, H-*a* and 6.58, H-*a*′), three *para*-substituted phenyl rings (*δ*_H_ 7.04, 6.91, 6.86, 6.67, 6.63, and 6.61), and one methoxy singlet (*δ*_H_ 3.49). The ^13^C NMR data of 6 revealed six oxygen-bearing aromatic carbons (*δ*_C_ 157.4, 156.3, 156.1, 156.0, 156.0, and 153.8), three aromatic methylene carbons (*δ*_C_ 33.1, 33.1, and 30.1), and one methoxy (*δ*_C_ 62.2). The structure of 6 was first established from the COSY fragment H-3′ (*δ*_H_ 6.72)/H-4′ (*δ*_H_ 7.00)/H-5′ (*δ*_H_ 6.70)/H-6′ (*δ*_H_ 7.05), which was connected by HMBC correlations from H-*a*′ (*δ*_H_ 6.58) to the oxygen-bearing aromatic carbon C-2′ (*δ*_C_ 156.1) and C-6′ (*δ*_C_ 128.0), from H-4′ (*δ*_H_ 7.00) and H-6′ (*δ*_H_ 7.05) to C-2′, and from H-5′ (*δ*_H_ 6.70) to C-1′ (*δ*_C_ 125.9), to establish the *E*-2′-hydroxystyryl unit of 6. The three *p*-hydroxybenzyl groups were identified by HMBC correlations from their methylene protons to the corresponding *p*-hydroxyphenyl rings. Thus, H-*a*″ (*δ*_H_ 3.99) showed correlations to C-1″ (*δ*_C_ 134.2) and C-2″/C-6″ (*δ*_C_ 130.3), H-*a*‴ (*δ*_H_ 4.04) to C-1‴ (*δ*_C_ 133.4) and C-2‴/C-6‴ (*δ*_C_ 130.2), along with H-*a*′′′′ (*δ*_H_ 4.03) to C-1′′′′ (*δ*_C_ 133.6) and C-2′′′′/C-6′′′′ (*δ*_C_ 130.3), confirming the presence of three *p*-hydroxybenzyl units. Moreover, HMBC correlations from H-*a* (*δ*_H_ 6.83) to C-1 (*δ*_C_ 140.7), C-2 (*δ*_C_ 125.8), C-6 (*δ*_C_ 124.7), and C-1′ (*δ*_C_ 125.9) established the attachment of the *E*-2′-hydroxystyryl unit at C-1 of the central aromatic core. In addition, HMBC correlations from H-*a*″ (*δ*_H_ 3.99) to C-1, C-2, and C-3 (*δ*_C_ 157.4), from H-*a*‴ (*δ*_H_ 4.04) to C-3, C-4 (*δ*_C_ 122.0), and C-5 (*δ*_C_ 153.8), and from H-*a*′′′′ (*δ*_H_ 4.03) to C-4, C-5, and C-6 (*δ*_C_ 124.7) established the attachment of the three *p*-hydroxybenzyl groups at C-2, C-4, and C-6, respectively. The methoxy group was assigned to C-3 by the HMBC correlation from 3-OCH_3_ (*δ*_H_ 3.49) to C-3, while the remaining hydroxy group was placed at C-5 to satisfy the molecular formula and NMR data. Based on the combined spectroscopic evidence, compound 6 was characterized as a previously undescribed benzylstilbene derivative for which the name galeonudin F was proposed.

Thirteen known compounds were identified as (*E*)-5′-hydroxy-4′-(4-hydroxybenzyl)-3′-methoxystilbene (7),^22^ (*E*)-5′-hydroxy-2′-(4-hydroxybenzyl)-3′-methoxystilbene (8), (*E*)-3′-hydroxy-2′-(4-hydroxybenzyl)-5′-methoxystilbene (9),^21^ (*E*)-2,4-bis(4-hydroxybenzyl)-3,5-dihydroxystilbene (10),^23^ (*E*)-3′-hydroxy-2′,4′-bis(4-hydroxybenzyl)-5′-methoxystilbene (11), (*E*)-2,3′-dihydroxy-2′,6′-bis(4-hydroxybenzyl)-5′-methoxystilbene (12),^21^ blestanol M (13),^24^*p*-hydroxybenzyl methyl ether (14), *p*-hydroxybenzyl ethyl ether (15),^25^ pollenopyrroside A (16),^26^ (*E*)-*N*-feruloyltyramine (17),^27^ 5-hydroxymethyl-2-furfural (18),^28^ and 5-hydroxymaltol (19)^29^ by comparison of their HR-MS and NMR data with those reported in the literature. To the best of our knowledge, these compounds were firstly reported from *G. nudifolia*. To evaluate whether the methoxylated compounds were artifacts formed during purification, the HPLC-PDA profiles of the *n*-hexane and EtOAc layers were compared with those of the purified new compounds 1–6. Peaks corresponding to compounds 1–6 were already detectable in the respective layers before subsequent chromatographic purification (Fig. S48). Notably, methoxylated compounds 1 and 2 were purified using a CH_3_CN-containing mobile phase without MeOH. Moreover, despite the substantially longer exposure to EtOH during extraction, no corresponding ethoxy analogues were detected. Together, these observations support the natural occurrence of the isolated methoxylated compounds rather than their formation as solvent-induced artifacts.

### *In vitro* anti-neuroinflammatory activity

All isolated compounds were evaluated for their anti-neuroinflammatory activity by measuring the inhibition of nitric oxide (NO) production in LPS-stimulated BV-2 microglial cells. Prior to biological evaluation, the cytotoxicity of the tested compounds was assessed using the MTS assay. None of the compounds exhibited significant cytotoxic effects at the highest concentrations tested (Fig. 3). The inhibitory effects on NO production were subsequently determined, revealing that most benzylresorcinol and benzylstilbene derivatives, including compounds 1–6 and 8–13, displayed notable activity with IC_50_ values ranging from 6.59 to 15.79 µM. Grossamide, a lignanamide previously reported to possess potent anti-neuroinflammatory activity,^30^ was employed as the positive control. Notably, compounds 6 (IC_50_ = 7.77 ± 0.24 µM), 10 (IC_50_ = 7.48 ± 0.57 µM), 11 (IC_50_ = 8.62 ± 0.86 µM), and 12 (IC_50_ = 6.59 ± 0.22 µM) exhibited stronger inhibitory effects on NO production in LPS-stimulated BV-2 microglial cells than grossamide (9.23 ± 0.05 µM), indicating their potential anti-neuroinflammatory activity (Table 3). The anti-neuroinflammatory activities observed in the present study were comparable to previous reports on structurally related natural products. In particular, benzylated stilbenes (parvifolins I–L) isolated from *Gnetum parvifolium* inhibited LPS-induced NO production in BV-2 microglial cells, with IC_50_ values ranging from 0.35 to 37.4 µM.^31^ The newly identified benzylstilbene derivatives 4–6 in the present study exhibited IC_50_ values ranging from 7.77 to 14.33 µM, which fell within the reported range and provided relevant context for the activities observed in this study.

**Fig. 3:**
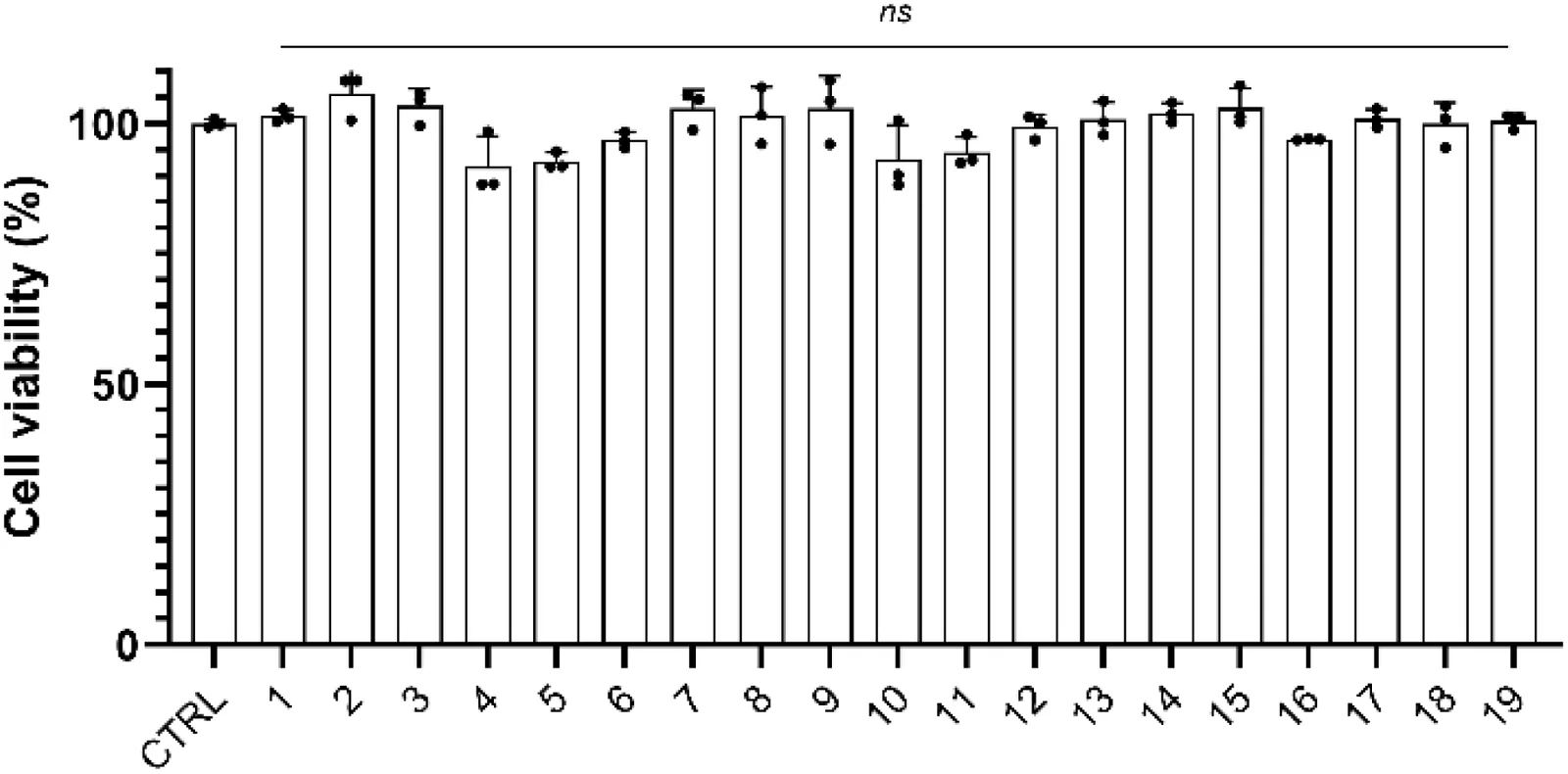
Cell viability of compounds 1–19.

**Table 3:** Inhibition of NO production of compounds 1–19

Compound	IC_50_ (µM)	Compound	IC_50_ (µM)
1	15.79 ± 0.27	11	8.62 ± 0.86
2	15.61 ± 0.06	12	6.59 ± 0.22
3	13.61 ± 0.79	13	10.44 ± 0.73
4	14.33 ± 0.30	14	> 20
5	12.76 ± 0.22	15	> 20
6	7.77 ± 0.24	16	> 20
7	> 20	17	> 20
8	12.32 ± 0.21	18	> 20
9	12.43 ± 0.47	19	> 20
10	7.48 ± 0.57	Grossamide	9.23 ± 0.05

Among compounds 6 and 10–12, compound 12 exhibited the strongest inhibition of NO production, with the lowest IC_50_ value of 6.59 ± 0.22 µM. It was therefore selected as the representative benzylstilbene derivative for subsequent immunofluorescence analyses of iNOS expression and NF-κB p65 nuclear translocation. By immunofluorescence staining, compound 12 markedly attenuated LPS-induced iNOS expression in BV-2 microglial cells in a concentration-dependent manner (Fig. 4A and C). In addition, compound 12 significantly suppressed the nuclear translocation of NF-κB p65, as evidenced by the reduced nuclear localization and decreased nuclear-to-cytoplasmic fluorescence intensity ratio compared with the LPS-treated group (Fig. 4B and D). These results suggest that the anti-inflammatory activity of compound 12 was associated with inhibition of NF-κB activation, leading to the downregulation of iNOS expression.

**Fig. 4:**
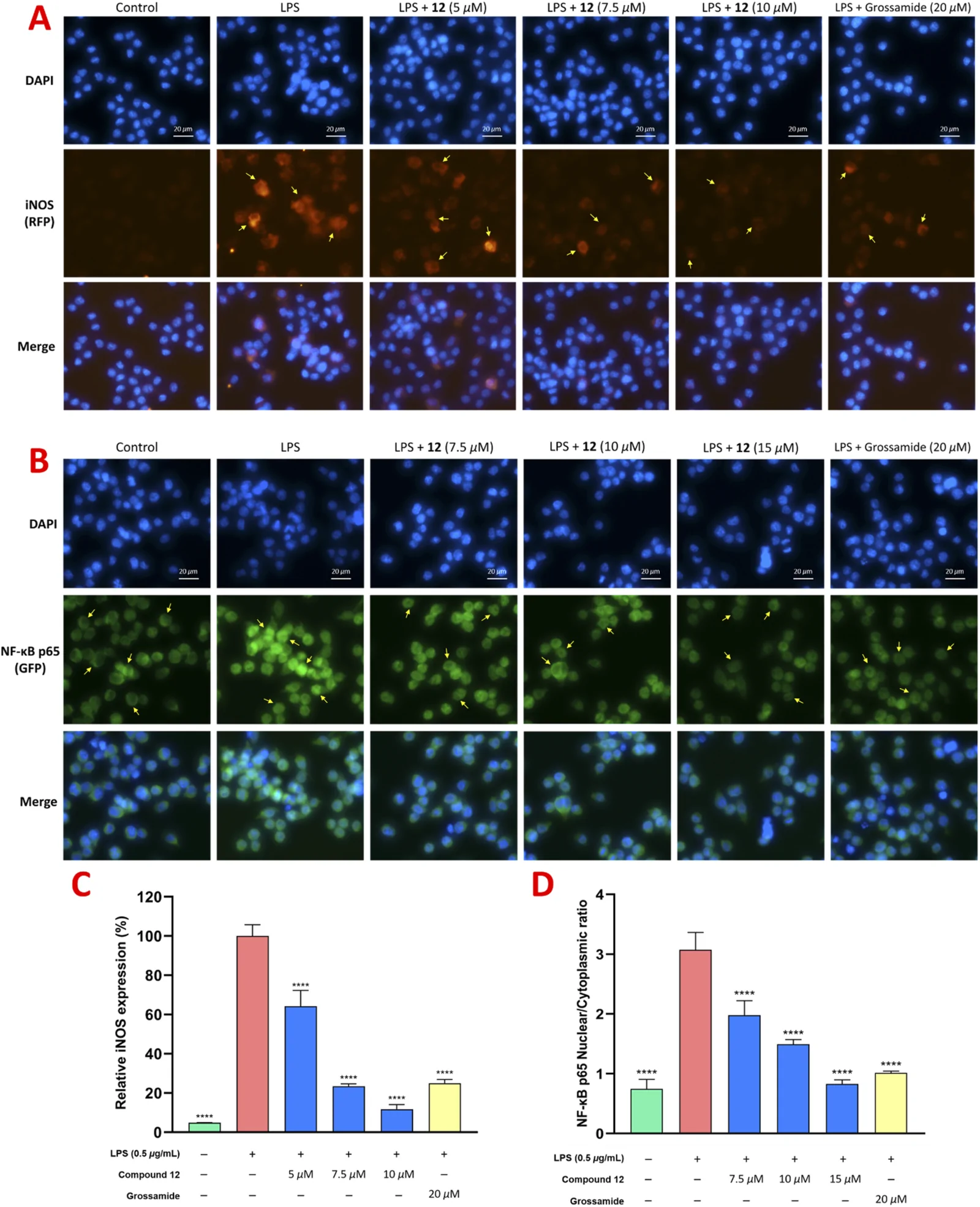
Immunofluorescence analysis of iNOS expression and NF-κB p65 nuclear translocation in LPS- induced BV-2 microglial cells. (A) Representative immunofluorescence images showing DAPI-stained nuclei (blue), iNOS (red), and merged images. (B) Representative immunofluorescence images showing DAPI-stained nuclei (blue), NF-κB p65 (green), and merged images. (C) Quantitative analysis of relative iNOS fluorescence intensity, normalized to the LPS-treated group. (D) Quantitative analysis of NF-κB p65 nuclear translocation expressed as the nuclear-to-cytoplasmic (N/C) fluorescence intensity ratio. Data are presented as mean ± SD (*n* = 3), with at least 30 cells analyzed per group, magnification ×40, scale bar = 20 µm. ****p < 0.0001 *versus* the LPS-treated group.

### Molecular docking analysis

Molecular docking studies were conducted using AutoDock 4.2.6 to investigate the binding interactions of the most active compounds (6, 10, 11, and 12) with iNOS and the IκBα/NF-κB complex. According to the simulation results shown in Table 4, all four compounds exhibited favorable predicted binding energies toward iNOS, ranging from −10.60 to −10.14 kcal mol^−1^, which were markedly lower than that of the reference inhibitor 1400 W (−7.34 kcal mol^−1^), suggesting favorable interactions with the enzyme. Likewise, docking analysis against the IκBα/NF-κB complex revealed binding energies ranging from −10.55 to −9.23 kcal mol^−1^ (Table 4), all of which were lower than that of the reference inhibitor BAY 11-7082 (−8.06 kcal mol^−1^), a well-established inhibitor of IκBα phosphorylation and NF-κB activation.^32^ These results indicated that the benzylstilbene derivatives from the fruits of *G. nudifolia* possess favorable binding characteristics toward both inflammatory targets. As docking scores primarily reflect predicted ligand–protein interactions under simplified *in silico* conditions, the docking analysis was used to qualitatively examine possible binding modes and ligand–protein interactions and to provide structural context for the experimentally observed activities. Within this framework, compound 12 exhibited the most favorable predicted binding energies toward both iNOS and the IκBα/NF-κB complex and also showed the strongest inhibition of NO production. Detailed analysis of the docking pose revealed that compound 12 formed conventional hydrogen bonds with Arg-A199 (6.43 Å), Gly-A202 (2.95 Å), and Glu-A377 (5.19 Å) through the hydroxyl groups located at C-5, C-2′, and C-4″, respectively. In addition, several π-mediated interactions were observed between methoxy group and residues Met-A374 (3.65 Å), Met-A434 (3.82 Å), and Ala-A439 (4.39 Å), as well as between the *ortho*-hydroxy substituted phenyl ring of the stilbene moiety and Pro-A350 (5.12 Å) and Val-A352 (6.68 Å). Furthermore, the aromatic ring of the 2″-benzyl unit participated in multiple hydrophobic and π-based interactions with Cys-A200 (6.21 Å), Ile-A201 (5.12 Å), Met-374 (4.58 Å), and Glu-A377 (5.98 Å). These interactions collectively contributed to the stable accommodation of compound 12 within the iNOS binding pocket. Notably, comparison with the docking pose of 1400 W, a highly selective iNOS inhibitor,^33^ revealed that compound 12 shared several key interacting residues, including Glu-A377, Cys-A200, Trp-A372, Pro-A350, and Val-A352. Furthermore, analysis of the docking interactions (Fig. 5 and Table S1) revealed that Arg199, Cys200, Ile201, Gly202, Glu377, and Met434 were the key amino acid residues involved in the binding of the benzylstilbene derivatives (compound 6, 10, 11, and 12) within the active site of iNOS.

**Table 4:** Binding energy (kcal mol^−1^) of compound 6, 10, 11, 12, 1400 W, and BAY 11-7082 with iNOS and IκBα/NF-κB proteins

Ligand	iNOS (PDB ID: 1NSI)	IκBα/NF-κB complex (PDB ID: 1IKN)
Compound 6	−10.32	−9.23
Compound 10	−10.14	−9.93
Compound 11	−10.43	−10.17
Compound 12	−10.60	−10.55
1400 W	−7.34	—
BAY 11-7082	—	−8.06

**Fig. 5:**
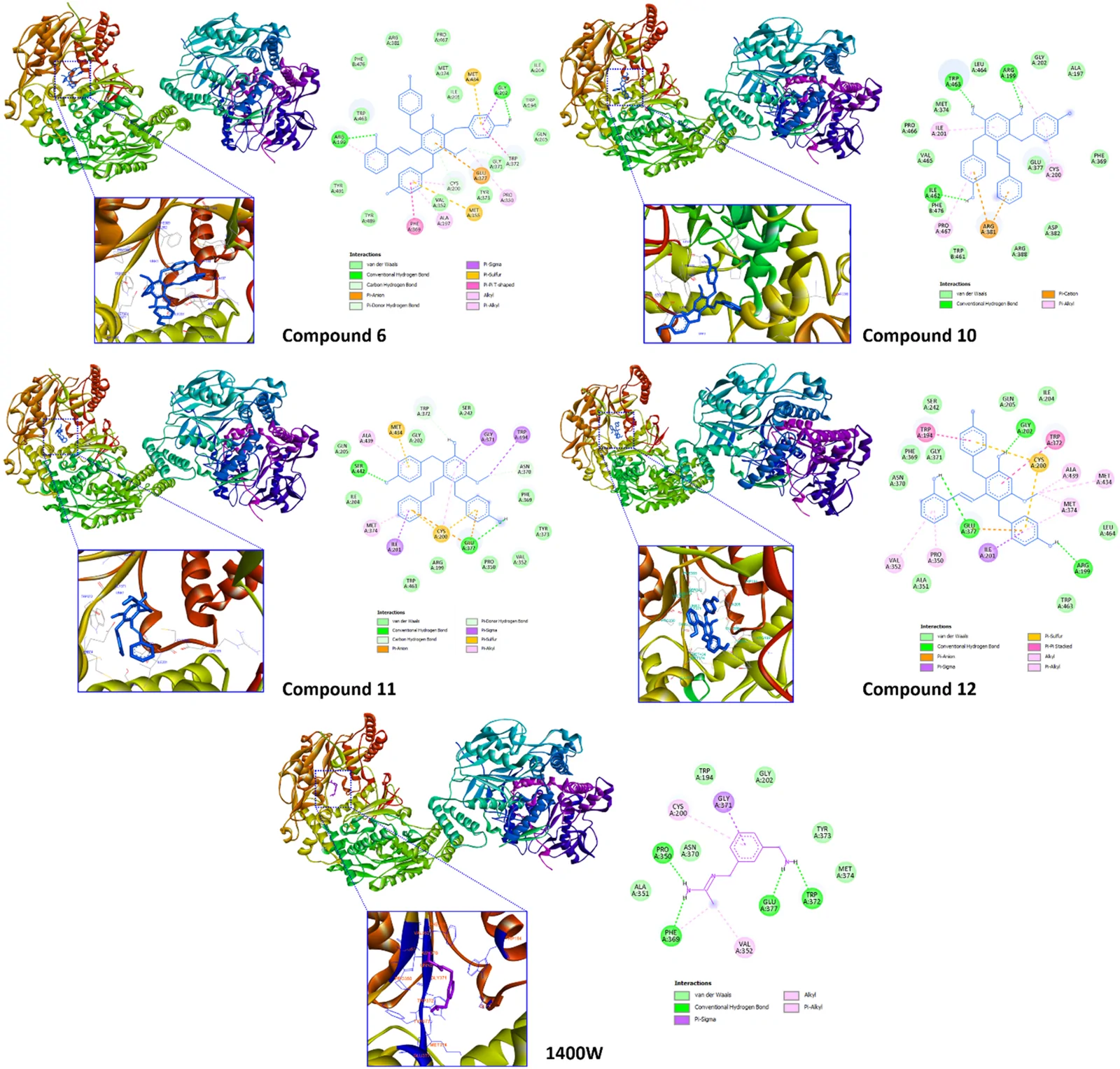
The 3D and 2D molecular interactions of compound 6, 10, 11, 12, and 1400 W with iNOS (PDB ID: 1NSI).

The IκBα in complex with the NF-κB p50/p65 heterodimer (PDB ID: 1IKN) was selected for molecular docking. Obtained results revealed that compound 12 formed four hydrogen bonds with residues Asp-A223 (3.07 Å), Gly-A237 (3.80 Å), Gly-D259 (3.94 Å), and Arg-D260 (5.21 Å) through phenolic hydroxyl groups at C-5, C-2′, C-4″, and C-4‴, respectively. In addition, the ligand established several hydrophobic and π-mediated interactions, including π-anion interactions with Glu-A225 (4.02 Å and 6.05 Å) and π-alkyl interactions with Arg-A50 (5.96 Å), Arg-A236 (5.22 Å), Phe-A239 (5.01 Å), Pro-A275 (5.99 Å), and Pro-D261 (5.60 Å). These interactions were primarily associated with the extended aromatic π-system of the benzylstilbene scaffold. The 1IKN structure consists of three subunits: NF-κB p65 (chain A), NF-κB p50 (chain C), and IκBα (chain D). Notably, all interactions between compound 12 and the protein complex were localized to chains A and D, corresponding to the p65 and IκBα subunits, respectively. Structural studies have shown that the p65–IκBα interface involves residues such as Arg-A50, Asp-A223, and Glu-A225 of p65, which interact with the backbone region surrounding Gly-D259 of IκBα.^34^ Interestingly, compound 12 simultaneously interacted with several of these interface residues, suggesting that the ligand occupies a region associated with the p65–IκBα binding interface. In addition, compounds 6, 10, 11, and 12 shared predicted interactions with Arg-A50, Arg-A236, Gly-A237, Gly-D259, Arg-D260, and Pro-D261 (Fig. 6 and Table S1).

**Fig. 6:**
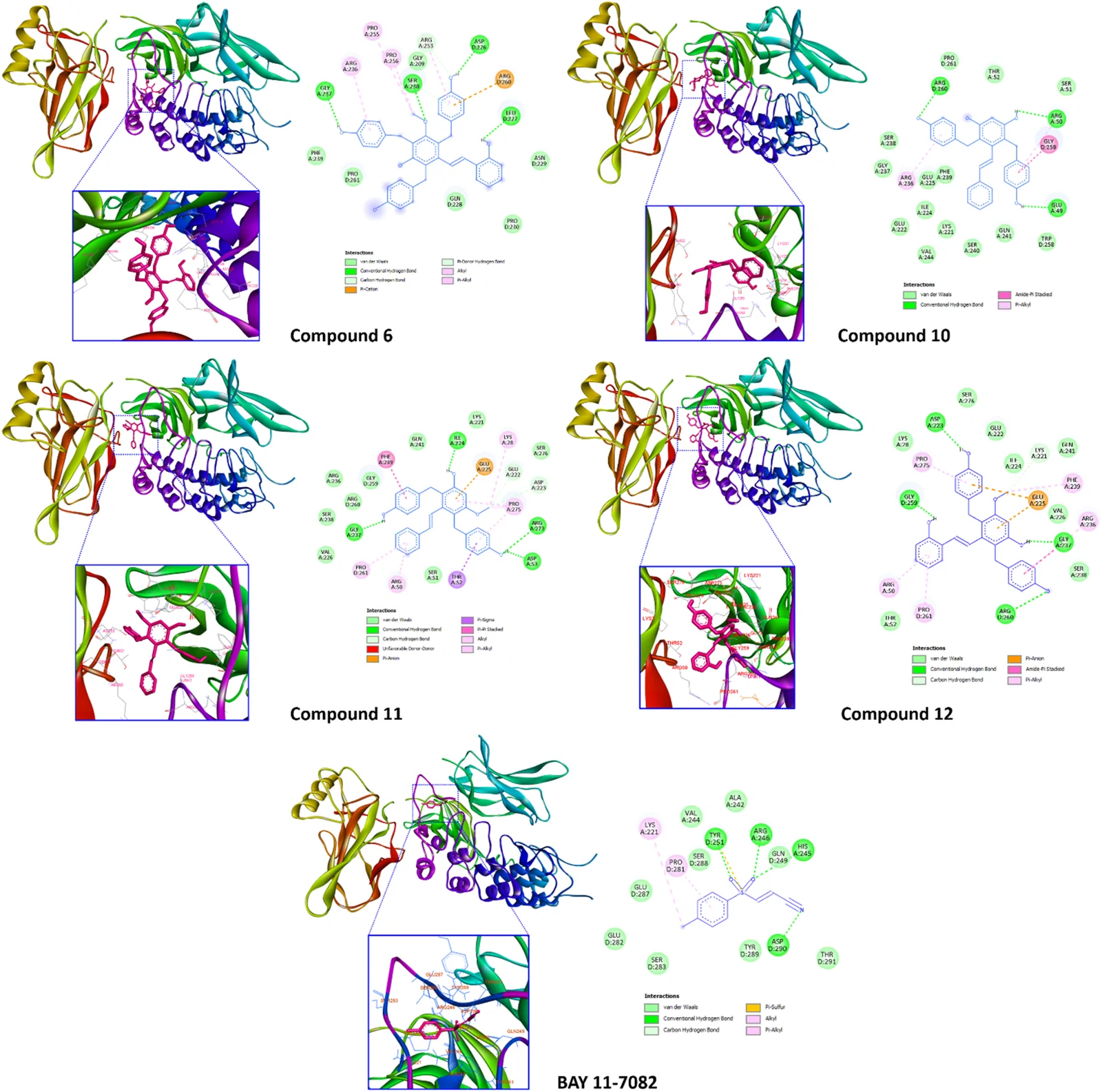
The 3D and 2D molecular interactions of compound 6, 10, 11, 12, and BAY 11-7082 with IκBα/NF-κB complex (PDB ID: 1IKN).

### Structure–activity relationship (SAR) analysis

Comparison of the structures and NO-inhibitory activities of compounds 1–13 revealed several structural features associated with their activities. Using the central aromatic ring of compound 6 as the common numbering reference, compounds 6 and 10–12, possessing both a *trans*-styryl substituent at C-1 and a *para*-hydroxybenzyl substituent at C-6, exhibited the strongest activities, with IC_50_ values ranging from 6.59 to 8.62 µM. Compounds 4 and 7–9 lack a *para*-hydroxybenzyl substituent at C-6 and showed moderate or weak activity, with IC_50_ values of 12.32–14.33 µM or greater than 20 µM. Compounds 1–3, bearing a linear alkyl substituent at C-1, also showed relatively weak activity, with IC_50_ values ranging from 13.61 to 15.79 µM. However, compounds 5 and 13 possess both principal features but displayed lower activities, with IC_50_ values of 12.76 and 10.44 µM, respectively, suggesting that substitution at the meta position of the terminal phenyl ring of the *trans*-styryl moiety may further modulate NO-inhibitory activity. In addition, docking models predicted several π-mediated interactions involving the *trans*-styryl and *para*-hydroxybenzyl moieties with iNOS and the IκBα/NF-κB complex (Fig. 5 and 6), providing qualitative structural context for these SAR observations (Fig. 7).

**Fig. 7:**
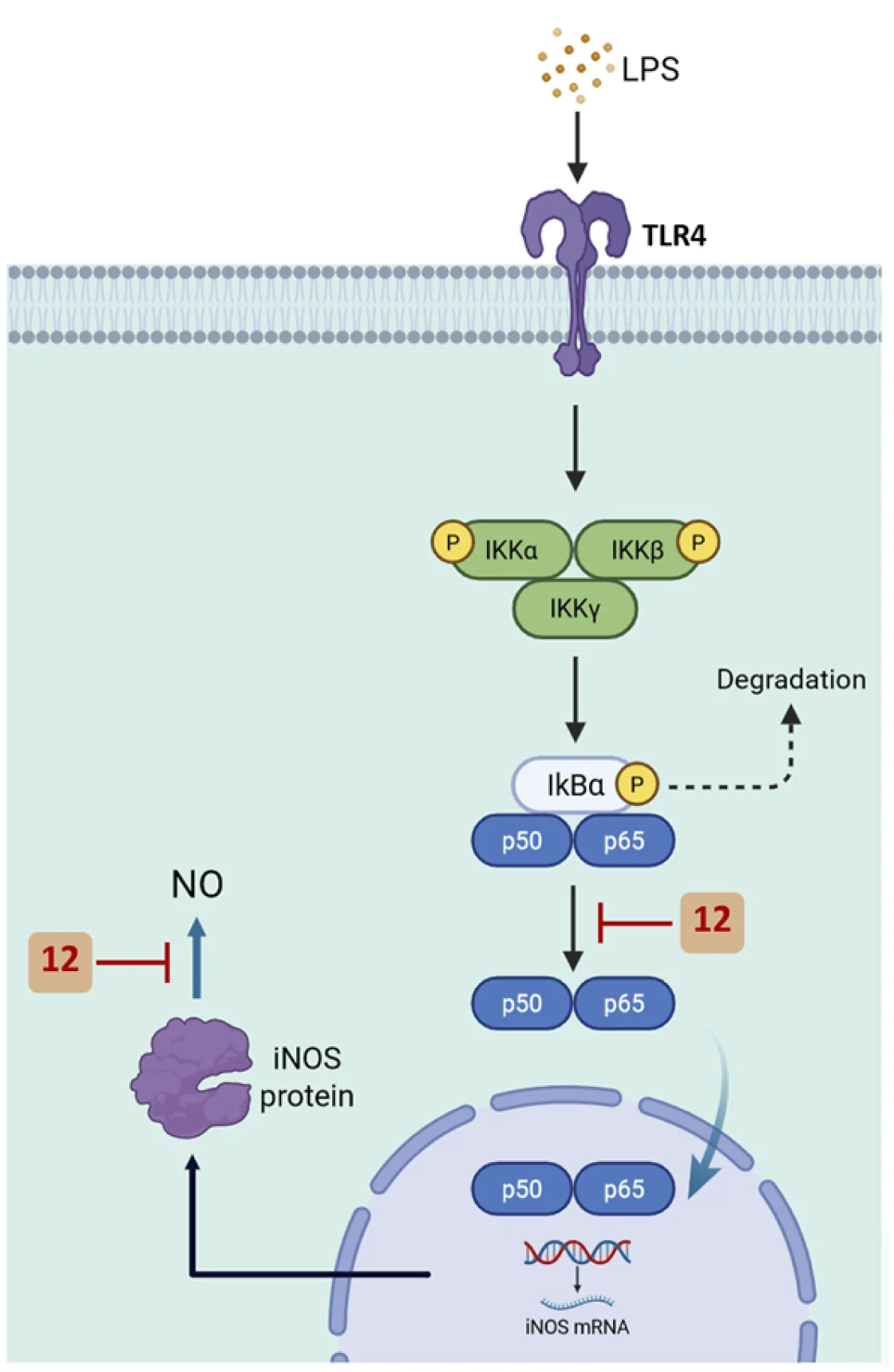
The anti-neuroinflammatory mechanism of benzylstilbene derivatives from the fruits of *G. nudifolia*.

## Conclusions

In conclusion, using a bioassay-guided approach, nineteen compounds, including six previously undescribed compounds (1–6) and thirteen known compounds (7–19), were isolated and structurally characterized from the fruits of *Galeola nudifolia.* Evaluation of their anti-neuroinflammatory activity in LPS-stimulated BV-2 microglial cells revealed that most benzylresorcinol and benzylstilbene derivatives (1–6 and 8–13) effectively inhibited NO production, with IC_50_ values ranging from 6.59 to 15.79 µM. Among them, compounds 6, 10, 11, and 12 exhibited greater inhibitory potency than the positive control (grossamide) with IC_50_ values of 7.77 ± 0.24, 7.48 ± 0.57, 8.62 ± 0.86, and 6.59 ± 0.22 µM, respectively. Furthermore, mechanistic investigations combining immunofluorescence assays and molecular docking analyses suggested that the anti-neuroinflammatory activities of these benzylstilbene derivatives are associated with suppression of iNOS expression and modulation of the NF-κB signaling pathway. Collectively, these findings expand the known chemical diversity of *G. nudifolia* and identify its benzylstilbene derivatives as natural products with anti-neuroinflammatory activity that warrant further pharmacological investigation.

## Author contributions

Duy-Hien Tran: conceptualization, data curation, formal analysis, investigation, methodology, validation, visualization, writing – original draft, writing – review & editing. Bien-Thuy Bui Nguyen: investigation, data curation. Yuh-Chiang Shen: methodology, investigation. Chia-Ching Liaw: analysis of results, methodology, spectroscopic data interpretation, writing – review & editing. Chih-Hua Chao: analysis of results, writing – review & editing. Hui Chi Huang: software. I-Wen Lo: writing – review & editing. Chin-Yeu Chen: methodology. Mei-Hsien Lee: conceptualization, methodology, supervision, analysis of results, visualization, writing – review & editing. Yu-Chi Lin: conceptualization, methodology, resources, project administration, supervision, analysis of results, writing – review & editing.

## Conflicts of interest

There are no conflicts to declare.

## Supplementary Material

RA-016-D6RA06075G-s001

## Data Availability

The data that support the findings of this study are available in the supplementary information (SI). Supplementary information: biological activities of crude extract, fractions, and subfractions; spectral data of new compounds 1–6; HPLC profiles of new isolated compounds; and molecular docking interactions of compounds 6 and 10–12, 1400 W, and BAY 11-7082 with iNOS and IκBα/NF-κB proteins. See DOI: https://doi.org/10.1039/d6ra06075g.
