# Supplementary material for: New benzylresorcinol and benzylstilbene derivatives from the fruits of orchid Galeola nudifolia Lour. with anti-neuroinflammatory activity
Source: RSC Adv. 2026 Oct 5;16(46):60130–42. doi: 10.1039/d6ra06075g (PMC13636293; doi:10.1039/d6ra06075g)
Supplement: Supplementary file 1 [file RA-016-D6RA06075G-s001.pdf]

Supplementary Information (SI) for RSC Advances.
This journal is © The Royal Society of Chemistry

SUPPORTING INFORMATION

New benzylresorcinol and benzylstilbene derivatives from the fruits of orchid *Galeola nudifolia* with anti-neuroinflammatory activity

Duy-Hien Tran,[abc] Bien-Thuy Bui Nguyen,[bd] Yuh-Chiang Shen,[b] Chia-Ching Liaw,[bef] Chih-Hua Chao,[gh] Hui-Chi Huang,[i] I-Wen Lo,[b] Chin-Yeu Chen,[j] Mei-Hsien Lee,[*ak] and Yu-Chi Lin[*b]

[a] *Ph.D. Program in Clinical Drug Development of Herbal Medicine, College of Pharmacy, Taipei Medical University, Taipei 11031, Taiwan. E-mail: lmh@tmu.edu.tw*

[b] *National Research Institute of Chinese Medicine, MOHW, Taipei 112026, Taiwan. E-mail: yclin@nricm.edu.tw.*

[c] *School of Pharmacy, University of Medicine and Pharmacy at Ho Chi Minh City, Ho Chi Minh City 700000, Vietnam*

[d] *University of Health Sciences, Vietnam National University Ho Chi Minh City, Ho Chi Minh City 700000, Vietnam*

[e] *Department of Pharmacy, School of Pharmaceutical Sciences, National Yang Ming Chiao Tung University, Taipei 112, Taiwan*

[f] *Graduate Institute of Natural Products, Kaohsiung Medical University, Kaohsiung 807, Taiwan*

[g] *School of Pharmacy, China Medical University, Taichung 406040, Taiwan*

[h] *Chinese Medicine Research and Development Center, China Medical University Hospital, Taichung 404327, Taiwan*

[i] *School of Chinese Medicine & Graduate Institute of Chinese Medicine, China Medical University, Taichung 406040, Taiwan*

[j] *Department of Physical Therapy, Tzu-Hui Institute of Technology, Pingtung 92641, Taiwan*

[k] *Graduate Institute of Pharmacognosy, College of Pharmacy, Taipei Medical University, Taipei 11031, Taiwan*

Fig. S1. Cell viability (A) and inhibition of NO production (B) in LPS-induced BV-2 cells of crude extract and fractions 4
Fig. S2. Cell viability (A) and inhibition of NO production (B) in LPS-induced BV-2 cells of *n*-hexane subfractions A1 – A12 4
Fig. S3. Cell viability (A) and inhibition of NO production (B) in LPS-induced BV-2 cells of ethyl acetate subfractions B1 – B9 4
Fig. S4. The HR-ESIMS (positive mode) spectrum of compound **1** 5
Fig. S5. The UV spectrum of compound **1** 5
Fig. S6. The ^{1}H NMR spectrum of compound **1** 6
Fig. S7. The ^{13}C NMR spectrum of compound **1** 6
Fig. S8. The HSQC spectrum of compound **1** 7
Fig. S9. The HMBC spectrum of compound **1** 7
Fig. S10. The COSY spectrum of compound **1** 8
Fig. S11. The HR-ESIMS (positive mode) spectrum of compound **2** 8
Fig. S12. The UV spectrum of compound **2** 9
Fig. S13. The ^{1}H NMR spectrum of compound **2** 9
Fig. S14. The ^{13}C NMR spectrum of compound **2** 10
Fig. S15. The HSQC spectrum of compound **2** 10
Fig. S16. The HMBC spectrum of compound **2** 11
Fig. S17. The COSY spectrum of compound **2** 11
Fig. S18. The HR-ESIMS (positive mode) spectrum of compound **3** 12
Fig. S19. The UV spectrum of compound **3** 12
Fig. S20. The ^{1}H NMR spectrum of compound **3** 13
Fig. S21. The ^{13}C NMR spectrum of compound **3** 13
Fig. S22. The HSQC spectrum of compound **3** 14
Fig. S23. The HMBC spectrum of compound **3** 14
Fig. S24. The COSY spectrum of compound **3** 15
Fig. S25. The HR-ESIMS (positive mode) spectrum of compound **4** 16
Fig. S26. The UV spectrum of compound **4** 16
Fig. S27. The ^{1}H NMR spectrum of compound **4** 16
Fig. S28. The ^{13}C NMR spectrum of compound **4** 17
Fig. S29. The HSQC spectrum of compound **4** 18
Fig. S30. The HMBC spectrum of compound **4** 18
Fig. S31. The COSY spectrum of compound **4** 19
Fig. S32. The HR-ESIMS (positive mode) spectrum of compound **5** 20
Fig. S33. The UV spectrum of compound **5** 20

Fig. S34. The ^{1}H NMR spectrum of compound **5** 20
Fig. S35. The ^{13}C NMR spectrum of compound **5** 21
Fig. S36. The HSQC spectrum of compound **5** 21
Fig. S37. The HMBC spectrum of compound **5** 22
Fig. S38. The COSY spectrum of compound **5** 22
Fig. S39. The HR-ESIMS (positive mode) spectrum of compound **6** 23
Fig. S40. The UV spectrum of compound **6** 23
Fig. S41. The ^{1}H NMR spectrum of compound **6** 24
Fig. S42. The ^{1}H NMR spectrum of compound **6** (range of 6.5 – 7.1 ppm) 24
Fig. S43. The ^{13}C NMR spectrum of compound **6** 25
Fig. S44. The ^{13}C NMR spectrum of compound **6** (range of 115 – 135 ppm) 25
Fig. S45. The HSQC spectrum of compound **6** 26
Fig. S46. The HMBC spectrum of compound **6** 27
Fig. S47. The COSY spectrum of compound **6** 27
Fig. S48. The HPLC-PDA profiles of the *n*-hexane layer (A), EtOAc layer (B), and six new compounds (C) 28

Table S1. Molecular docking interactions of compound **6**, **10**, **11**, **12**, 1400W, and BAY 11-7082 with iNOS and IκBα/NF-κB proteins 29

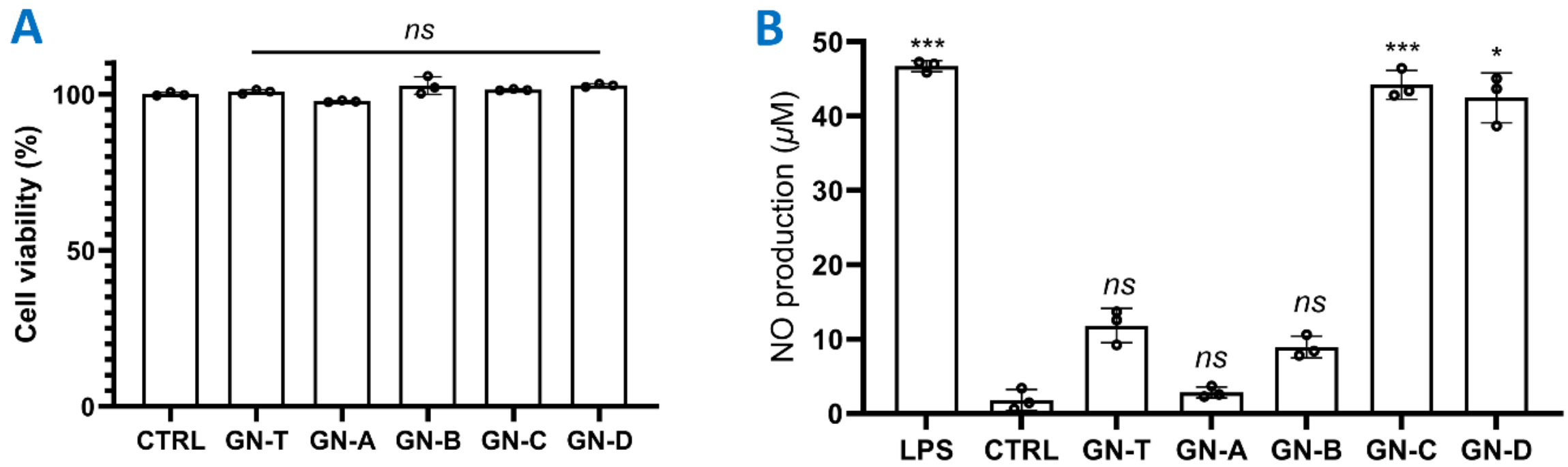

(GN-T: crude extract, GN-A: n-hexane fraction, GN-B: ethyl acetate fraction, GN-C: n-butanol fraction, GN-D: water fraction)

Fig. S1. Cell viability (A) and inhibition of NO production (B) in LPS-induced BV-2 cells of crude extract and fractions

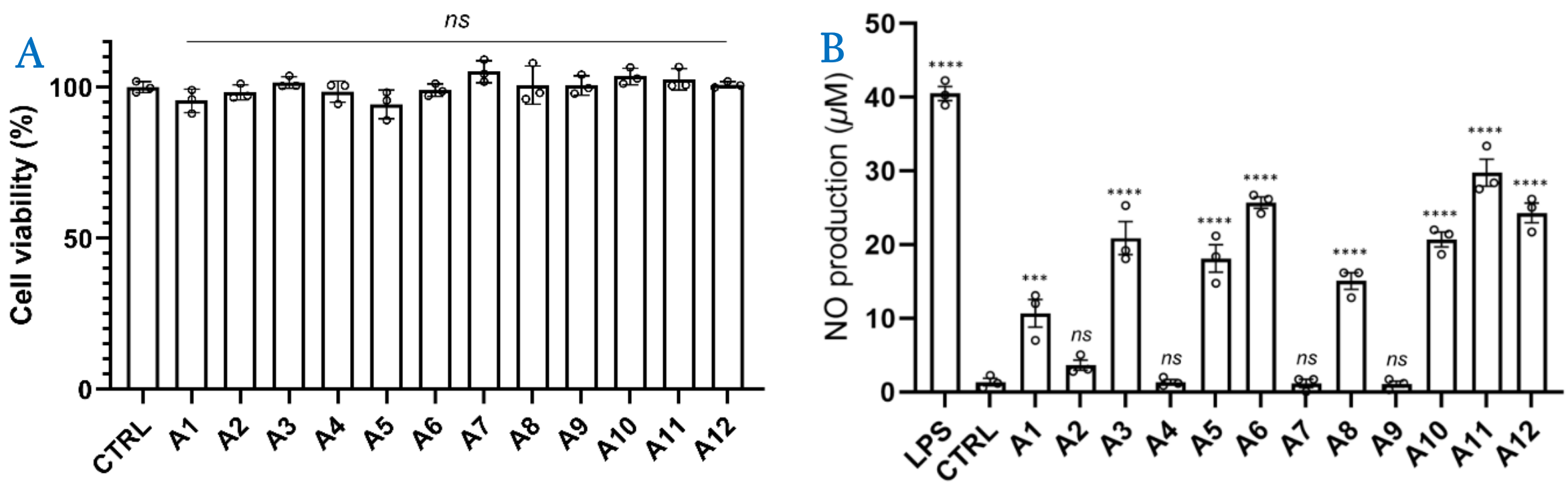

Fig. S2. Cell viability (A) and inhibition of NO production (B) in LPS-induced BV-2 cells of *n*-hexane subfractions A1 – A12

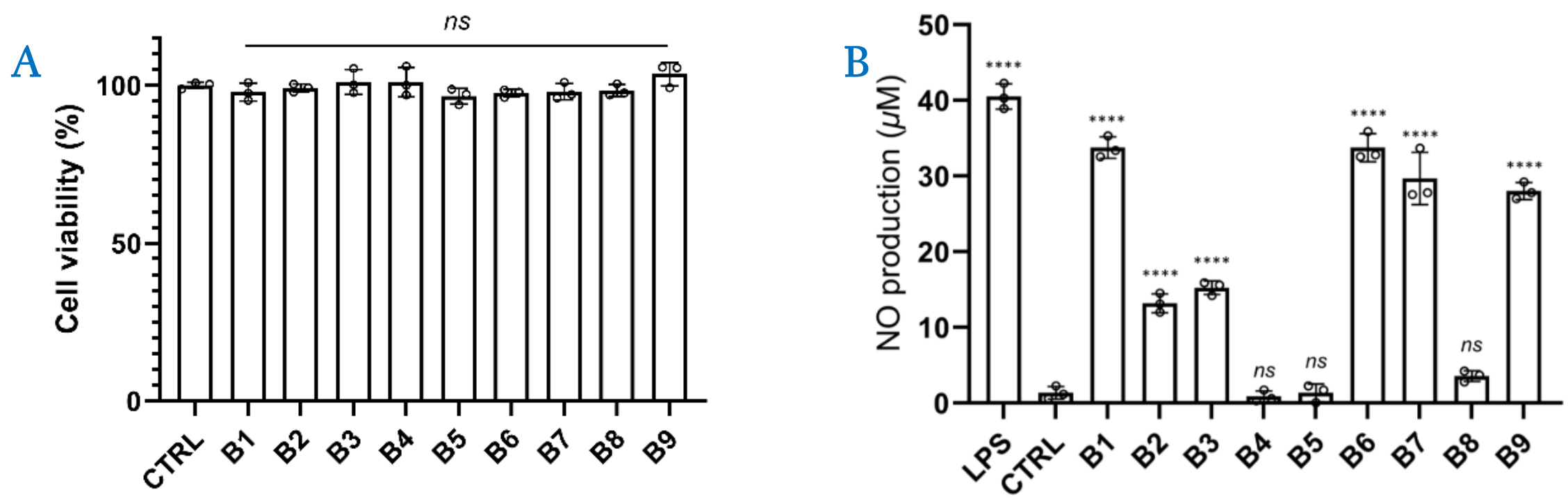

Fig. S3. Cell viability (A) and inhibition of NO production (B) in LPS-induced BV-2 cells of ethyl acetate subfractions B1 – B9

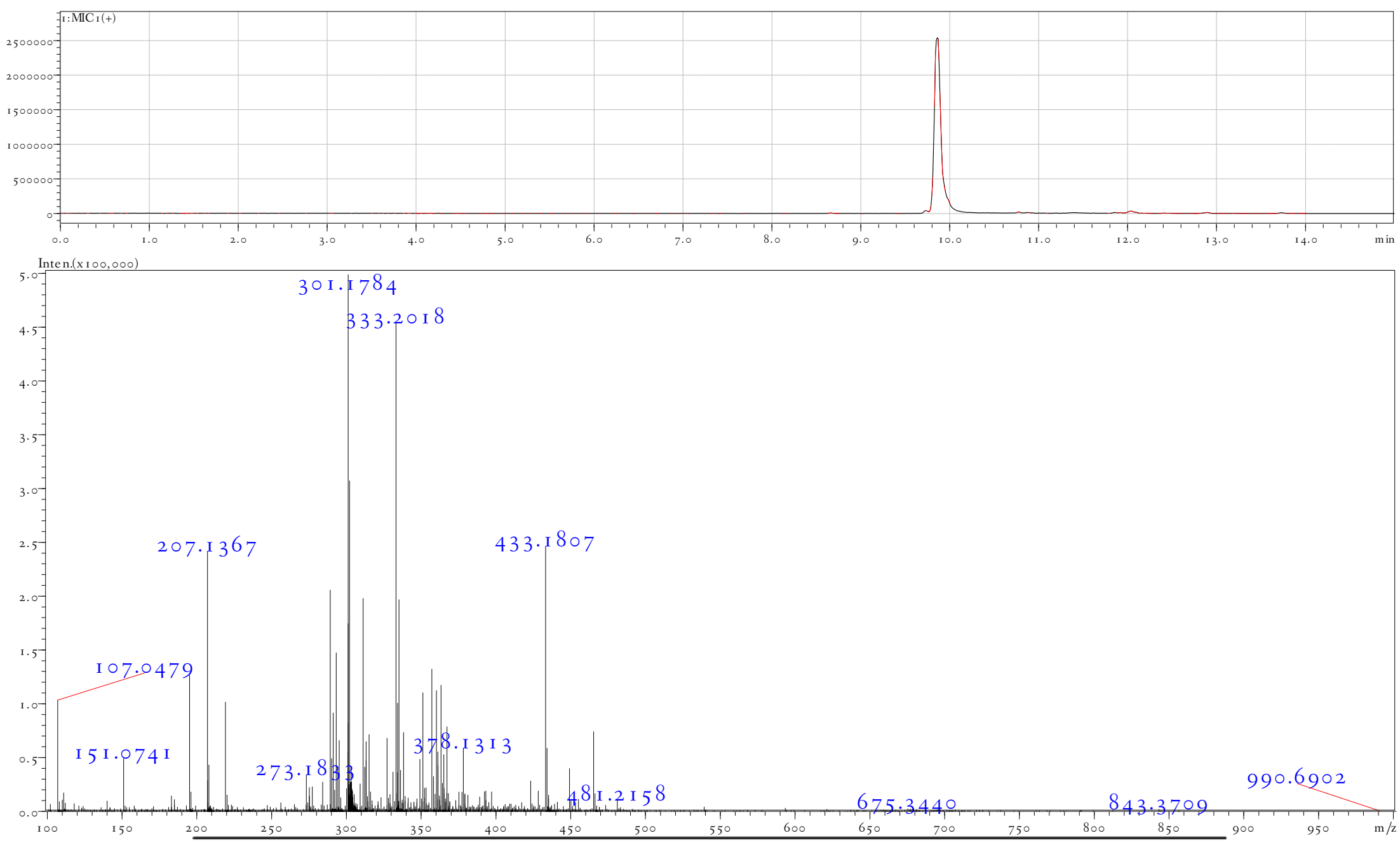

Molecular	$[M+H]^+$, m/z	Cald., m/z	Error (ppm)
$C_{19}H_{24}O_3$	301.1784	301.1798	4.7

Fig. S4. The HR-ESIMS (positive mode) spectrum of compound 1

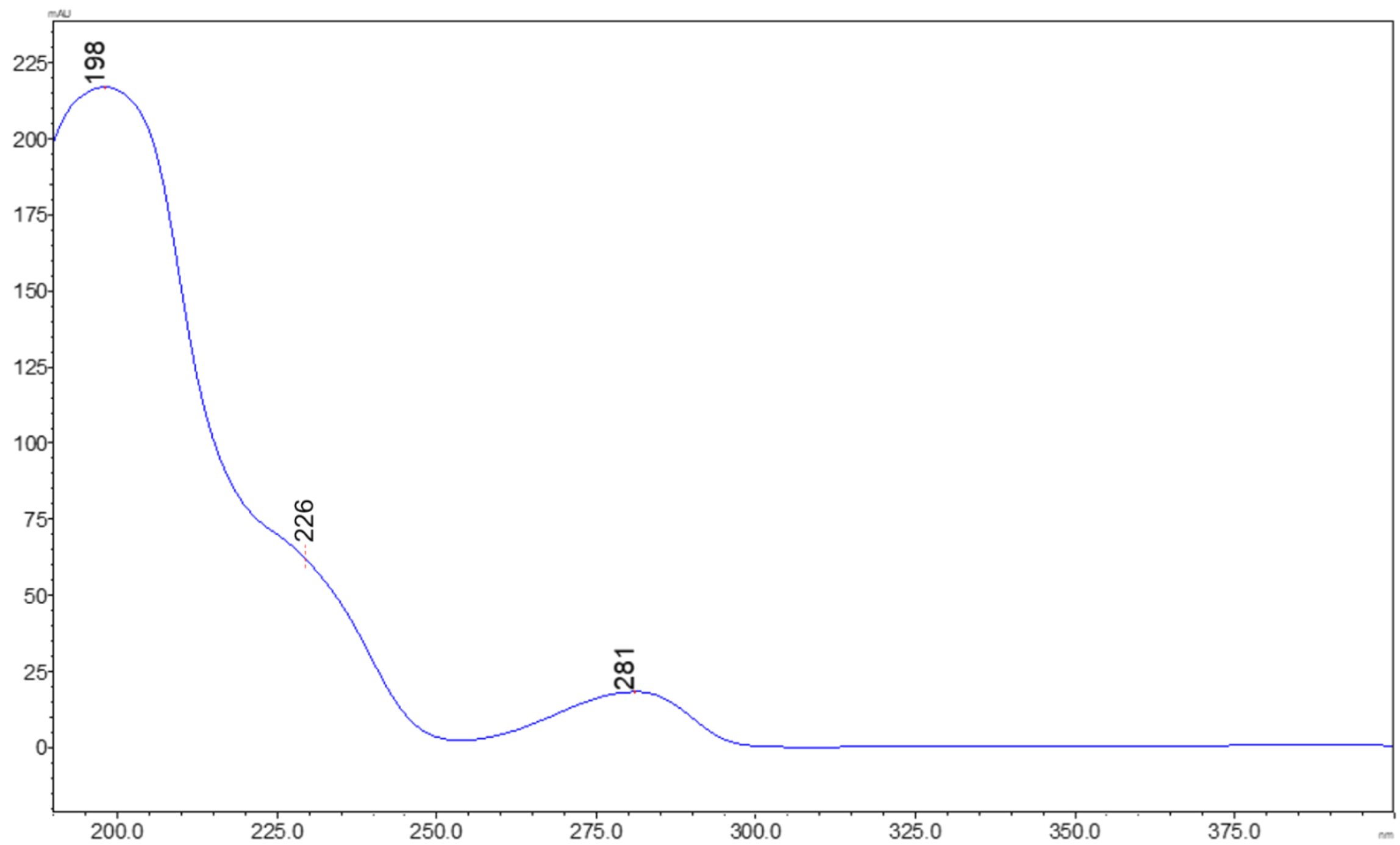

Fig. S5. The UV spectrum of compound 1

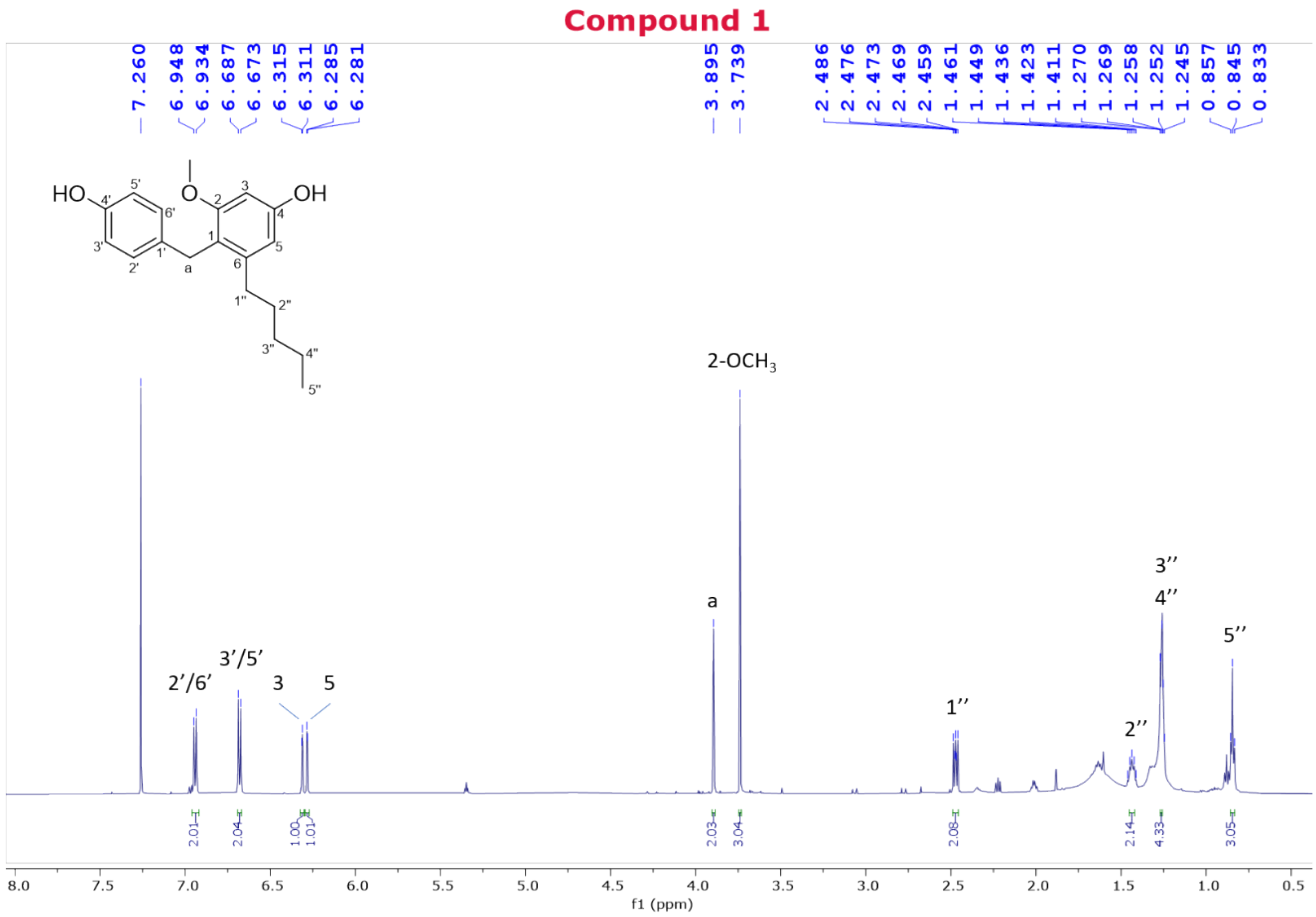

Fig. S6. The ^{1}H NMR spectrum of compound **1**

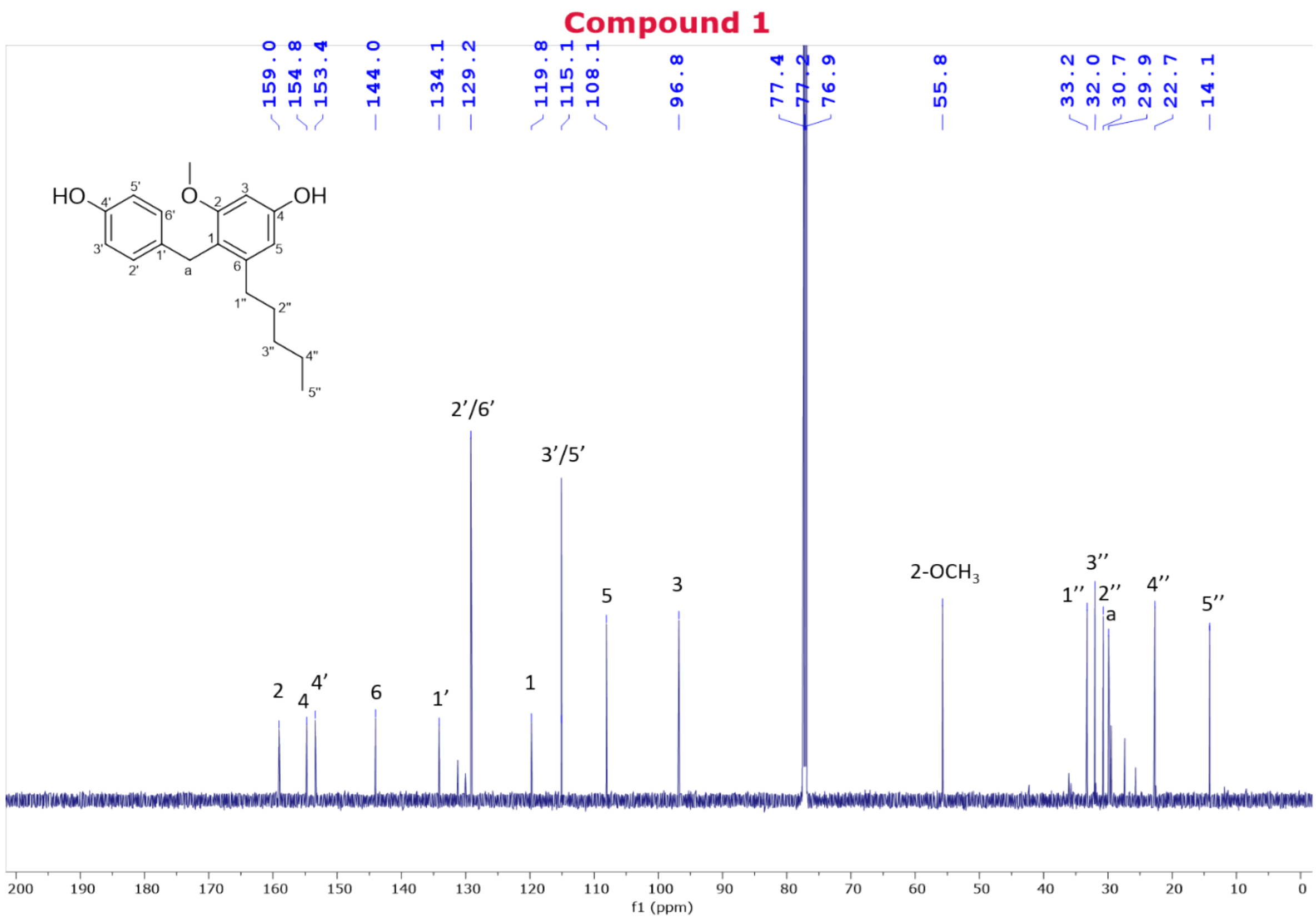

Fig. S7. The ^{13}C NMR spectrum of compound **1**

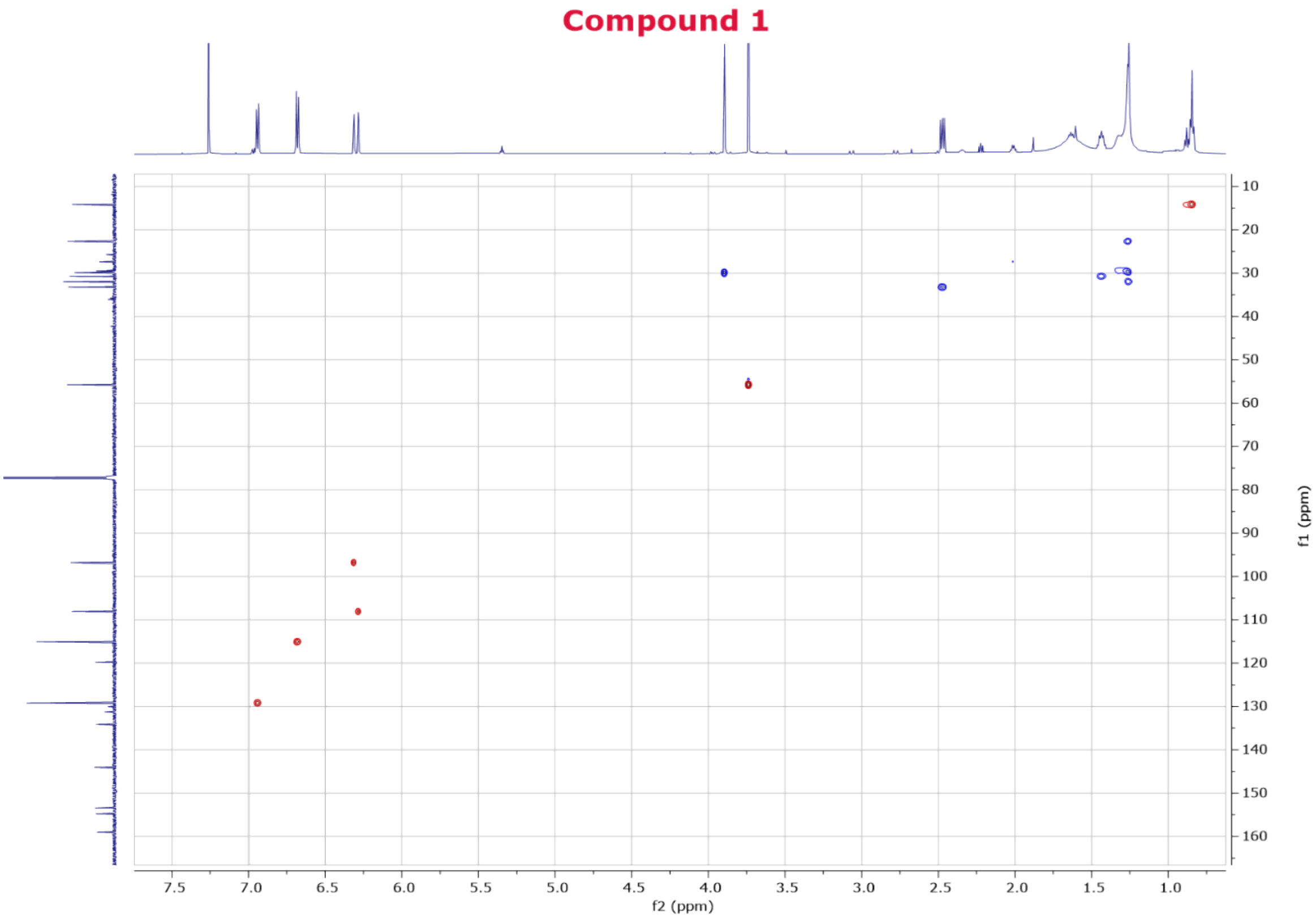

Fig. S8. The HSQC spectrum of compound 1

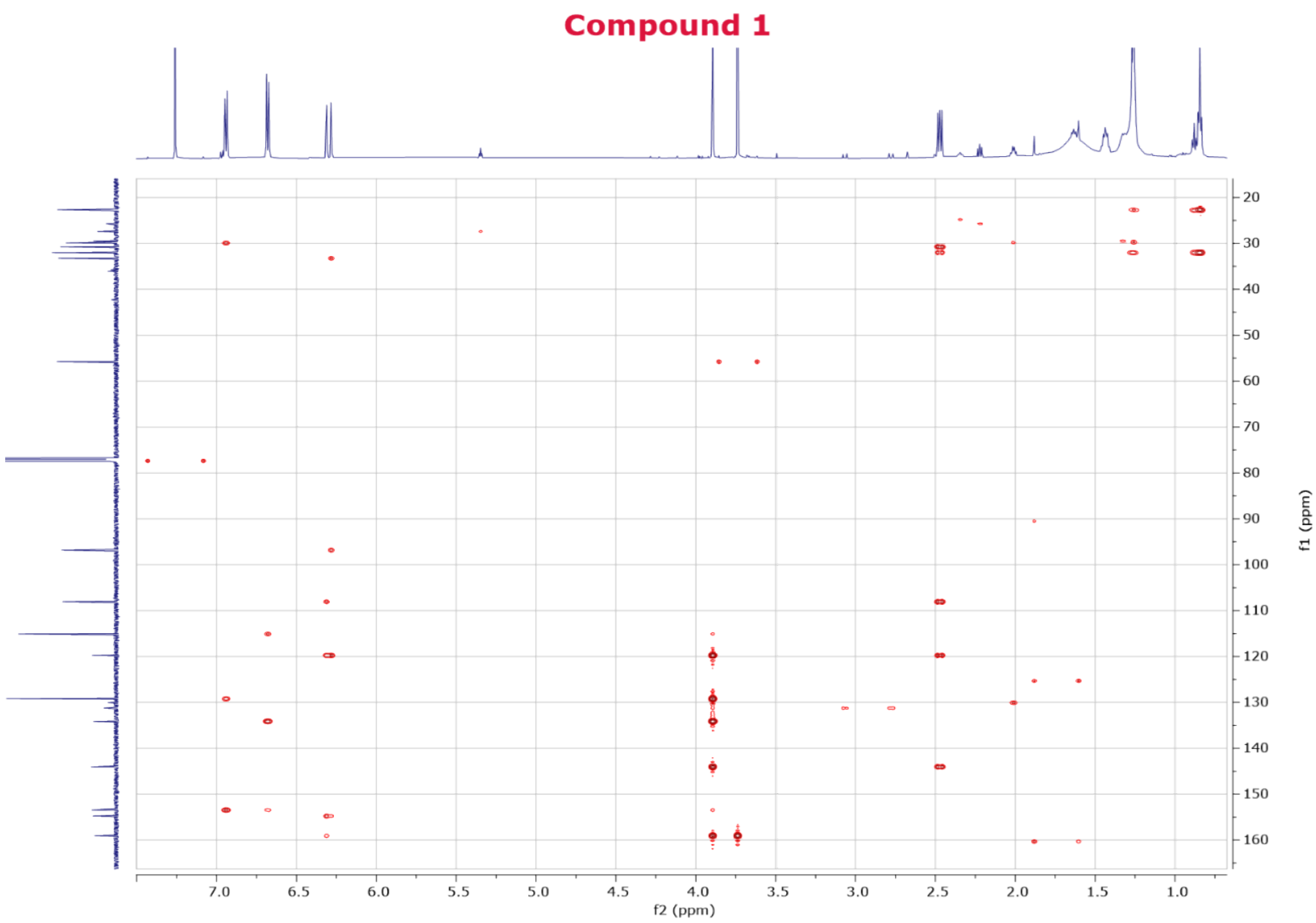

Fig. S9. The HMBC spectrum of compound 1

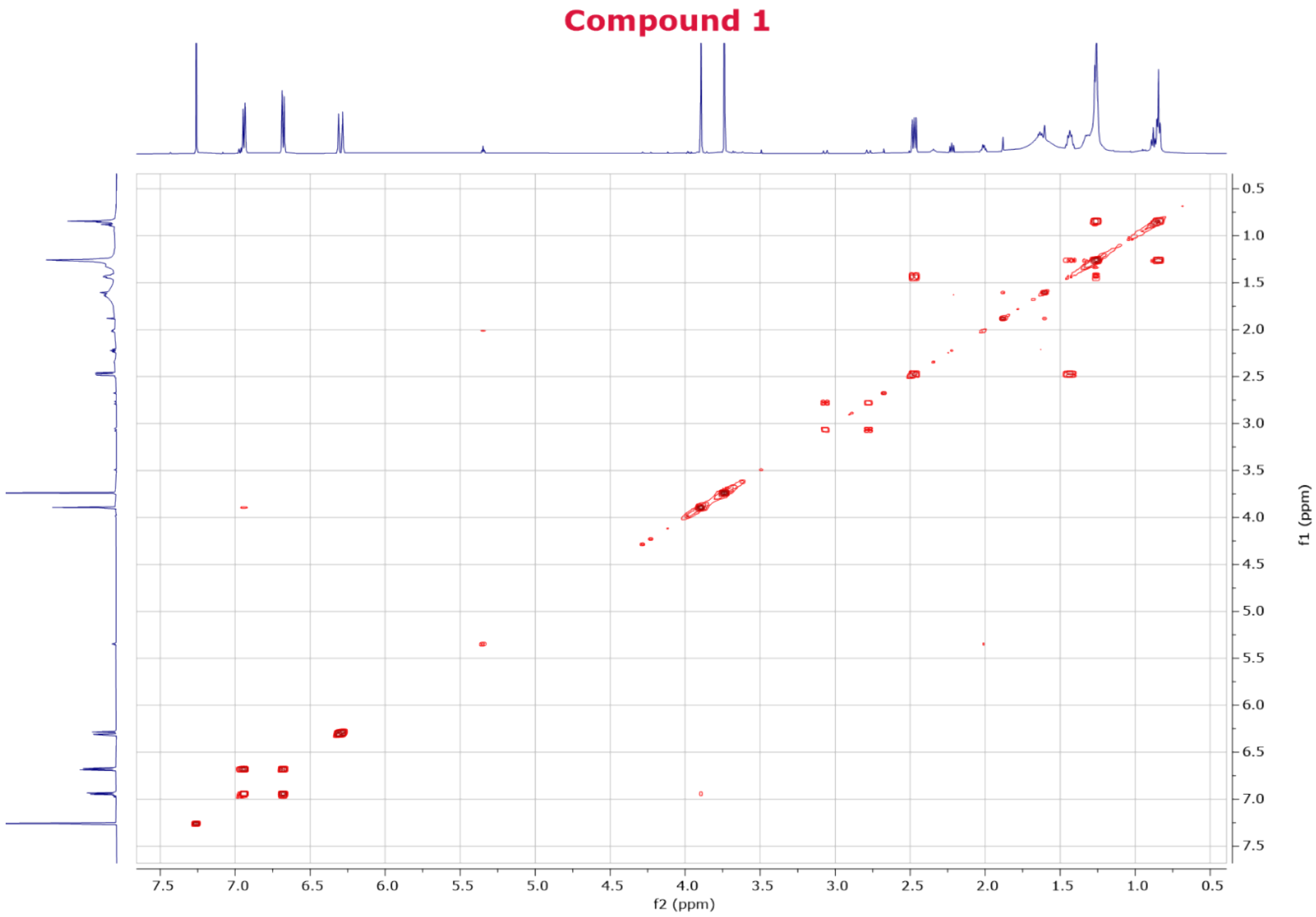

Fig. S10. The COSY spectrum of compound 1

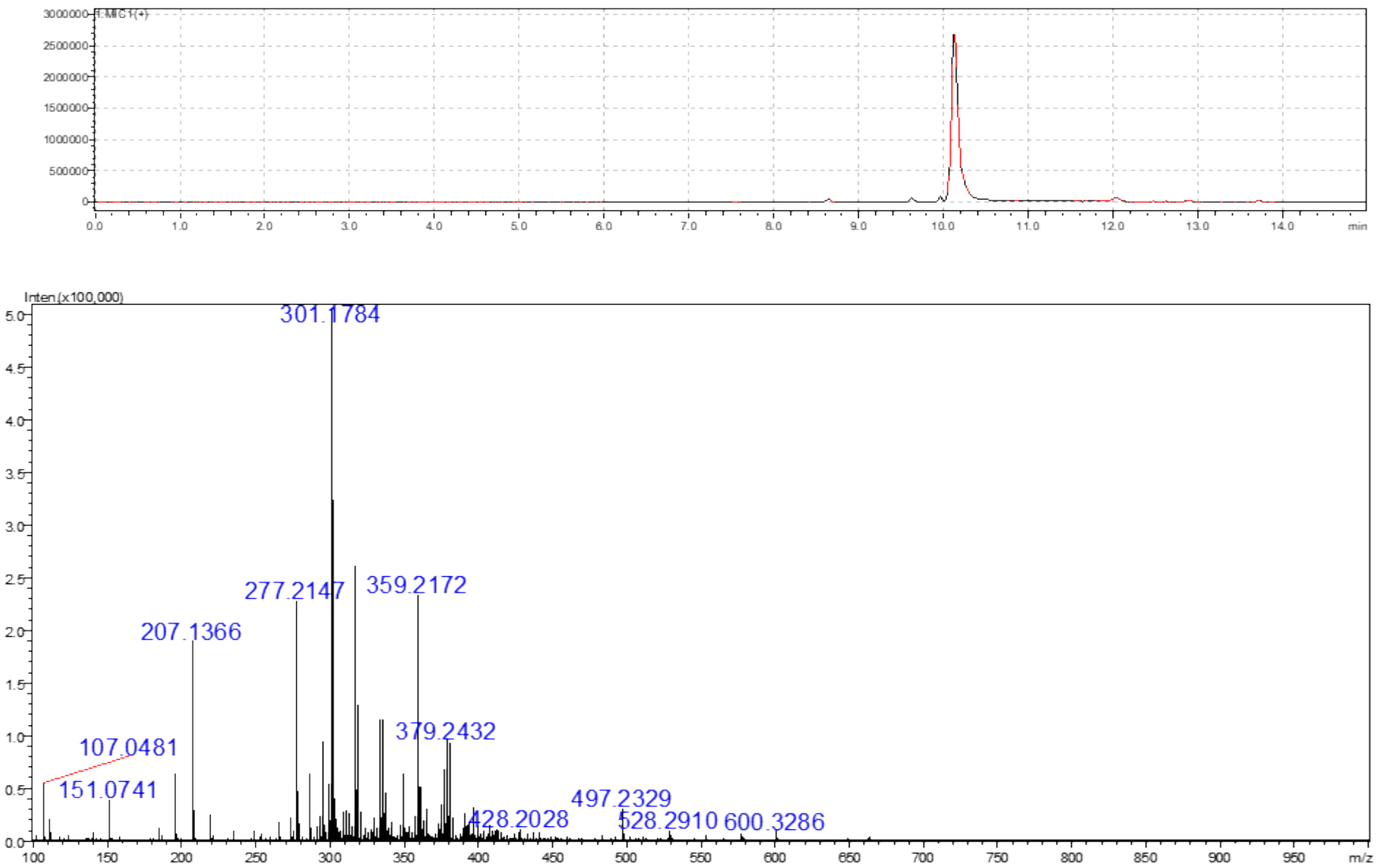

Molecular	$[M+H]^+$, m/z	Cald., m/z	Error (ppm)
$C_{19}H_{24}O_3$	301.1784	301.1798	4.7

Fig. S11. The HR-ESIMS (positive mode) spectrum of compound 2

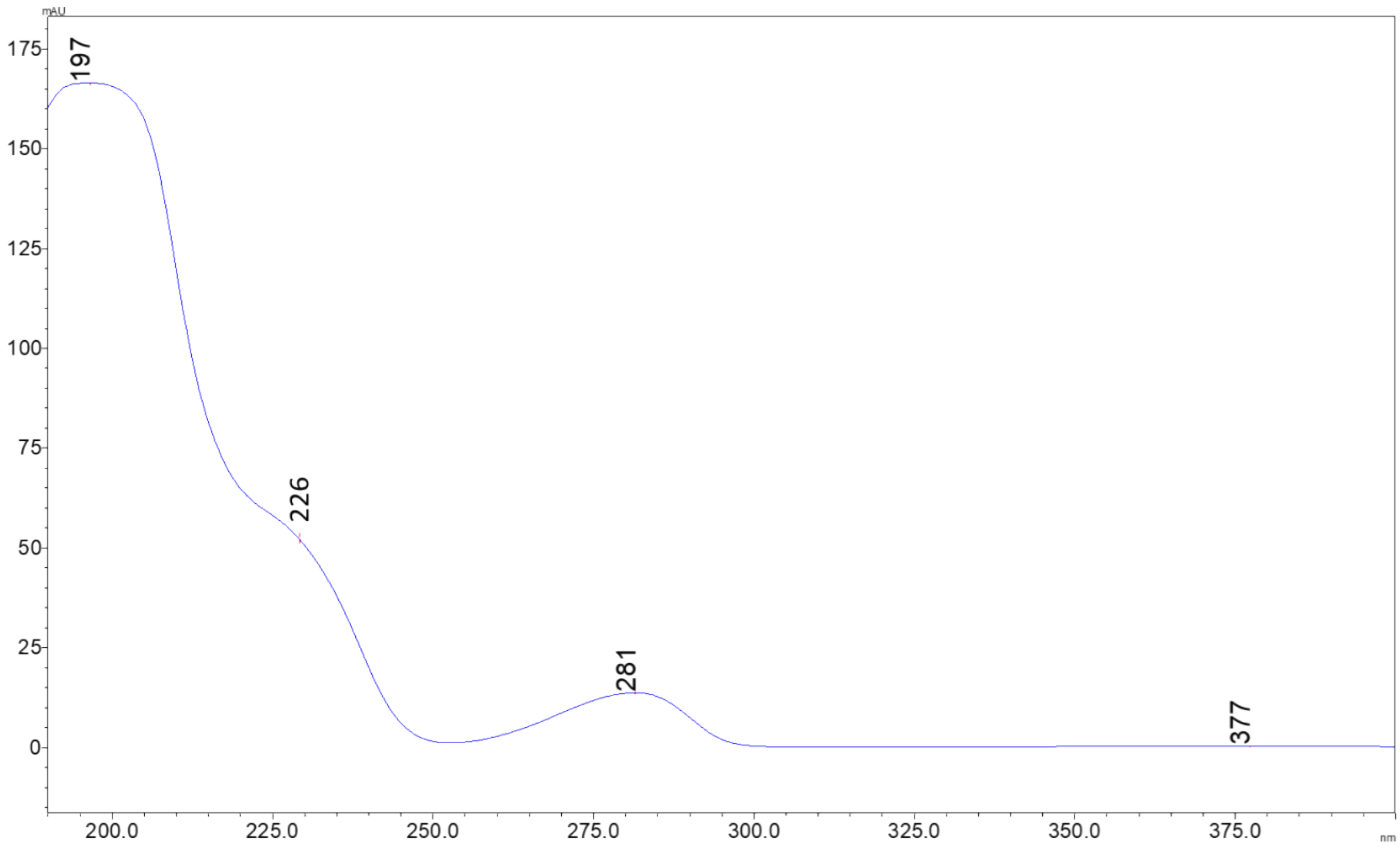

Fig. S12. The UV spectrum of compound **2**

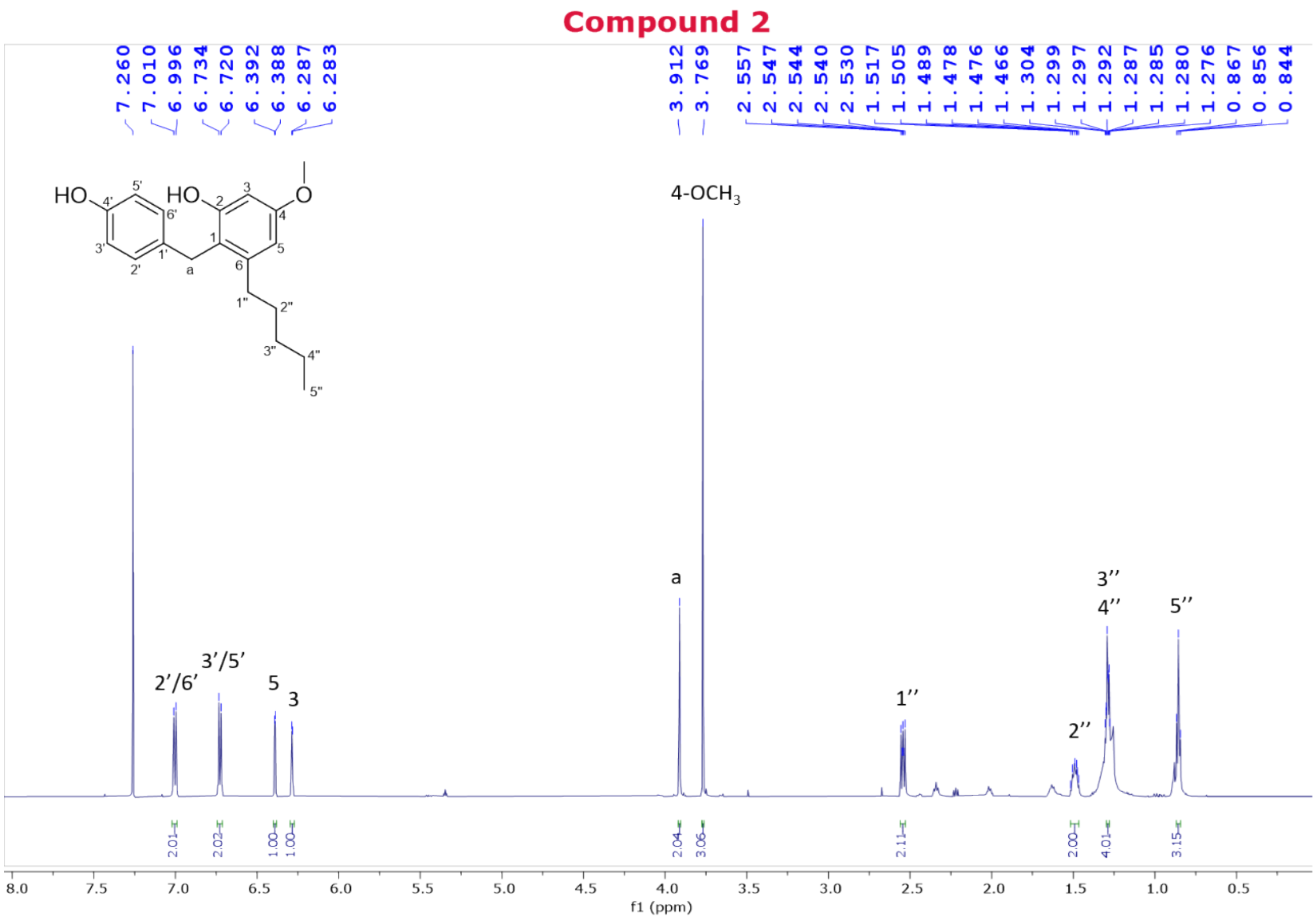

Fig. S13. The ^{1}H NMR spectrum of compound **2**

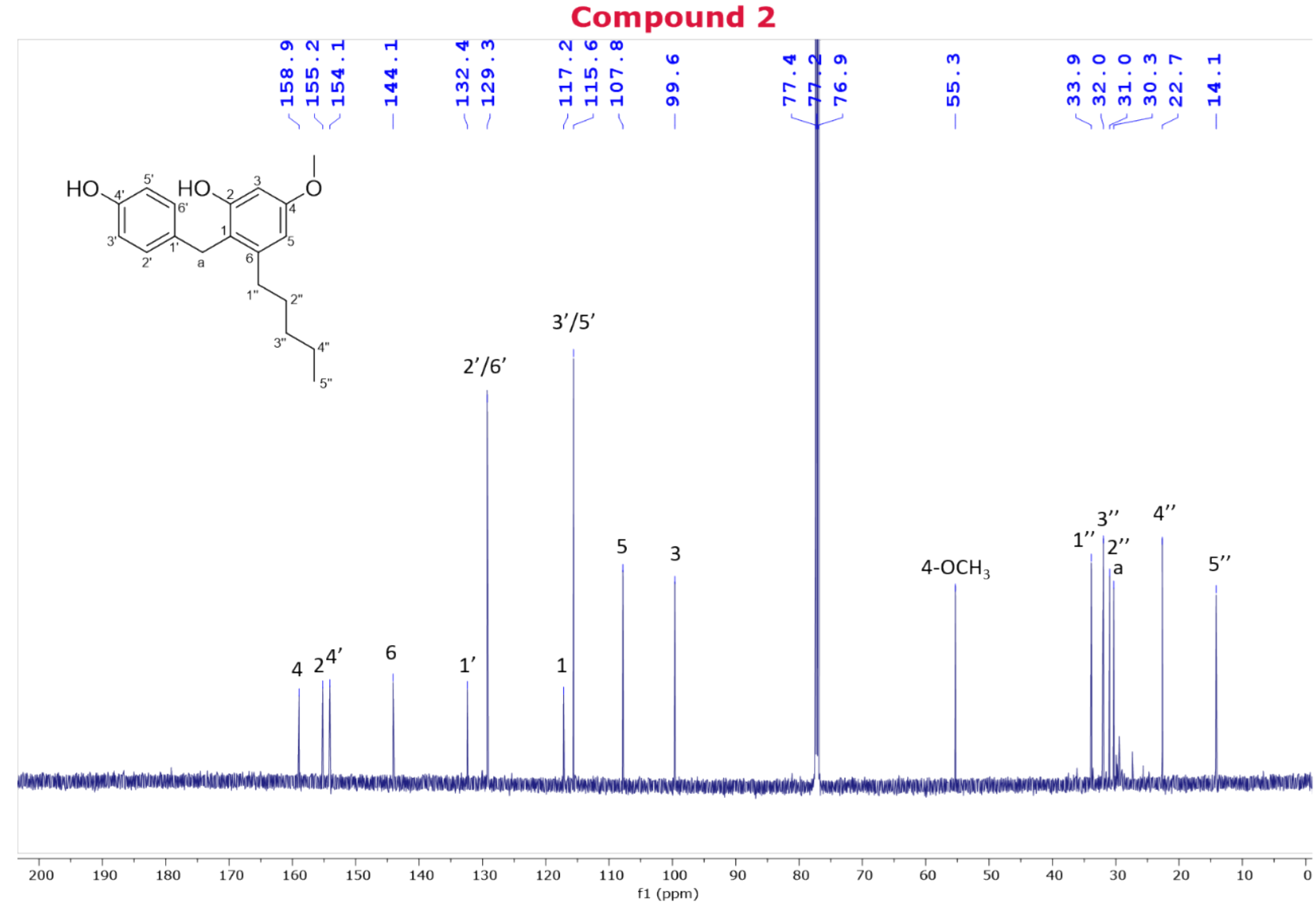

Fig. S14. The ^{13}C NMR spectrum of compound **2**

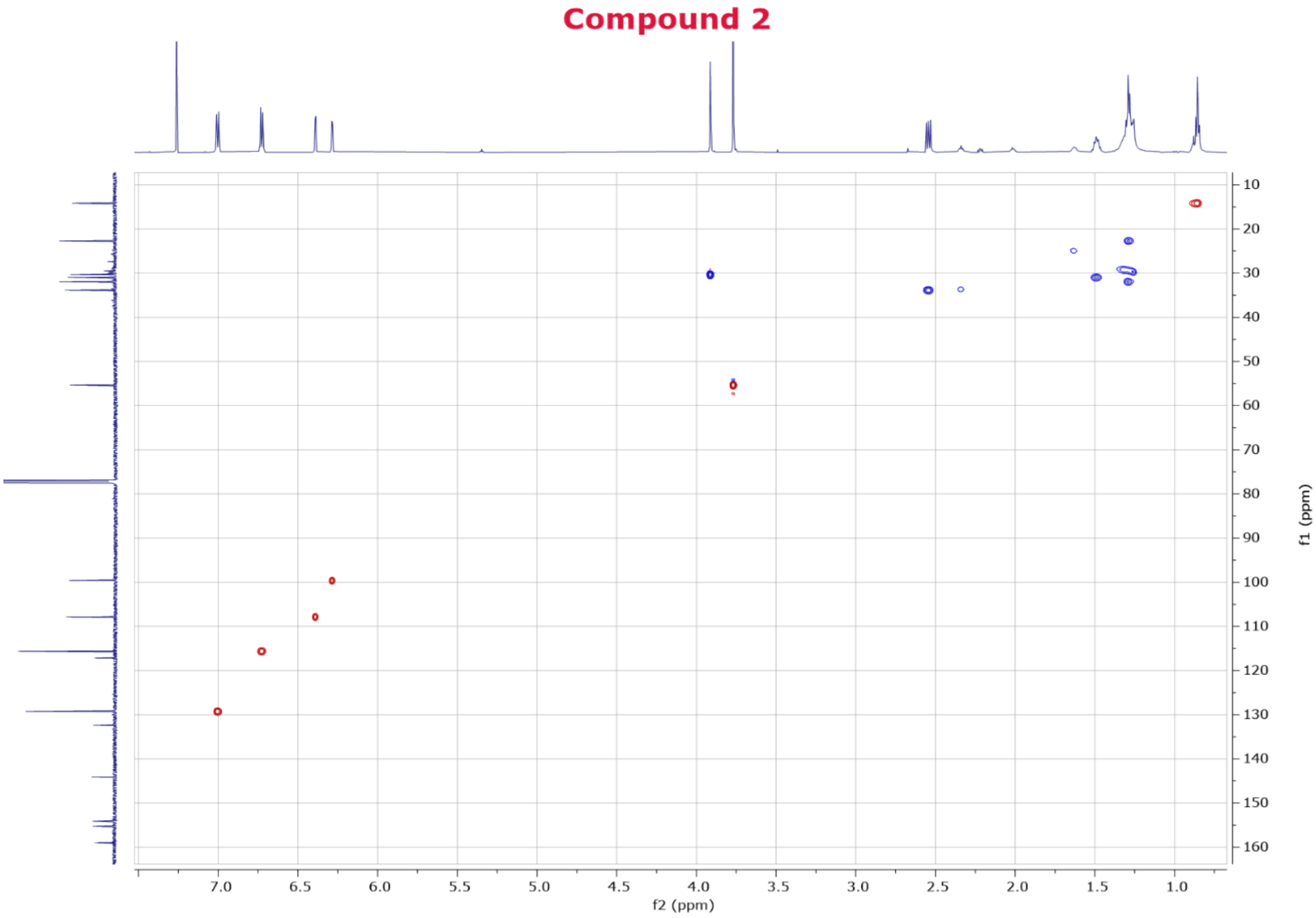

Fig. S15. The HSQC spectrum of compound **2**

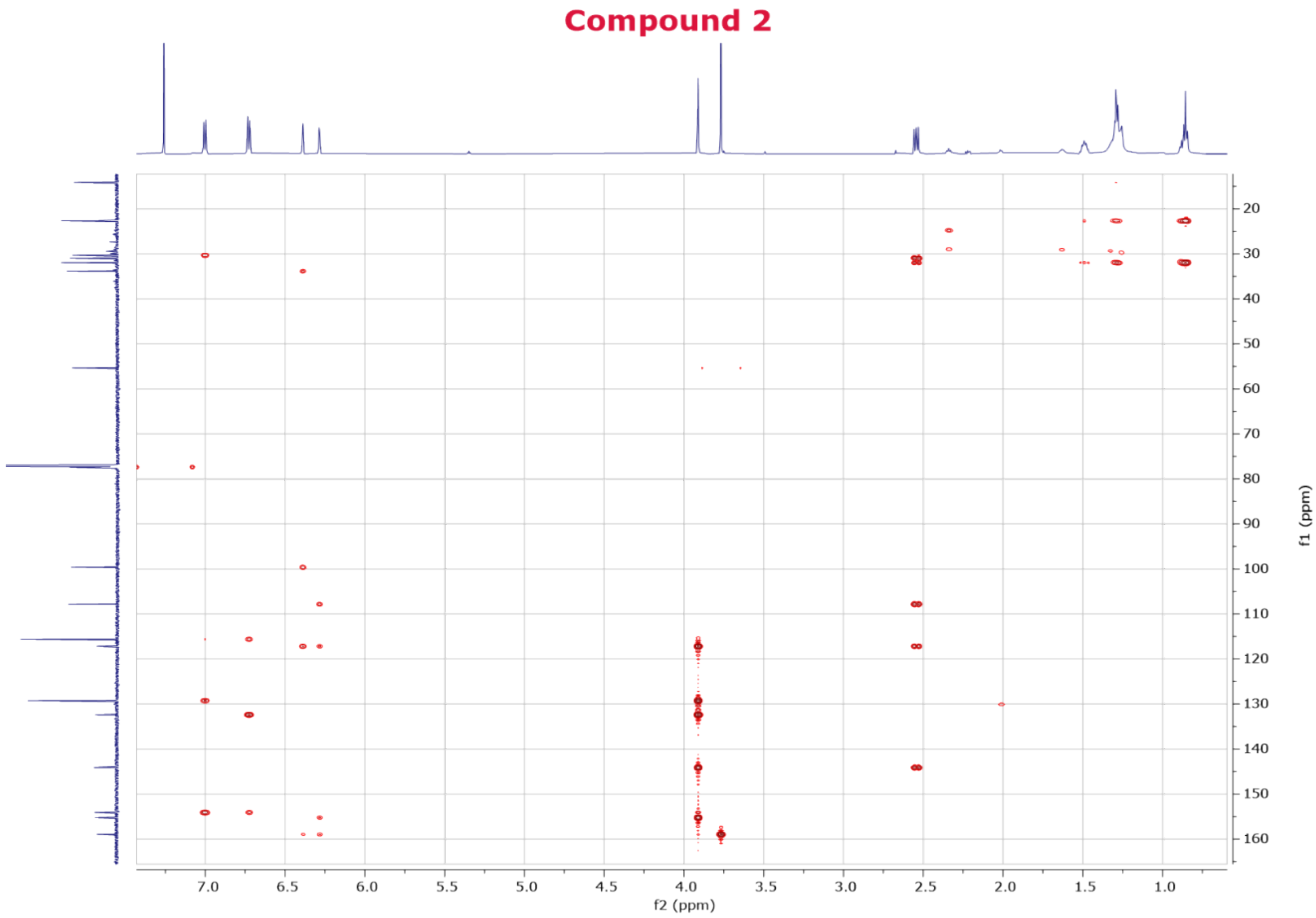

Fig. S16. The HMBC spectrum of compound **2**

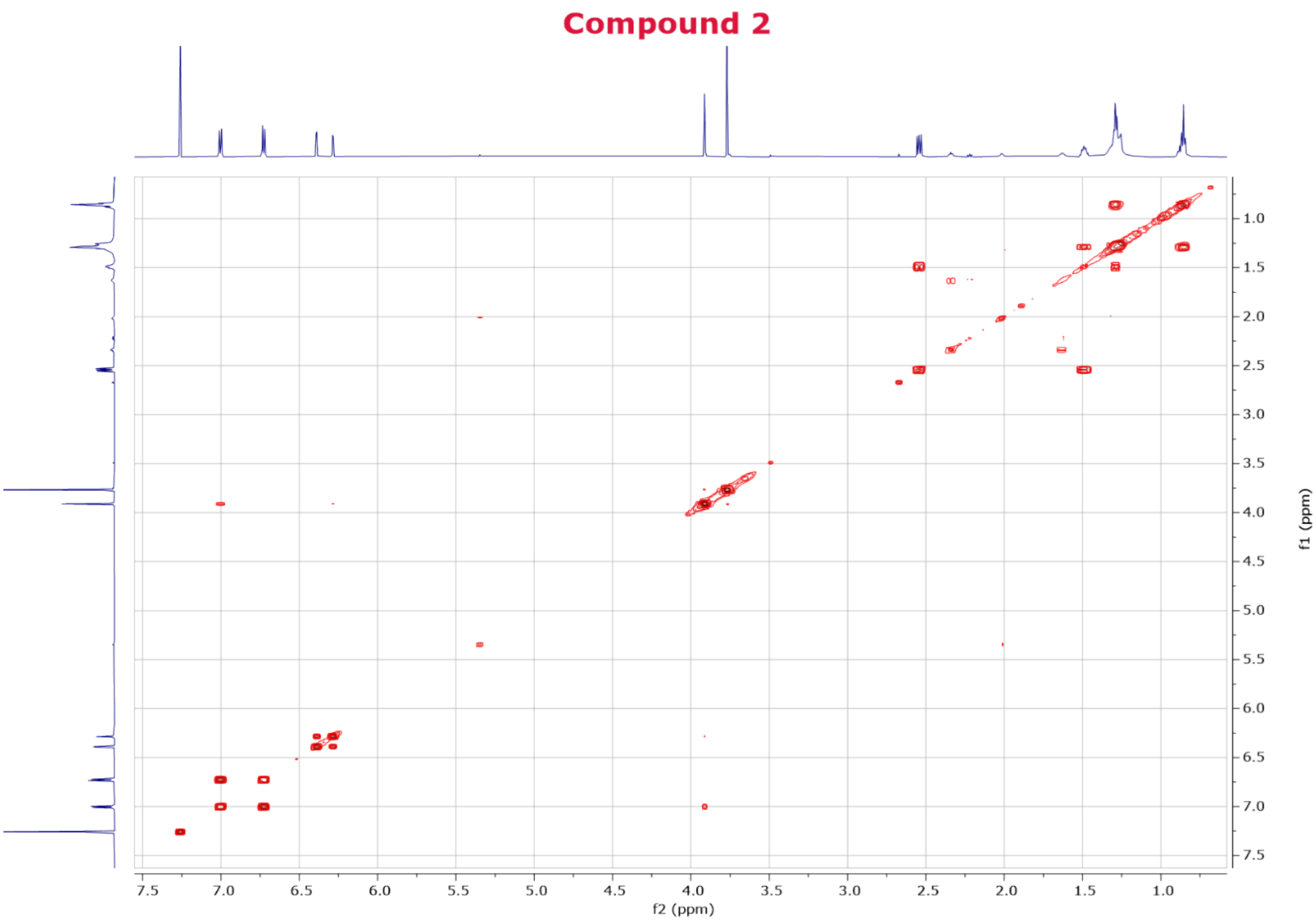

Fig. S17. The COSY spectrum of compound **2**

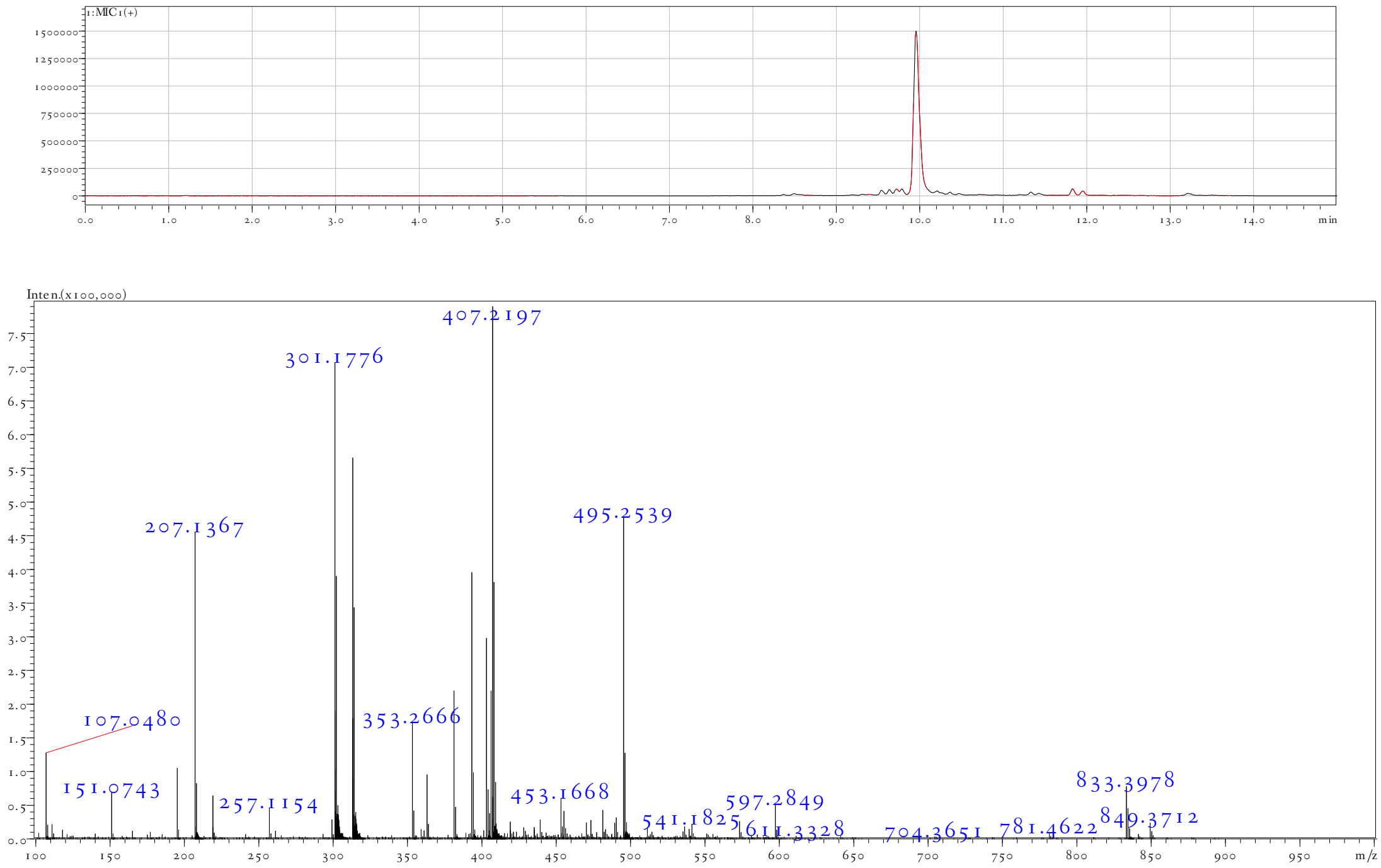

Molecular	[M+H]+, m/z	Cald., m/z	Error (ppm)
$C_{26}H_{30}O_4$	407.2197	407.2217	4.9

Fig. S18. The HR-ESIMS (positive mode) spectrum of compound **3**

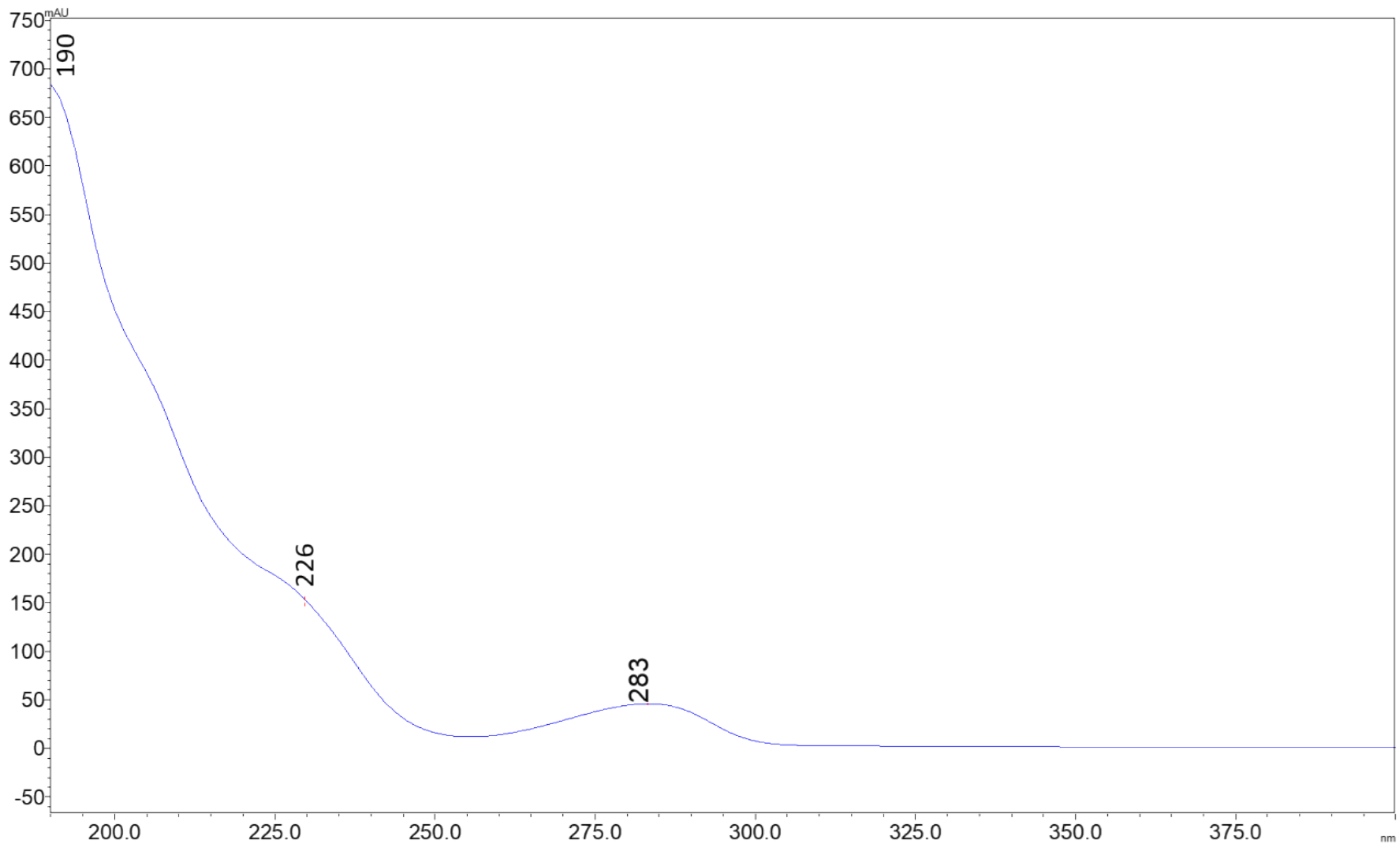

Fig. S19. The UV spectrum of compound **3**

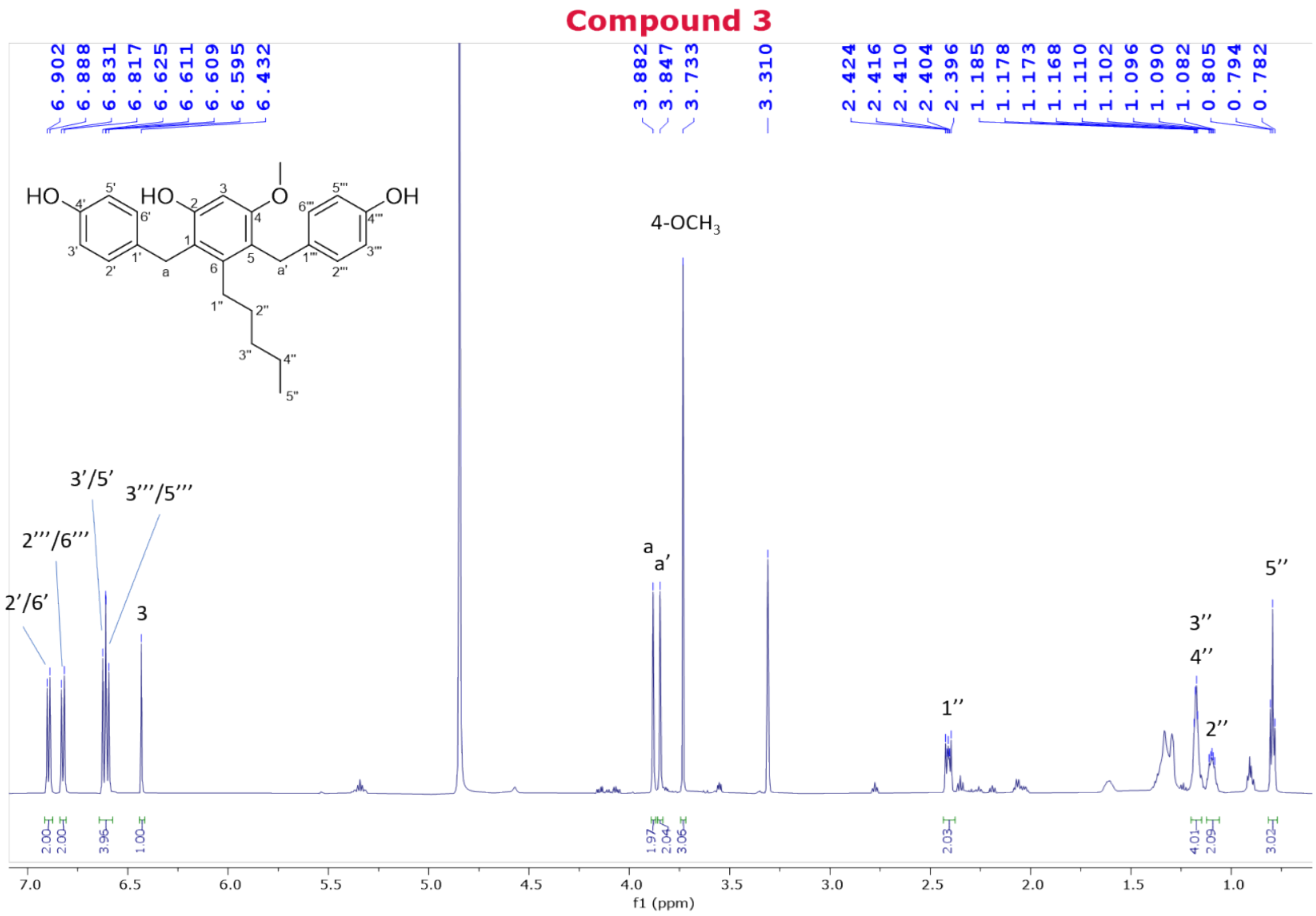

Fig. S20. The ¹H NMR spectrum of compound **3**

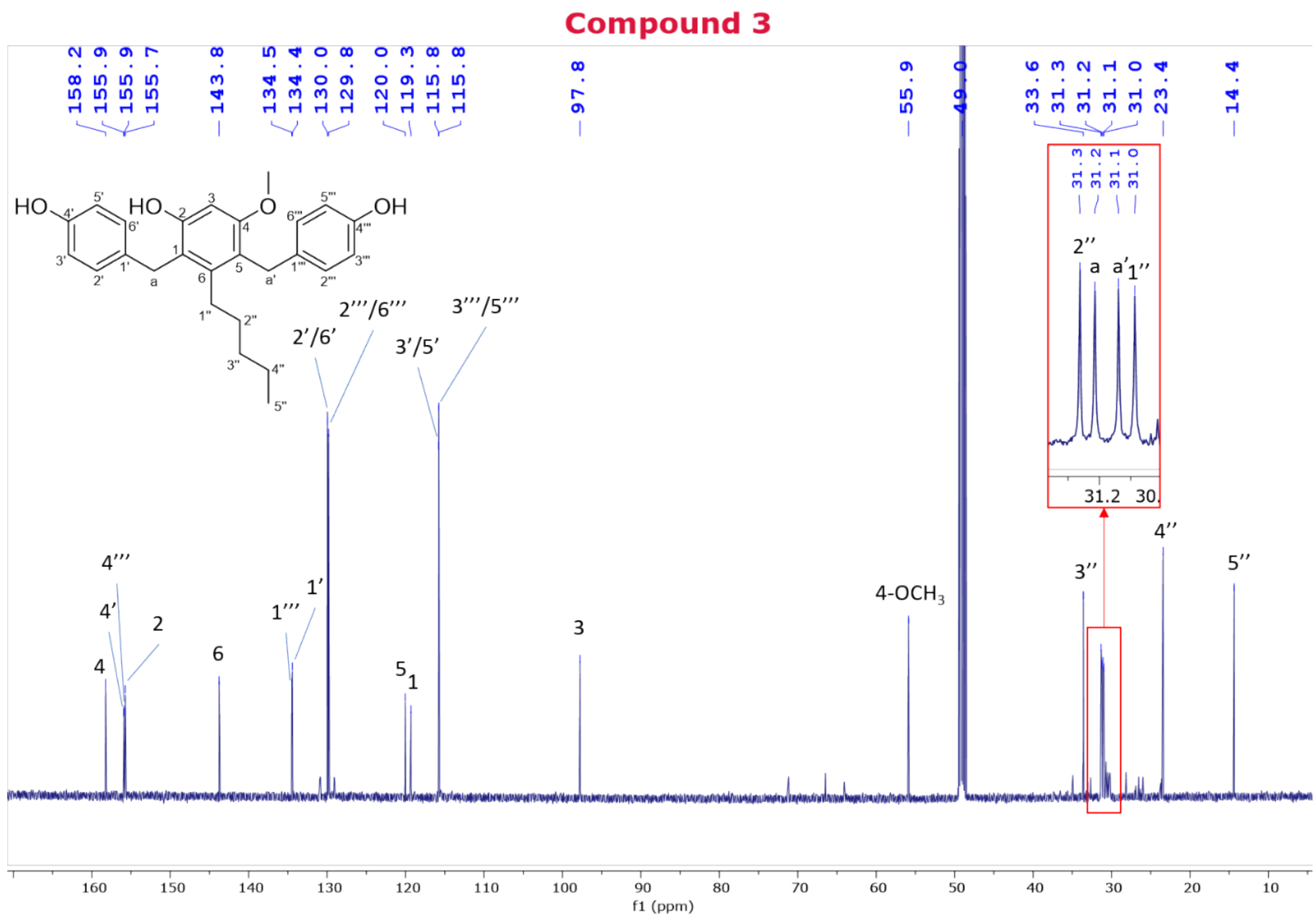

Fig. S21. The ¹³C NMR spectrum of compound **3**

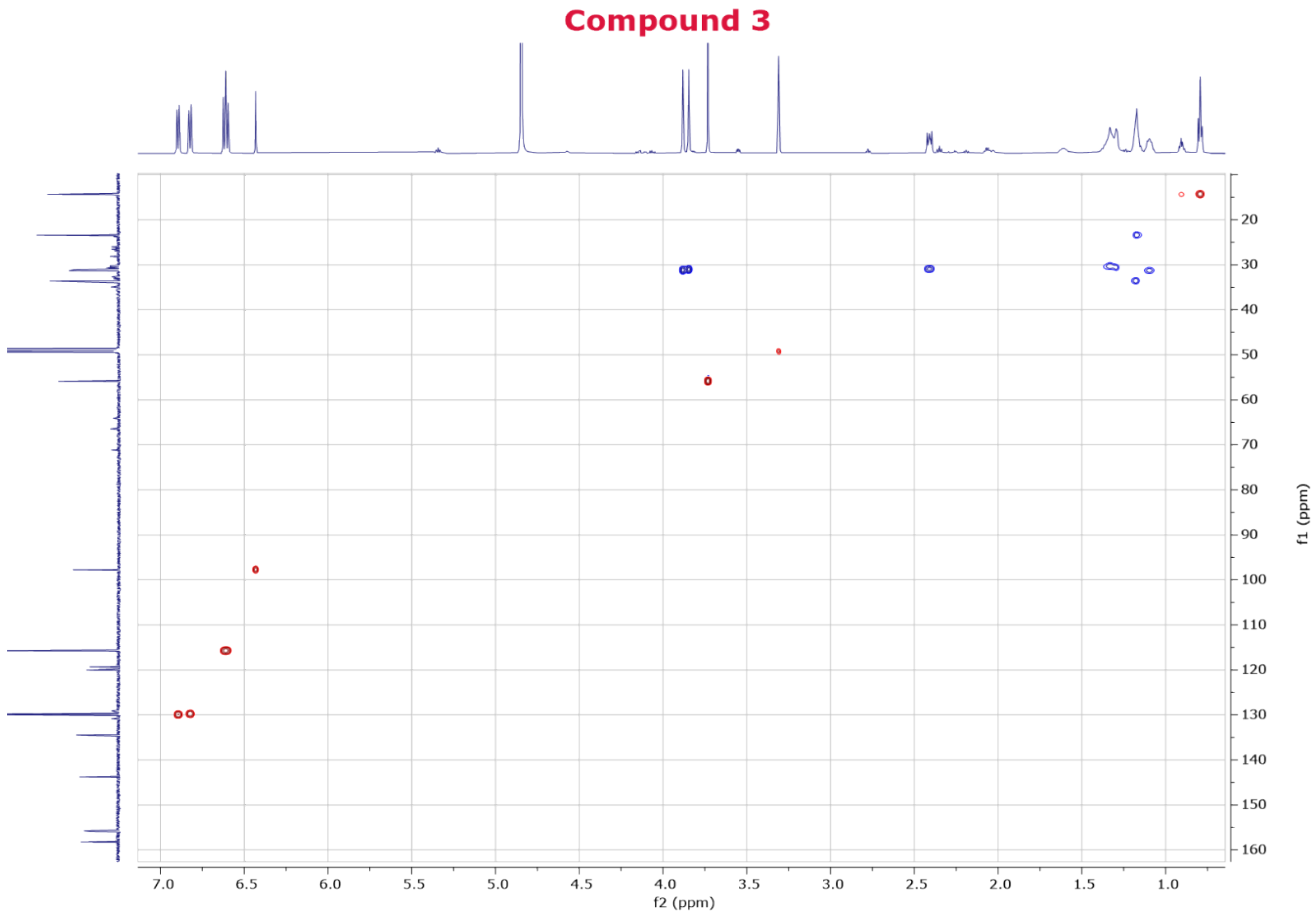

Fig. S22. The HSQC spectrum of compound **3**

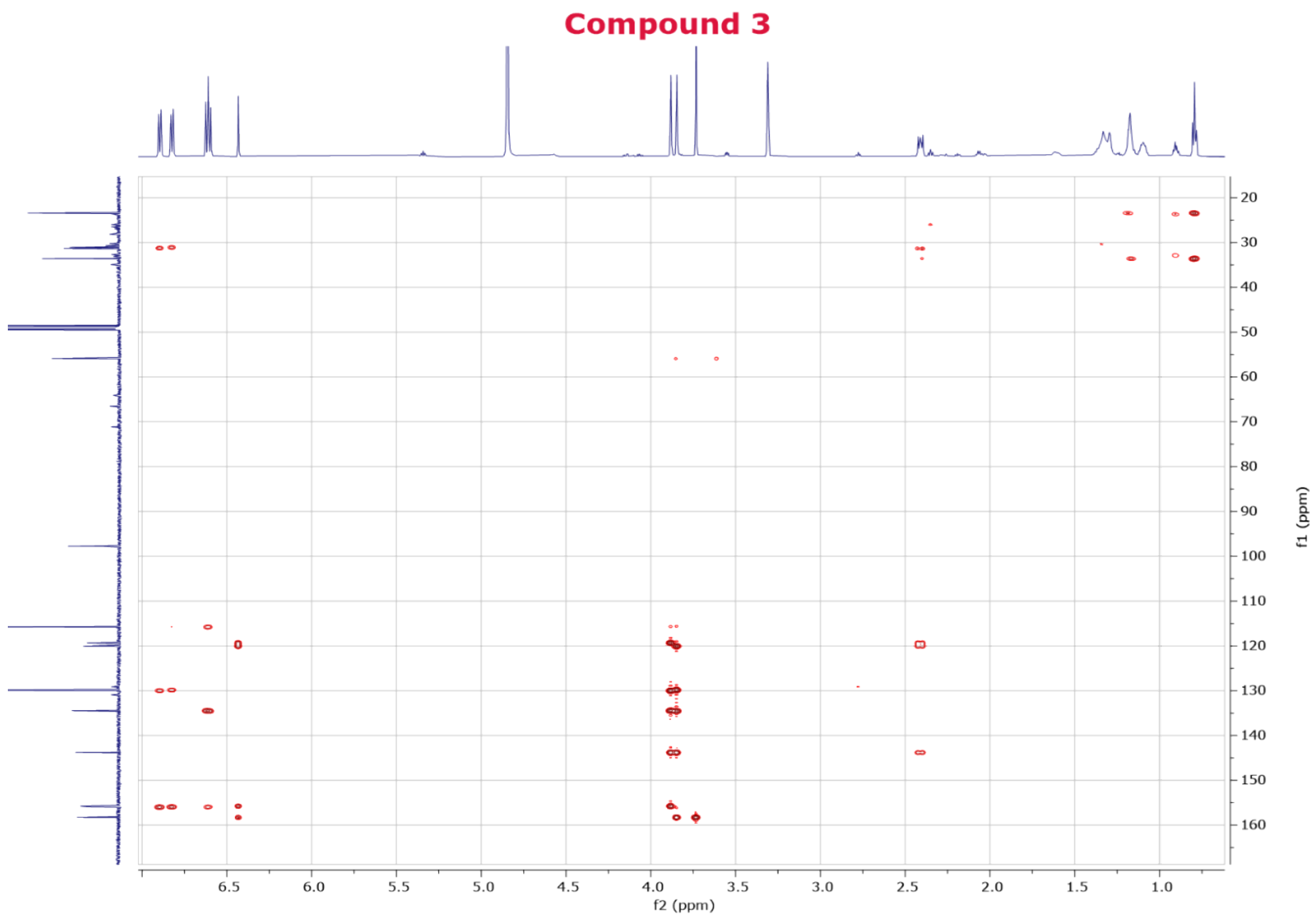

Fig. S23. The HMBC spectrum of compound **3**

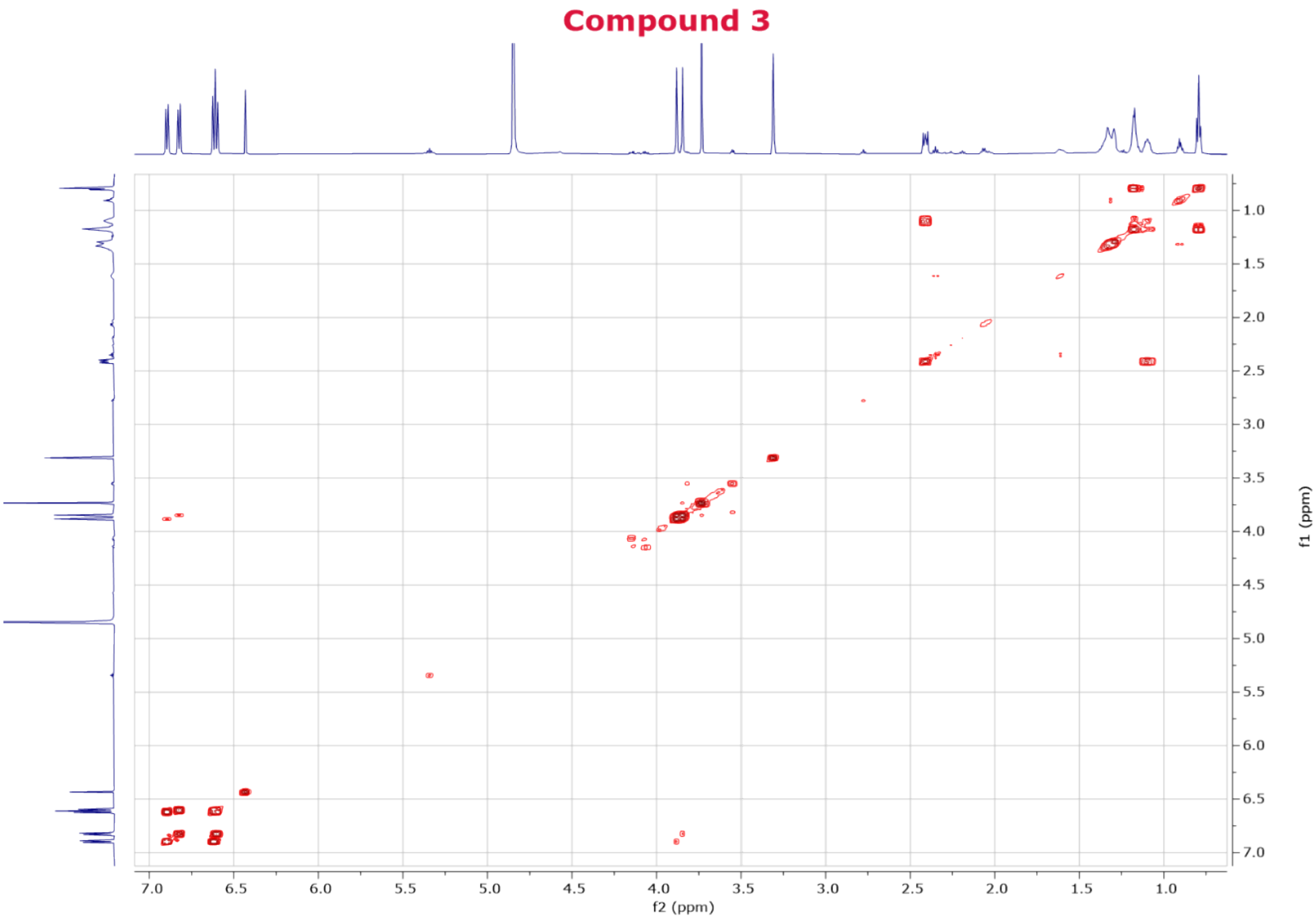

Fig. S24. The COSY spectrum of compound 3

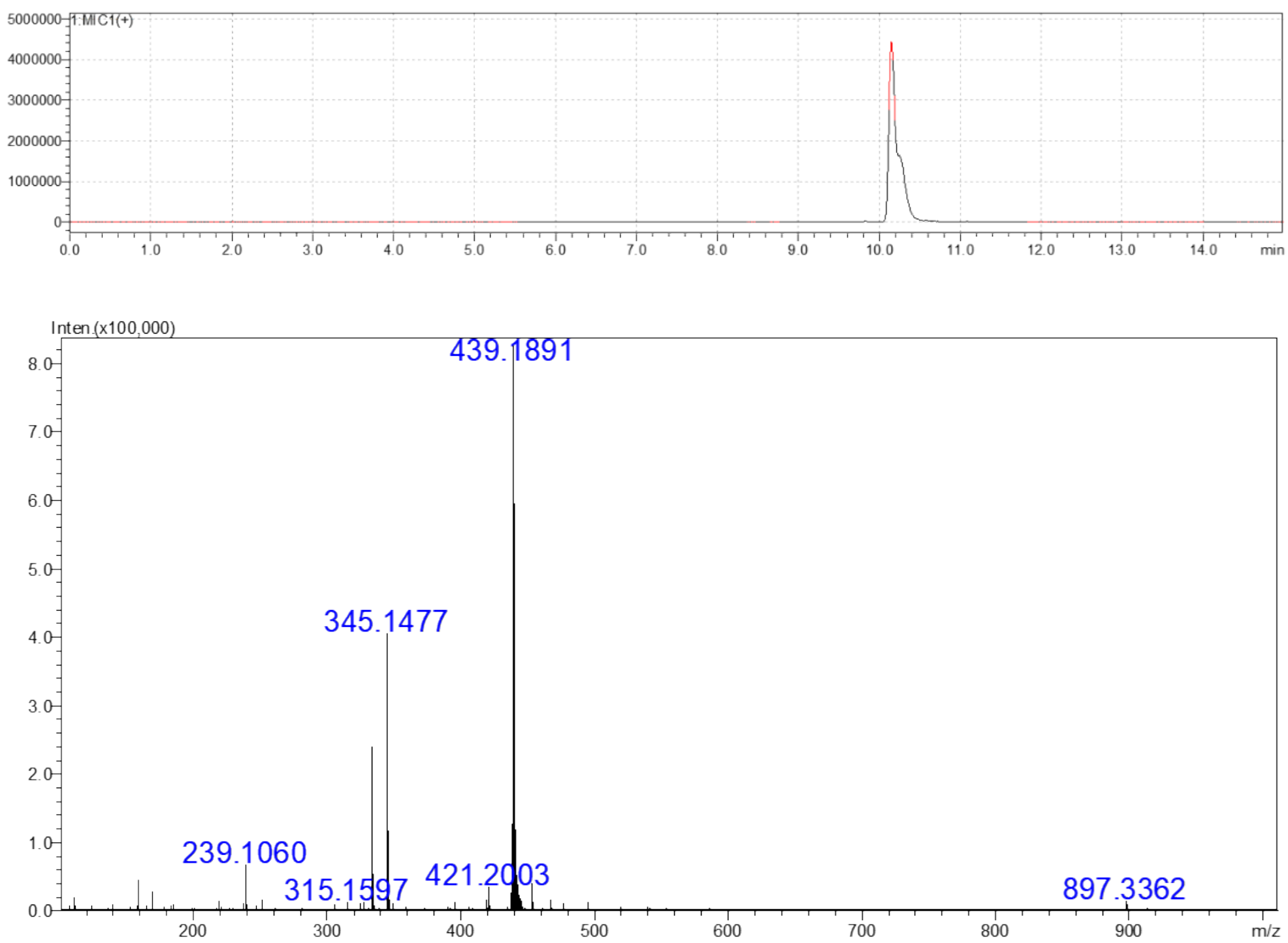

Molecular	[M+H]+, m/z	Cald., m/z	Error (ppm)
$C_{29}H_{26}O_4$	439.1891	439.1904	2.9

Fig. S25. The HR-ESIMS (positive mode) spectrum of compound **4**

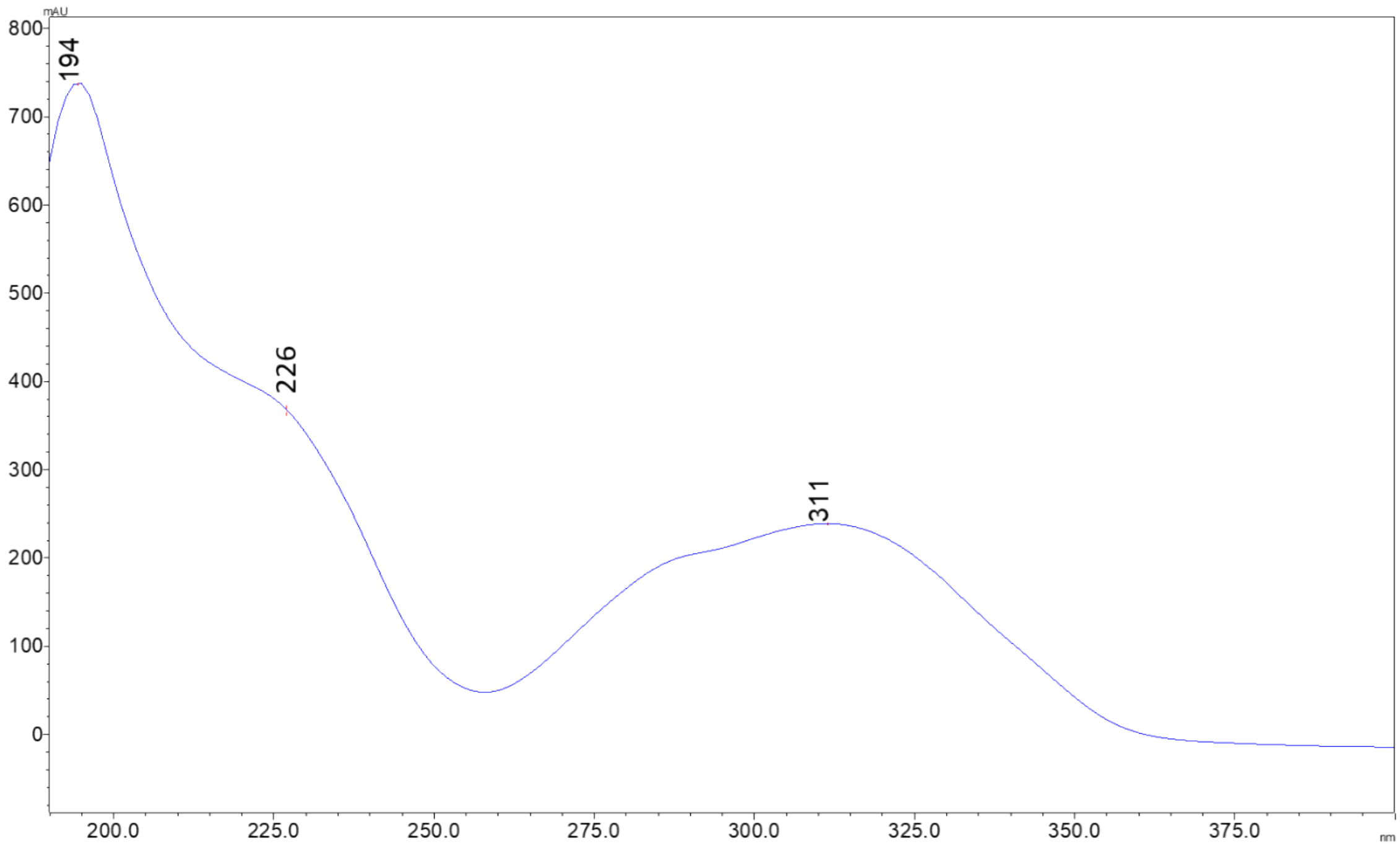

Fig. S26. The UV spectrum of compound **4**

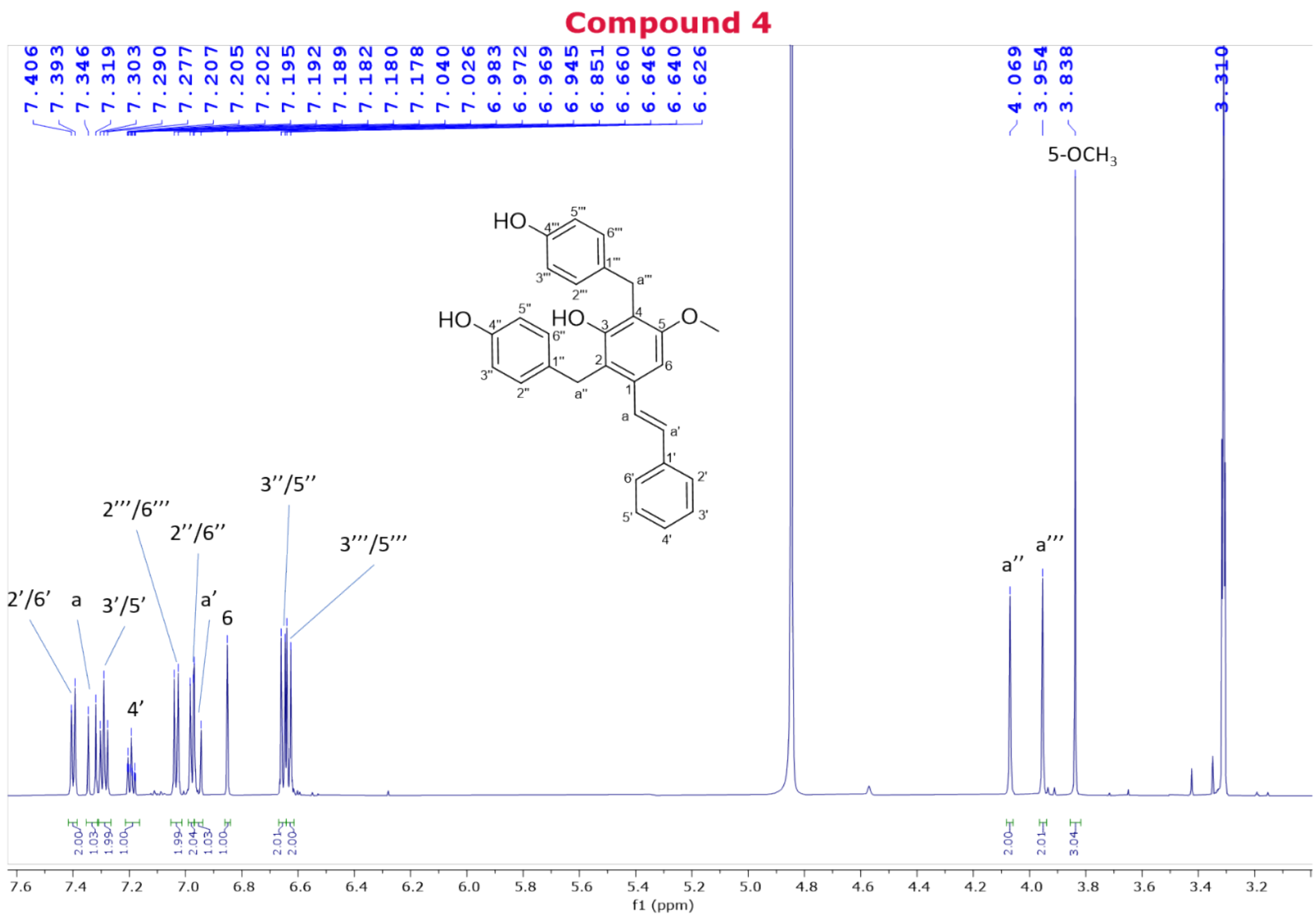

Fig. S27. The ^{1}H NMR spectrum of compound **4**

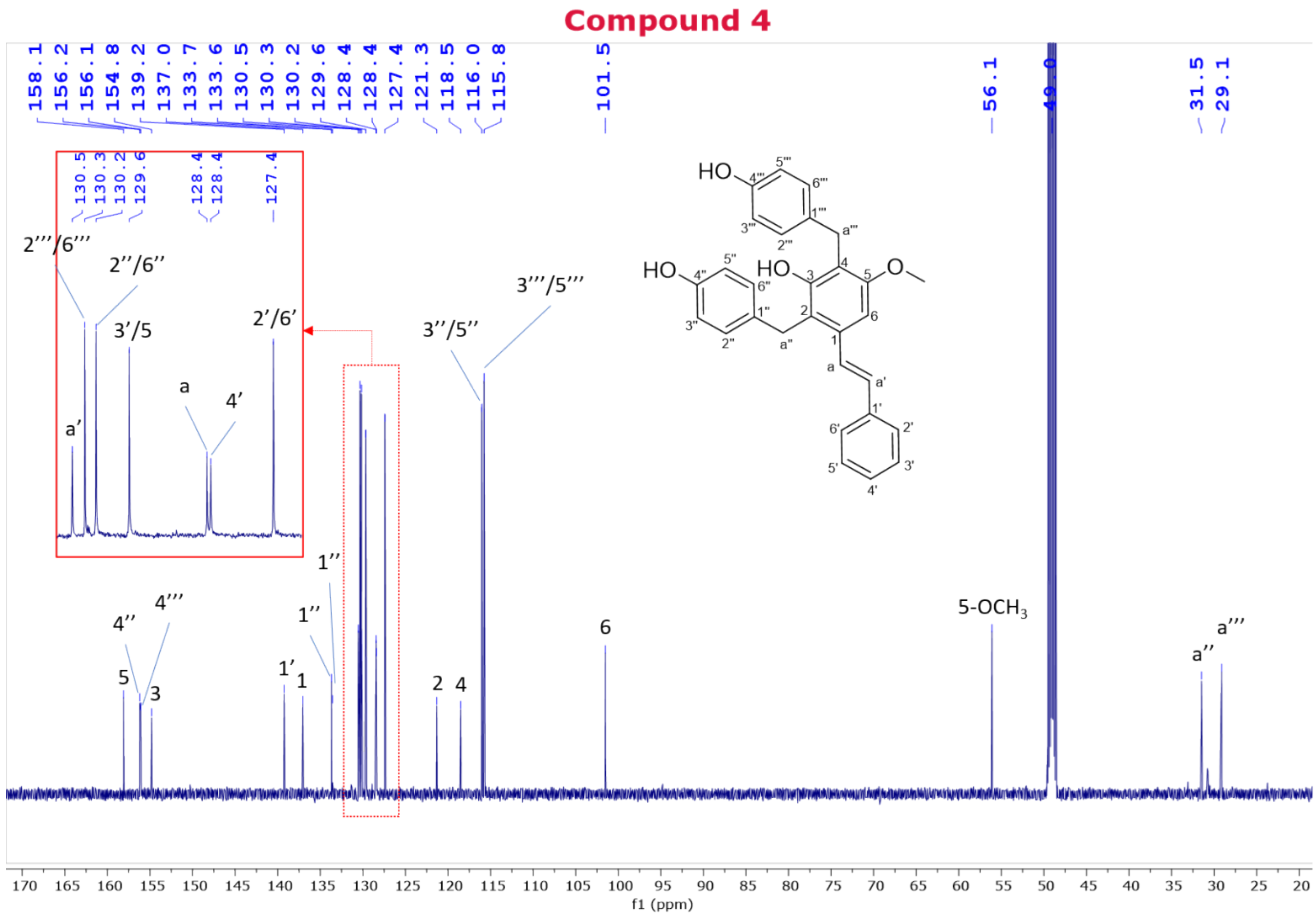

Fig. S28. The ^{13}C NMR spectrum of compound **4**

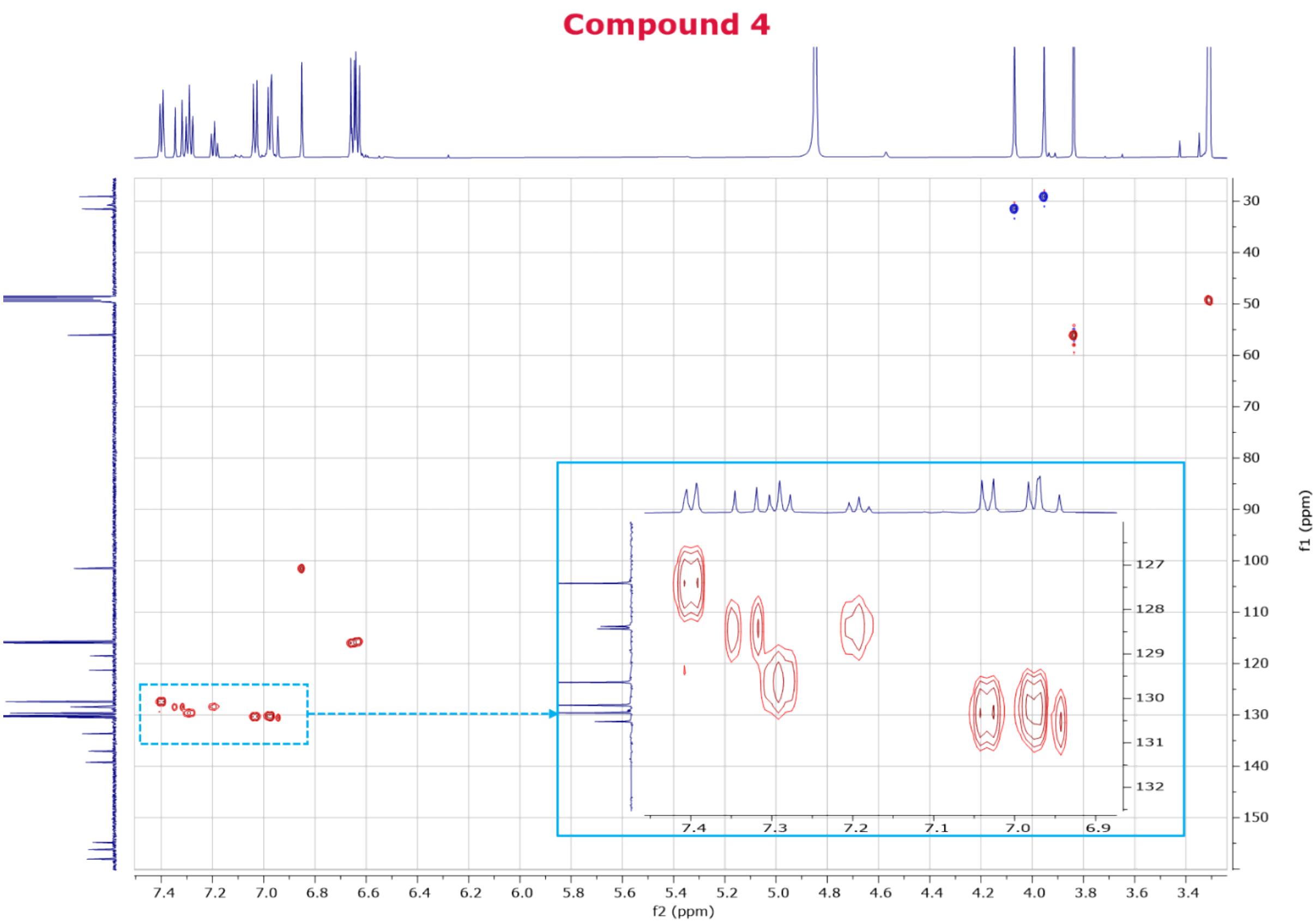

Fig. S29. The HSQC spectrum of compound **4**

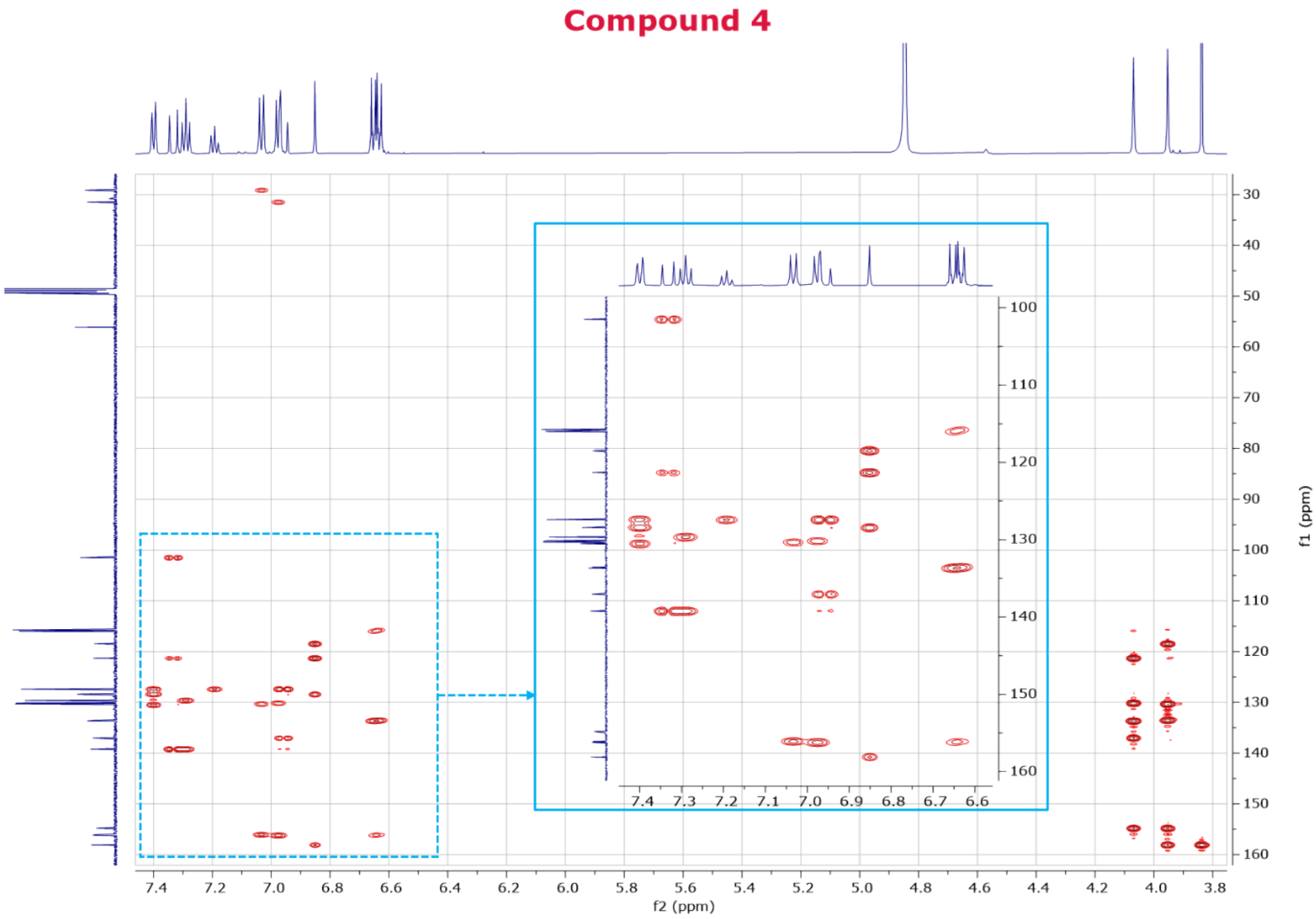

Fig. S30. The HMBC spectrum of compound **4**

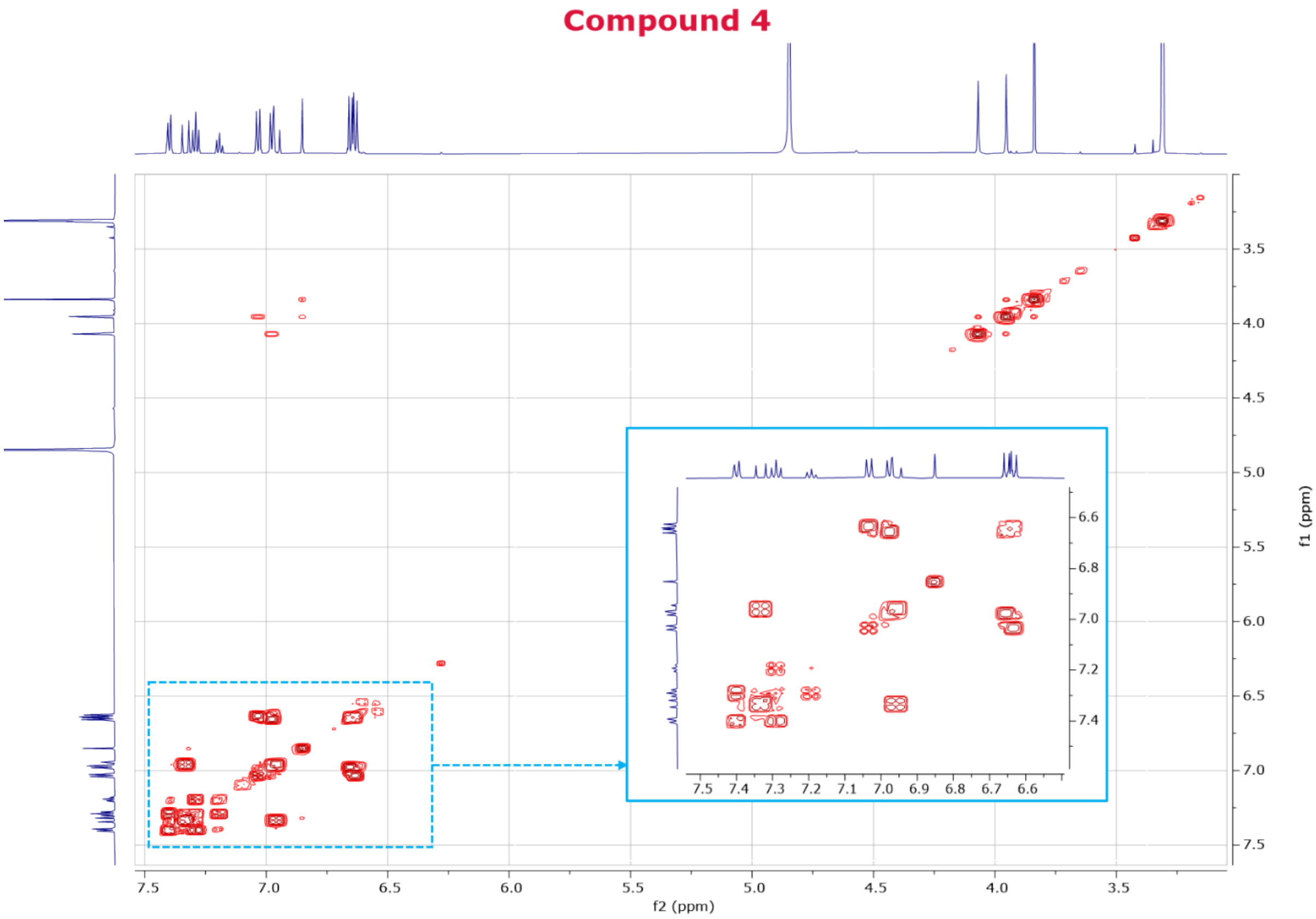

Fig. S31. The COSY spectrum of compound **4**

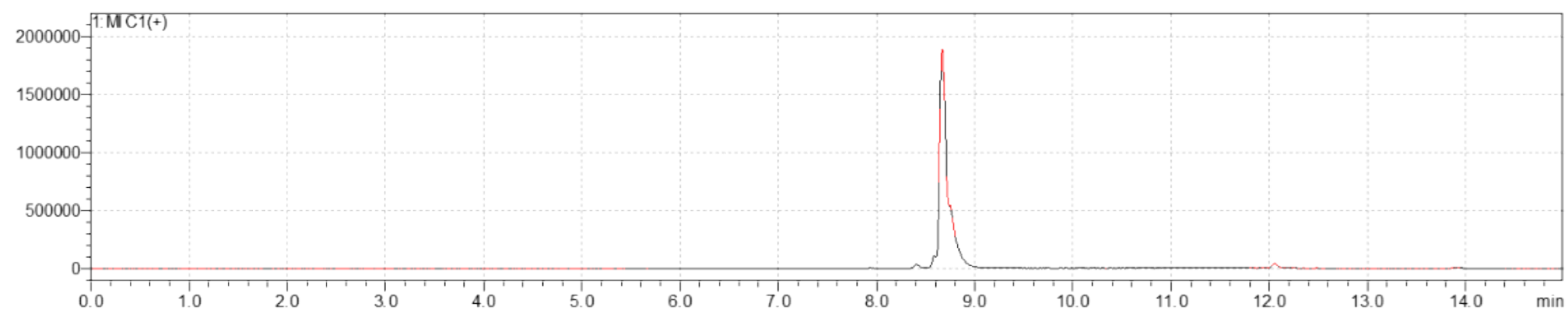

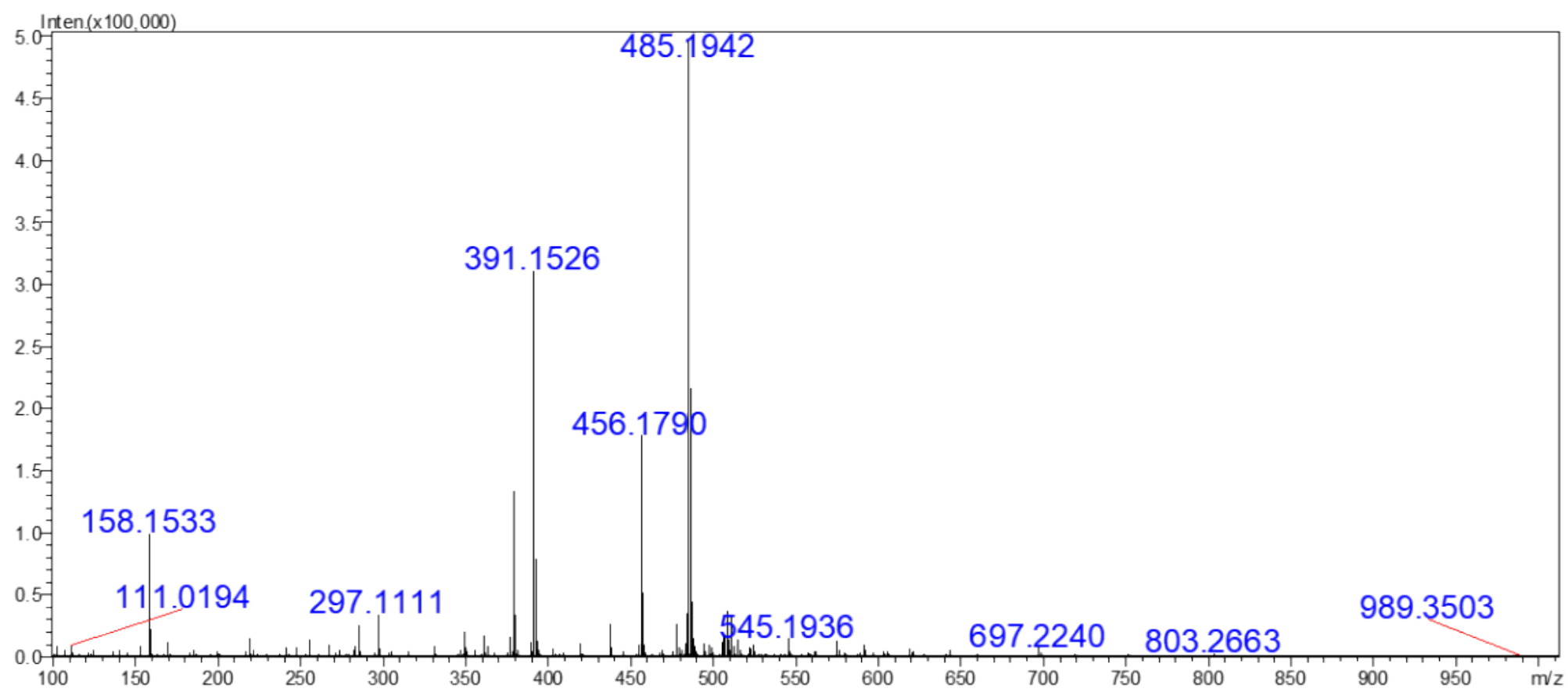

Molecular	[M+H]+, m/z	Cald., m/z	Error (ppm)
$C_{30}H_{28}O_6$	485.1942	485.1959	3.4

Fig. S32. The HR-ESIMS (positive mode) spectrum of compound **5**

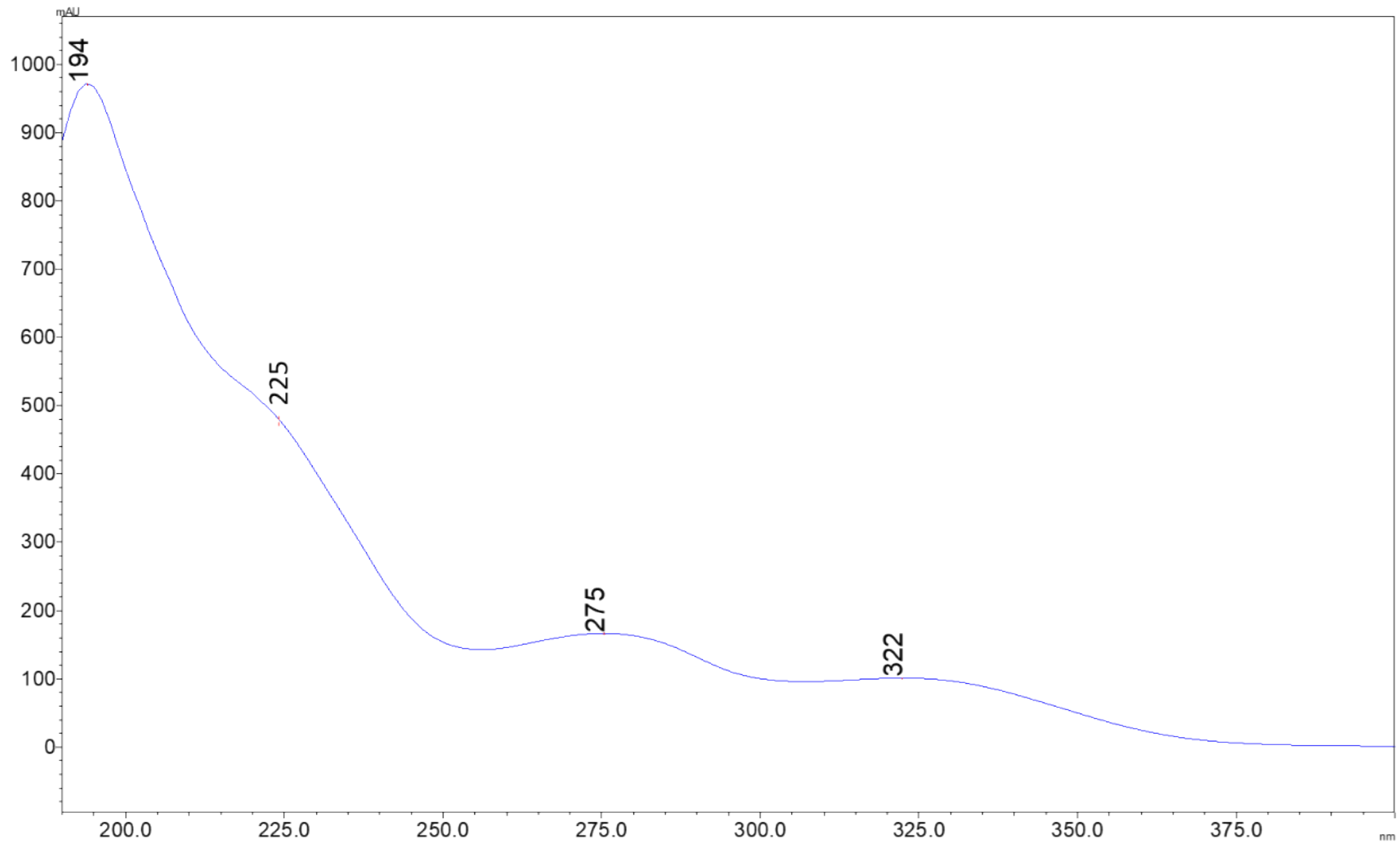

Fig. S33. The UV spectrum of compound **5**

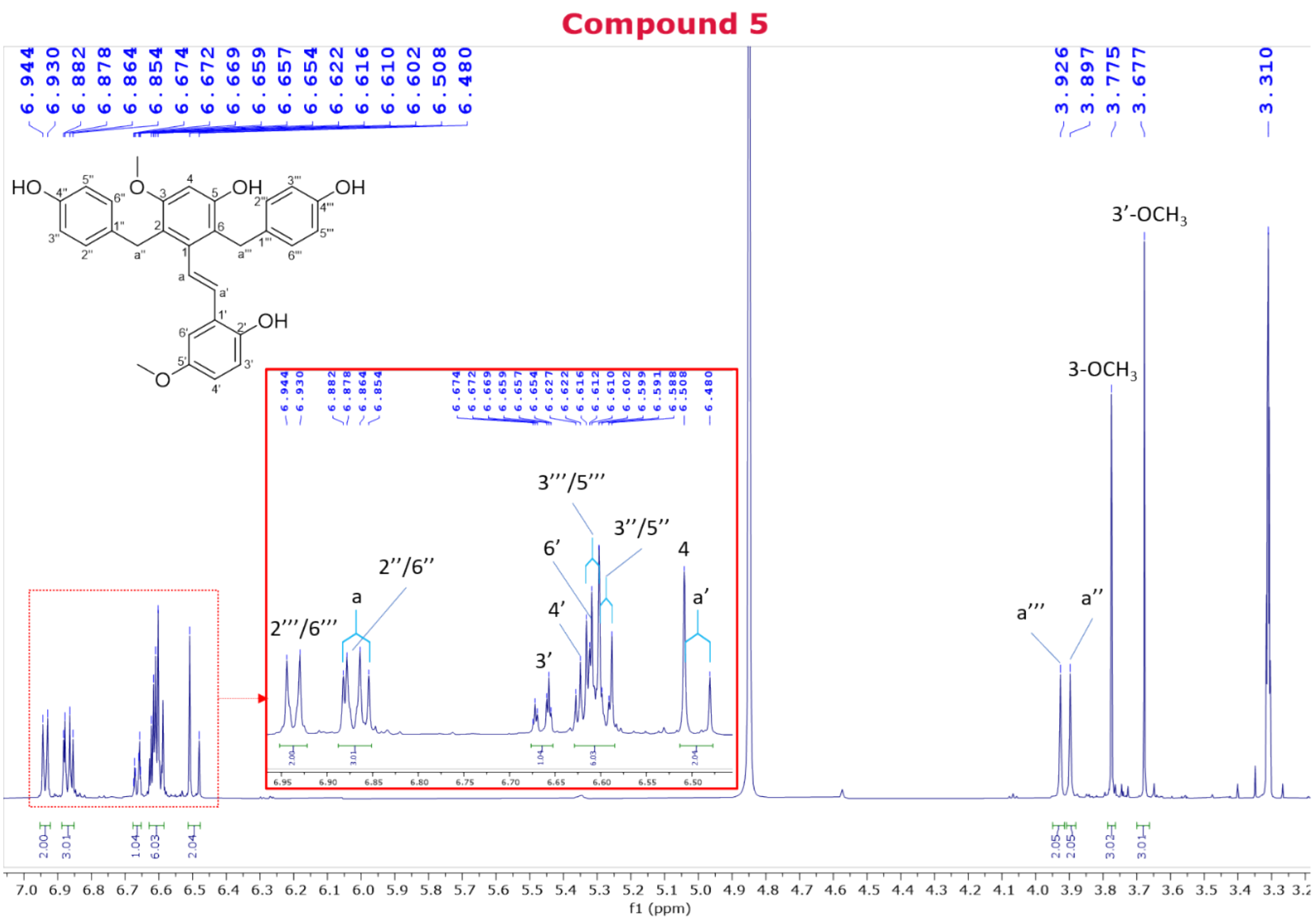

Fig. S34. The ^{1}H NMR spectrum of compound **5**

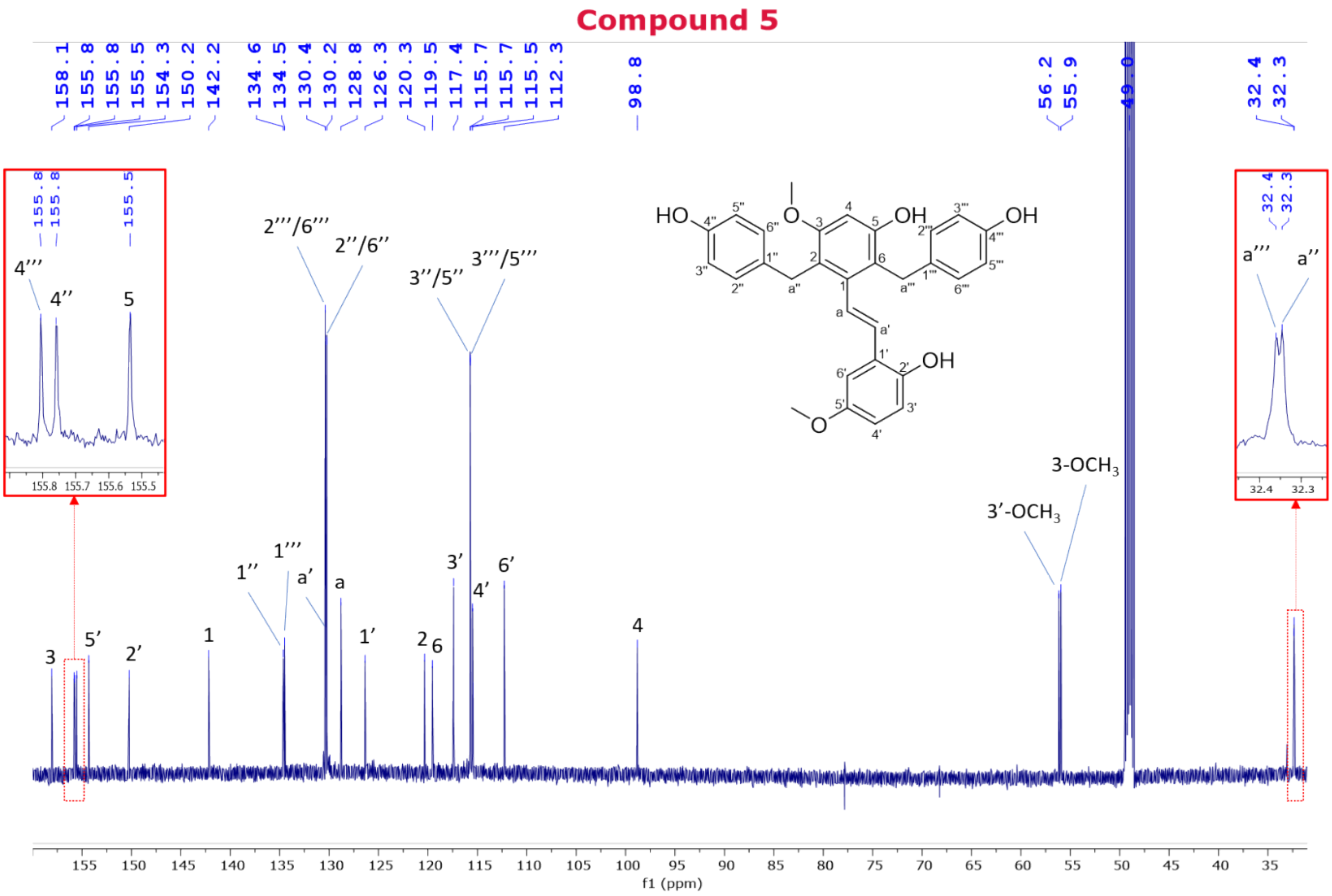

Fig. S35. The ^{13}C NMR spectrum of compound **5**

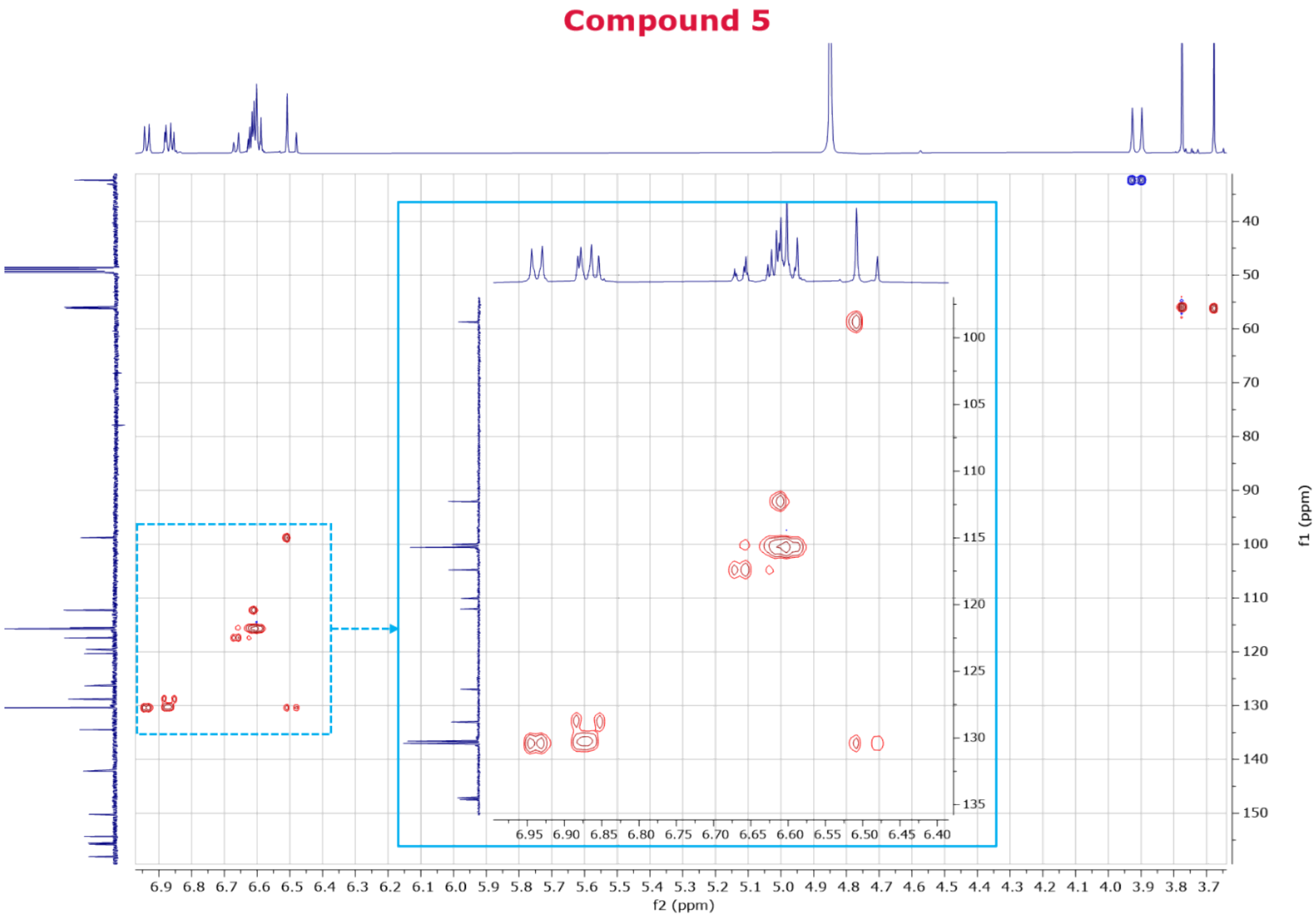

Fig. S36. The HSQC spectrum of compound **5**

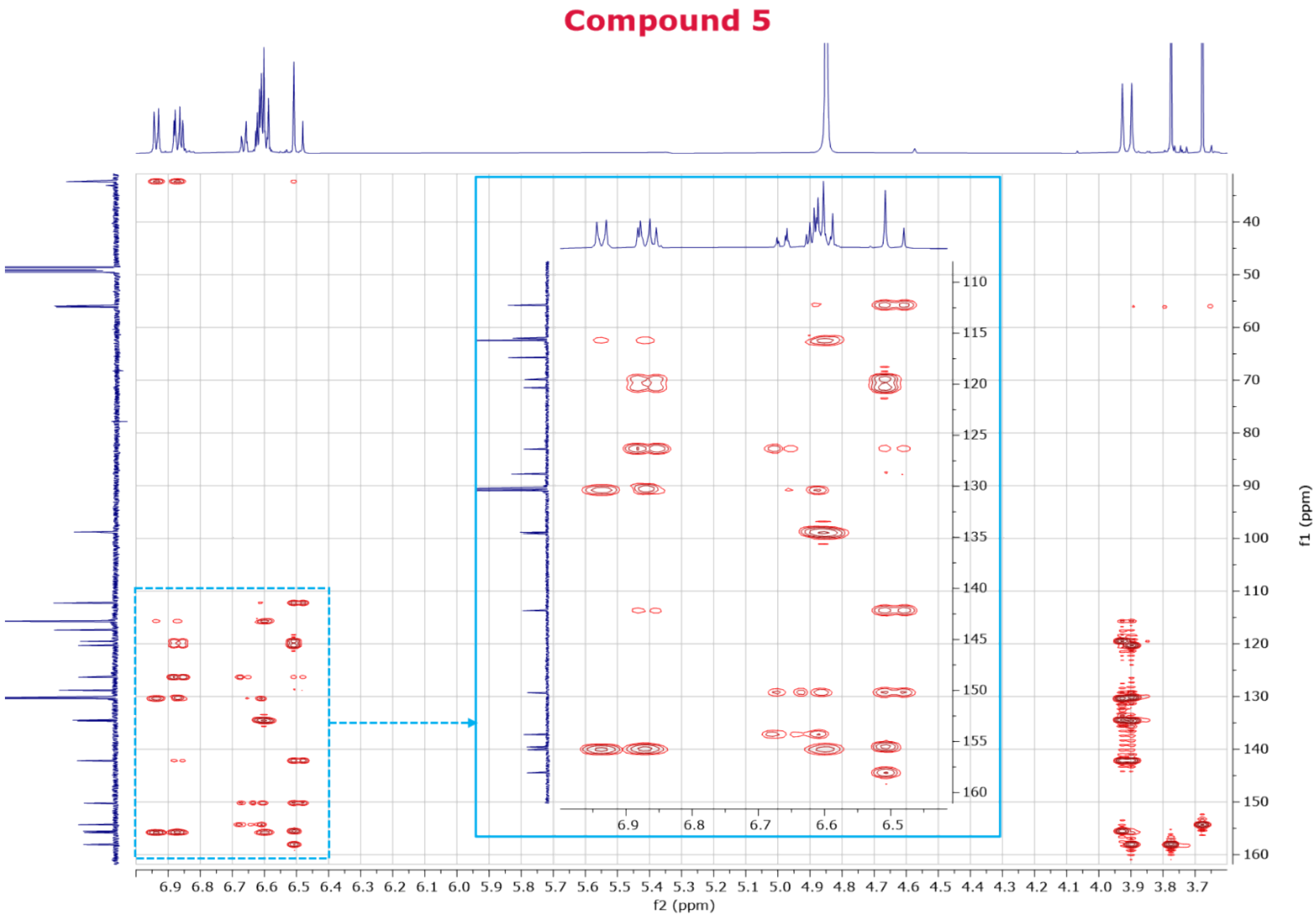

Fig. S37. The HMBC spectrum of compound **5**

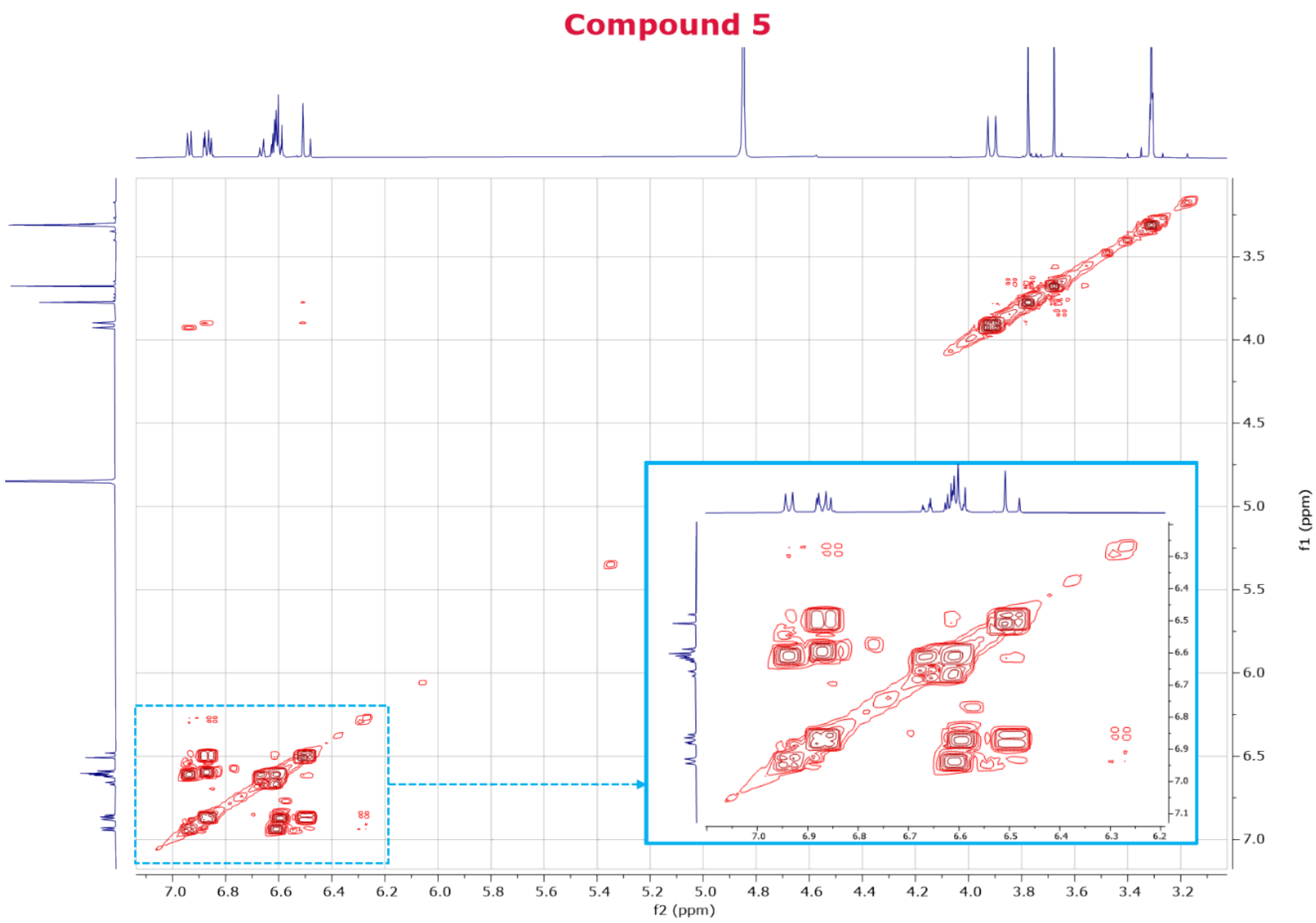

Fig. S38. The COSY spectrum of compound **5**

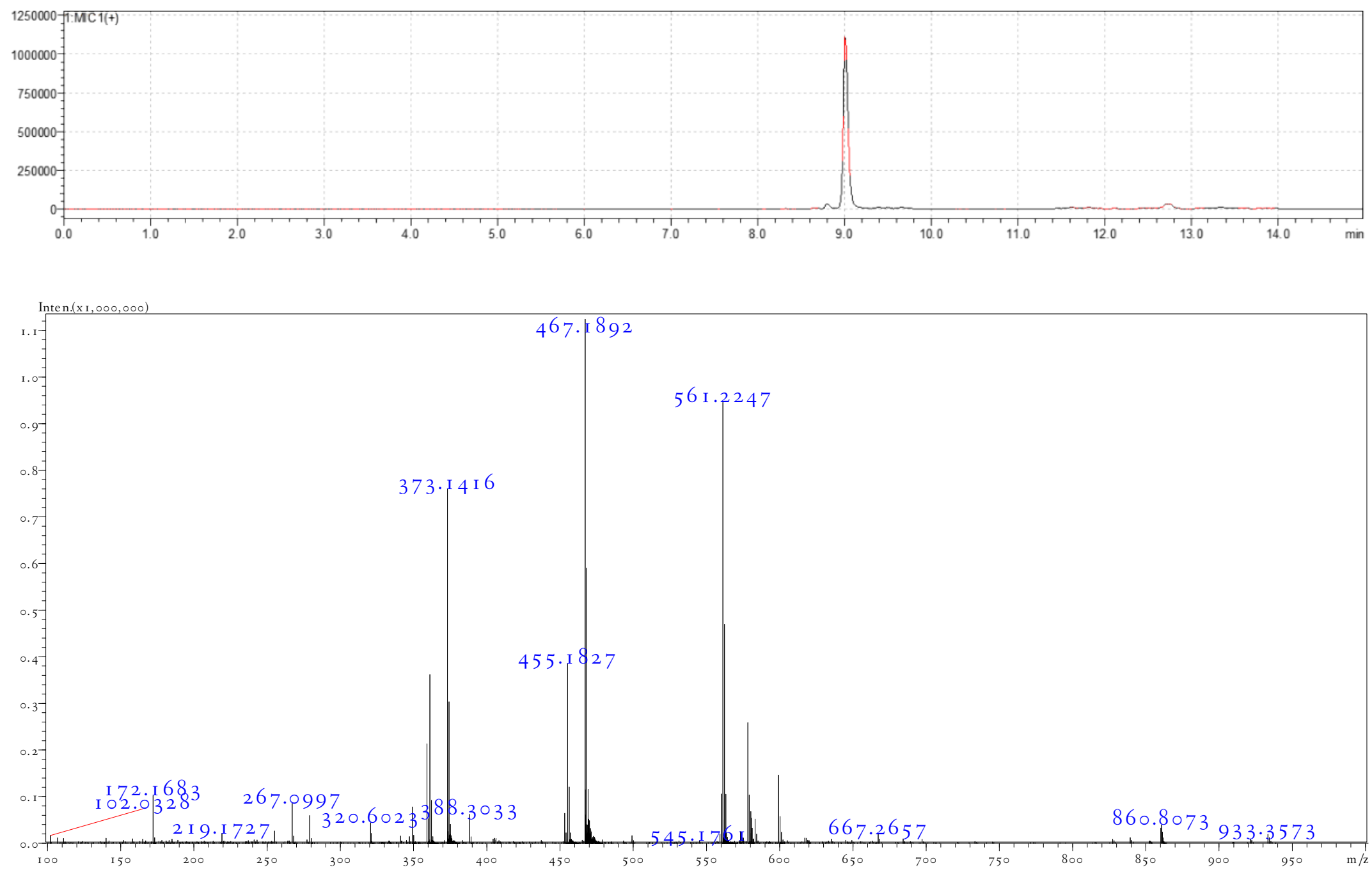

Molecular	[M+H]+, m/z	Cald., m/z	Error (ppm)
$C_{36}H_{32}O_6$	561.2247	561.2272	4.4

Fig. S39. The HR-ESIMS (positive mode) spectrum of compound **6**

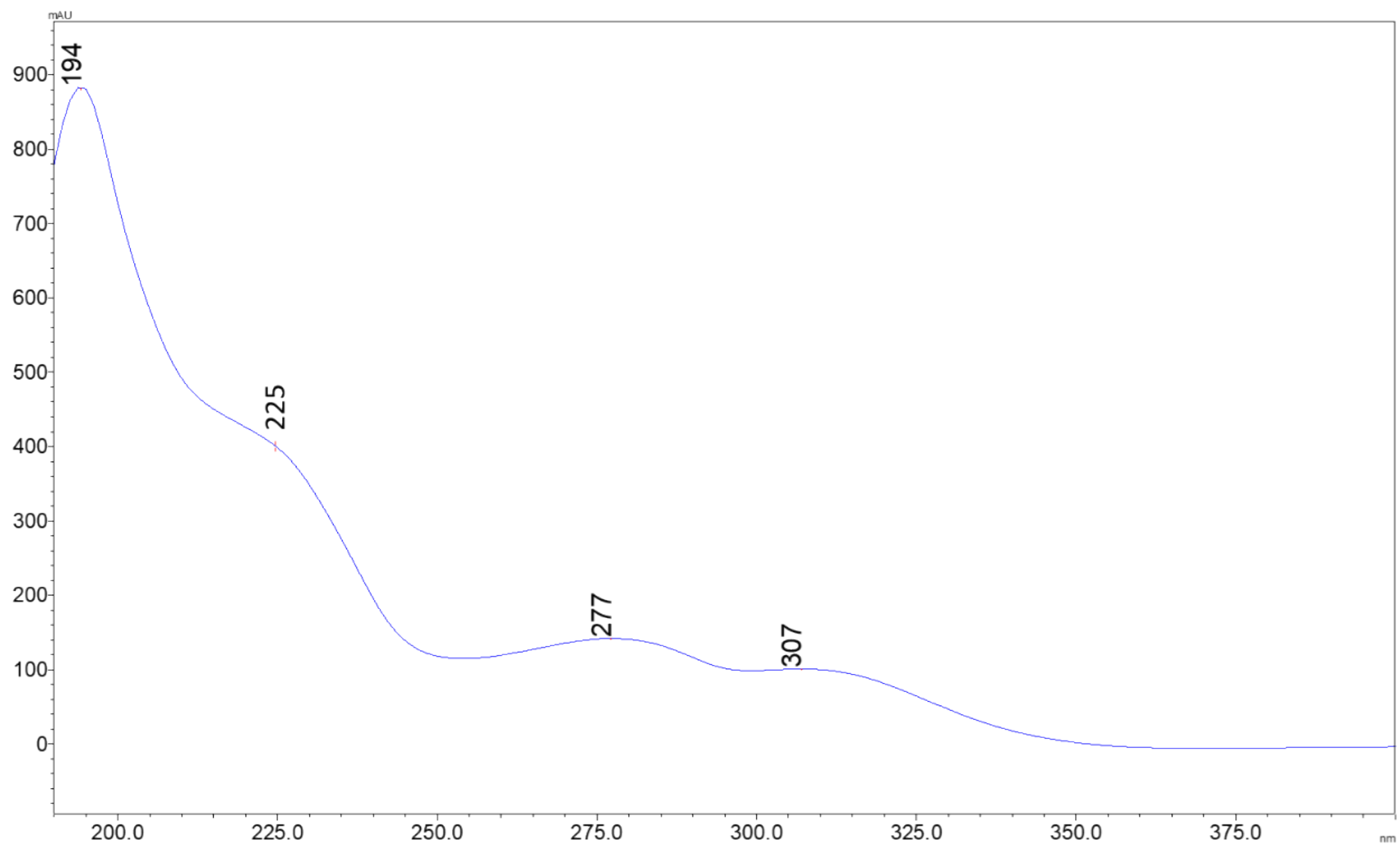

Fig. S40. The UV spectrum of compound **6**

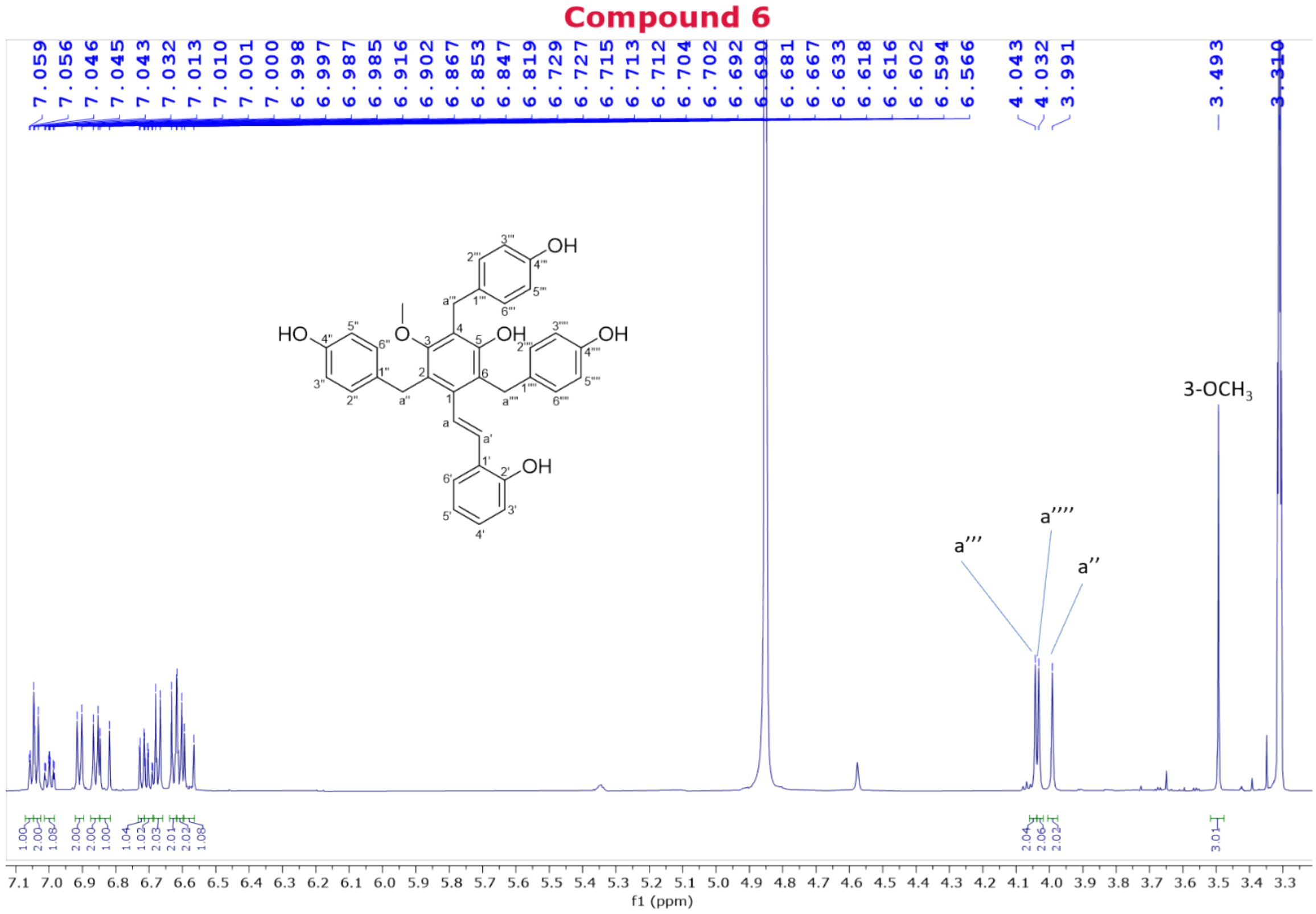

Fig. S41. The ¹H NMR spectrum of compound **6**

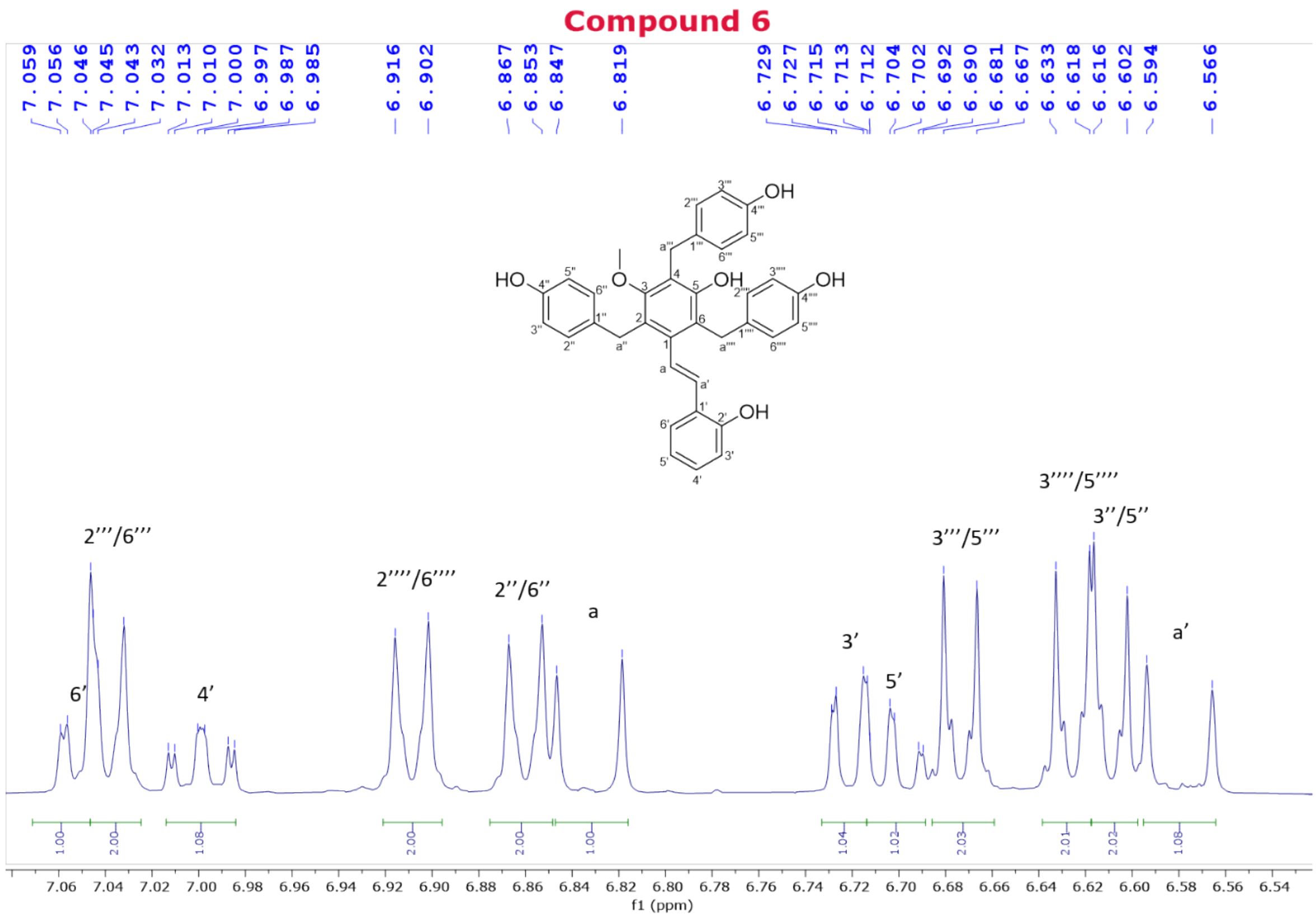

Fig. S42. The ¹H NMR spectrum of compound **6** (range of 6.5 – 7.1 ppm)

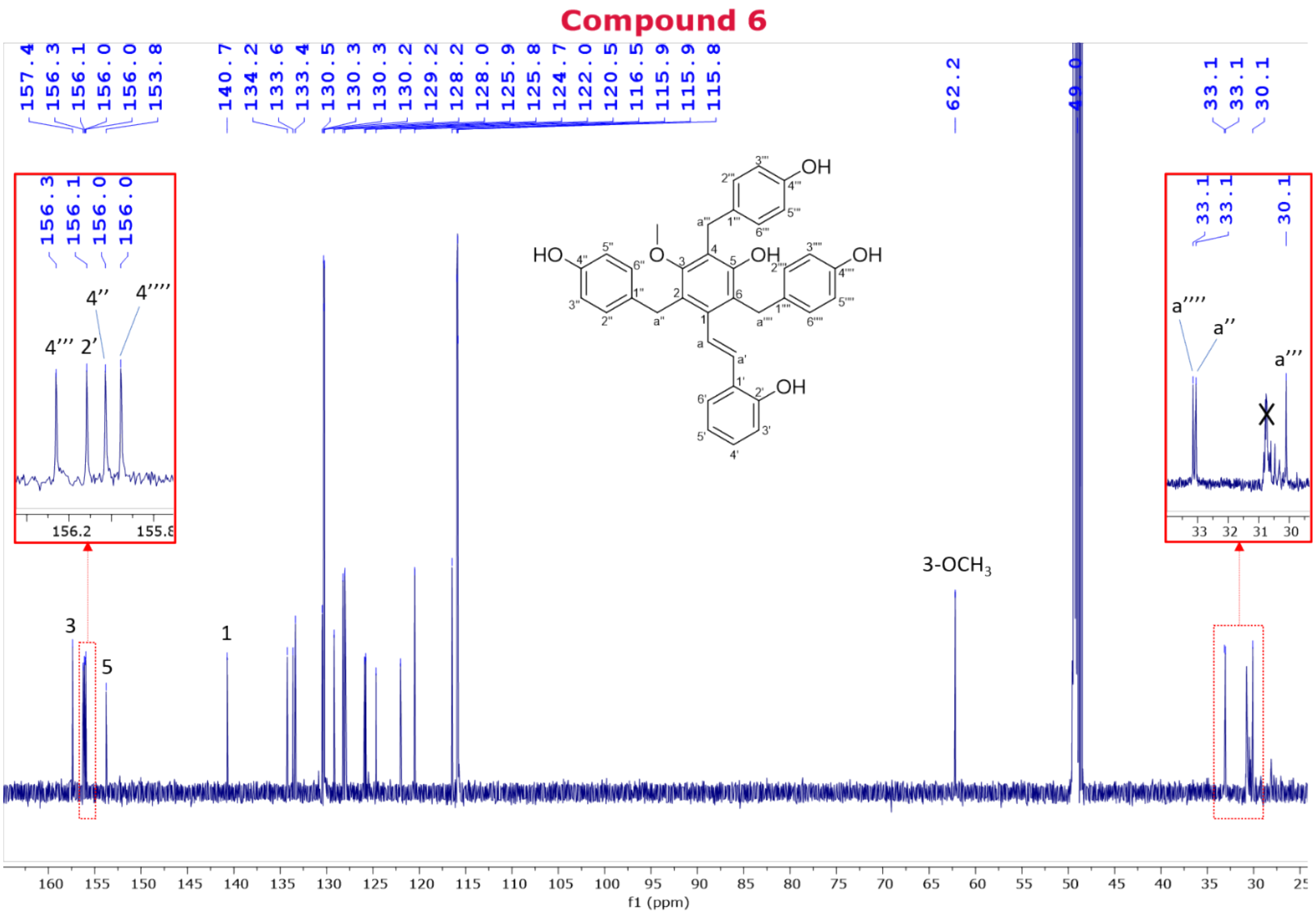

Fig. S43. The ^{13}C NMR spectrum of compound **6**

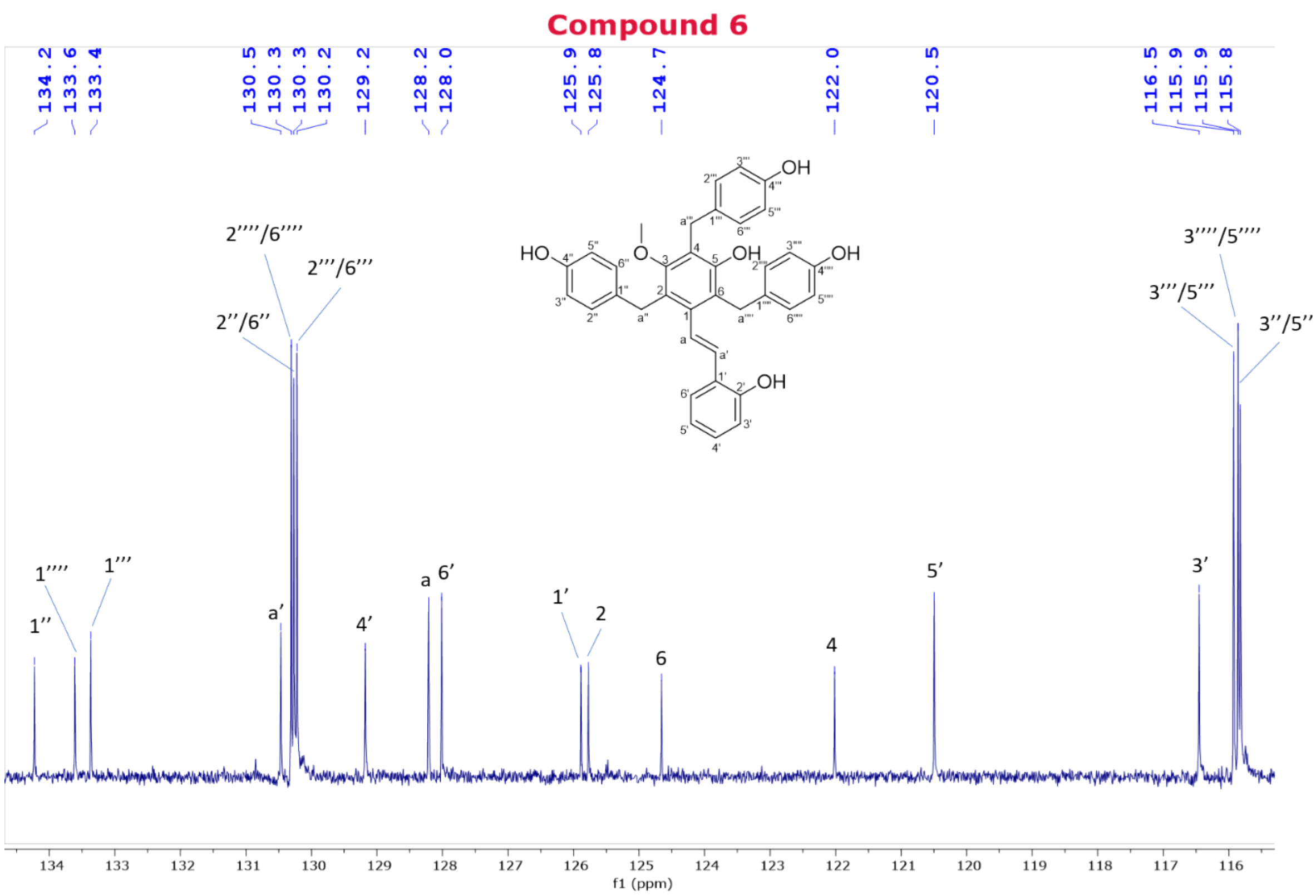

Fig. S44. The ^{13}C NMR spectrum of compound **6** (range of 115 – 135 ppm)

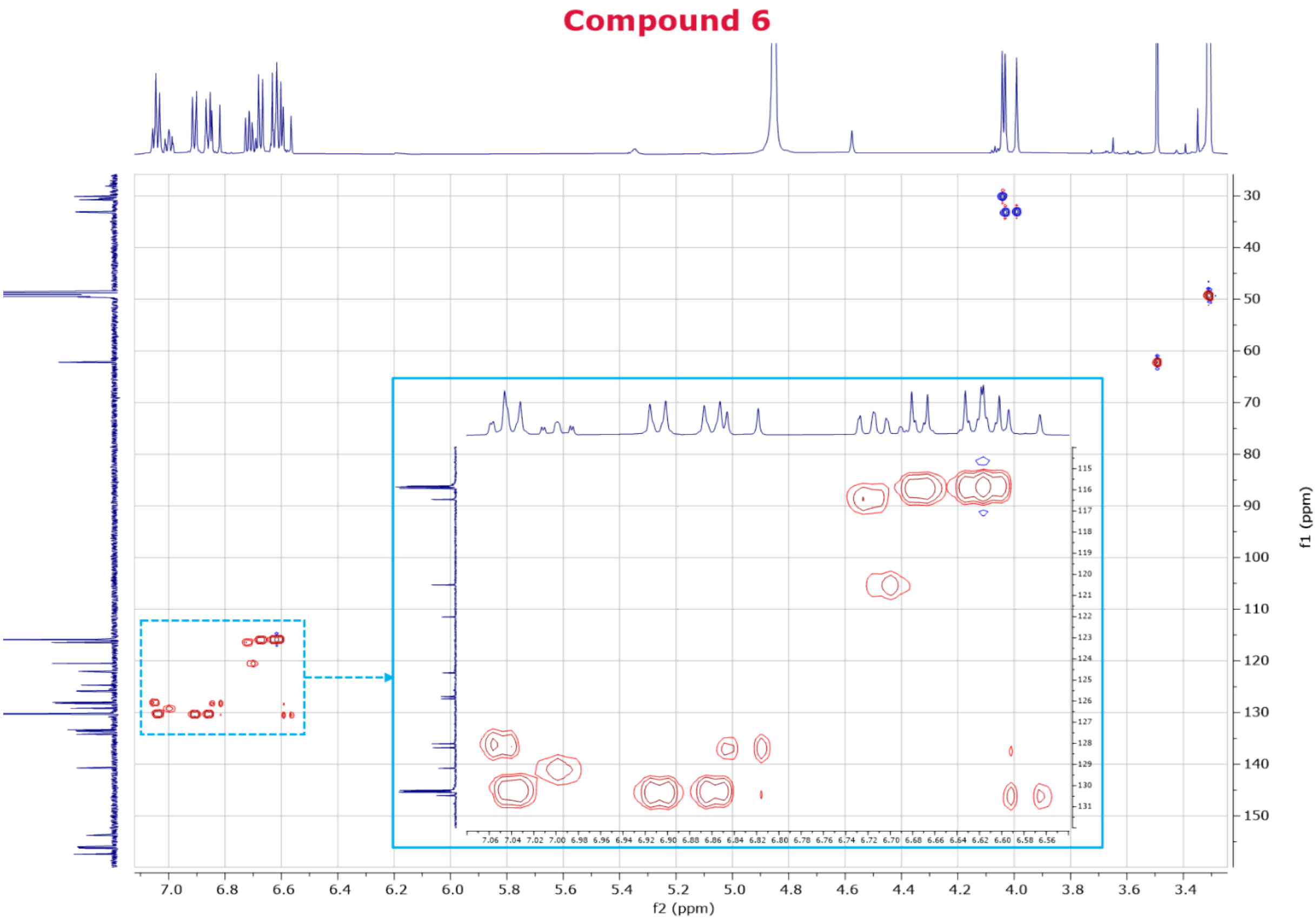

Fig. S45. The HSQC spectrum of compound **6**

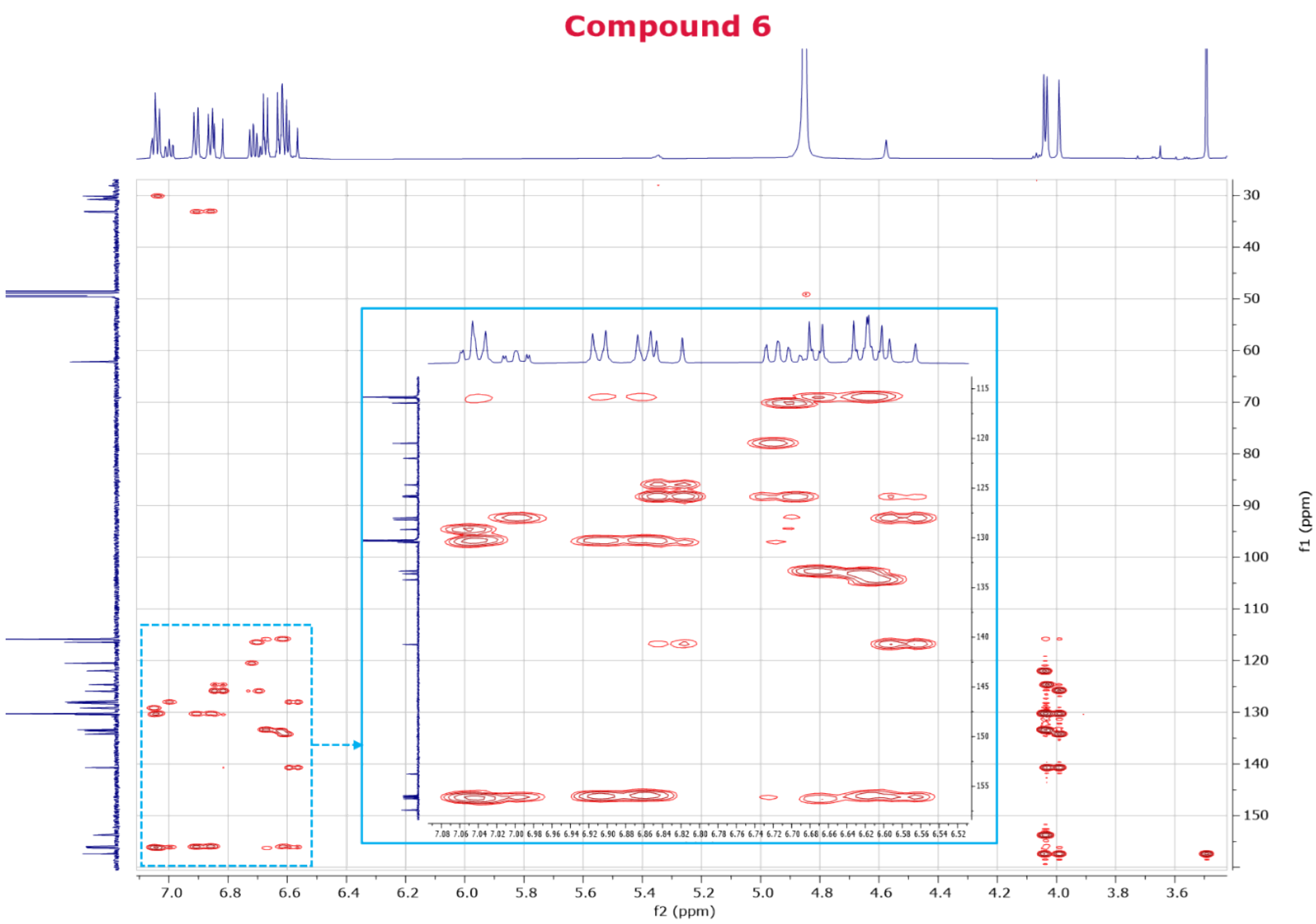

Fig. S46. The HMBC spectrum of compound **6**

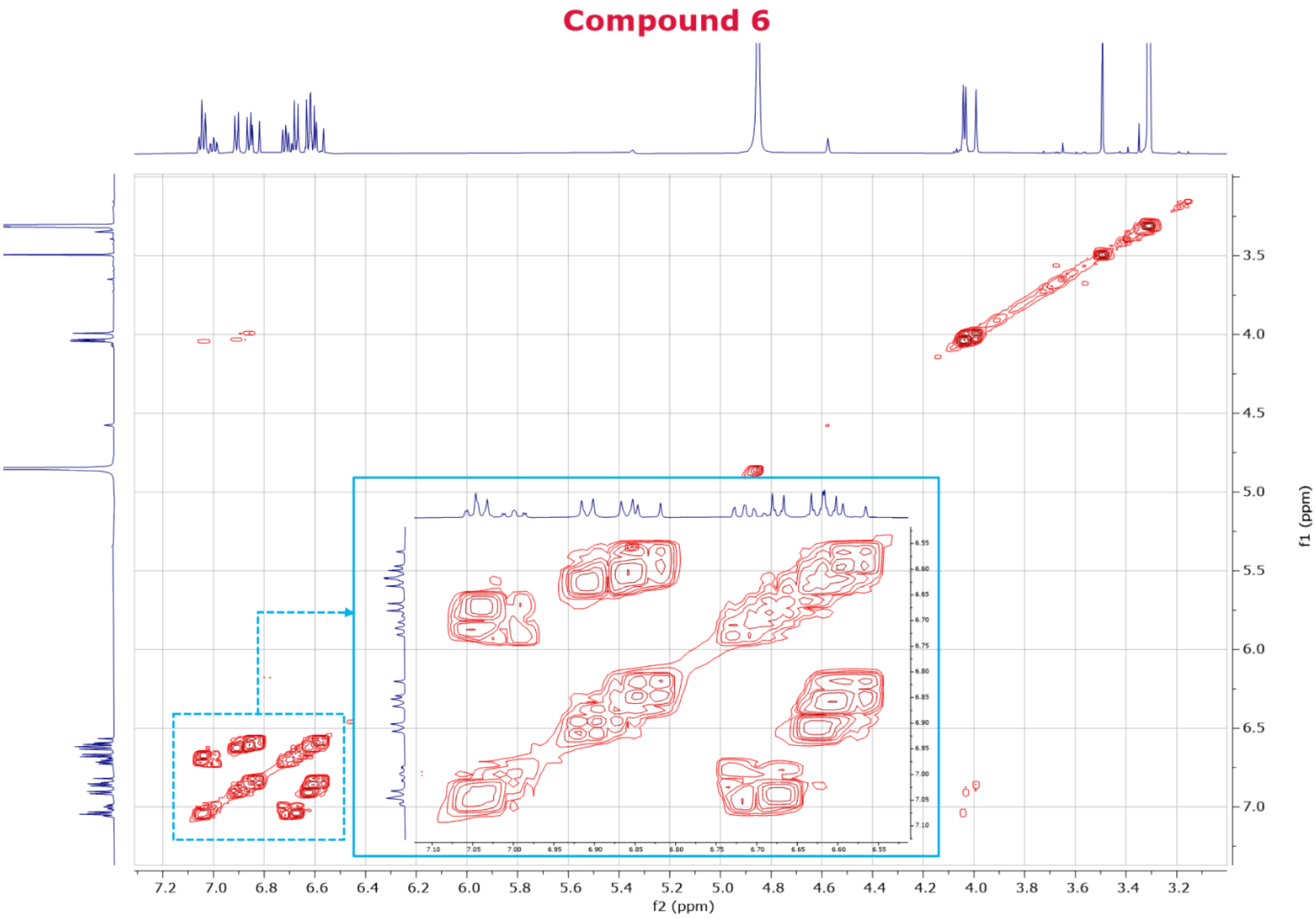

Fig. S47. The COSY spectrum of compound **6**

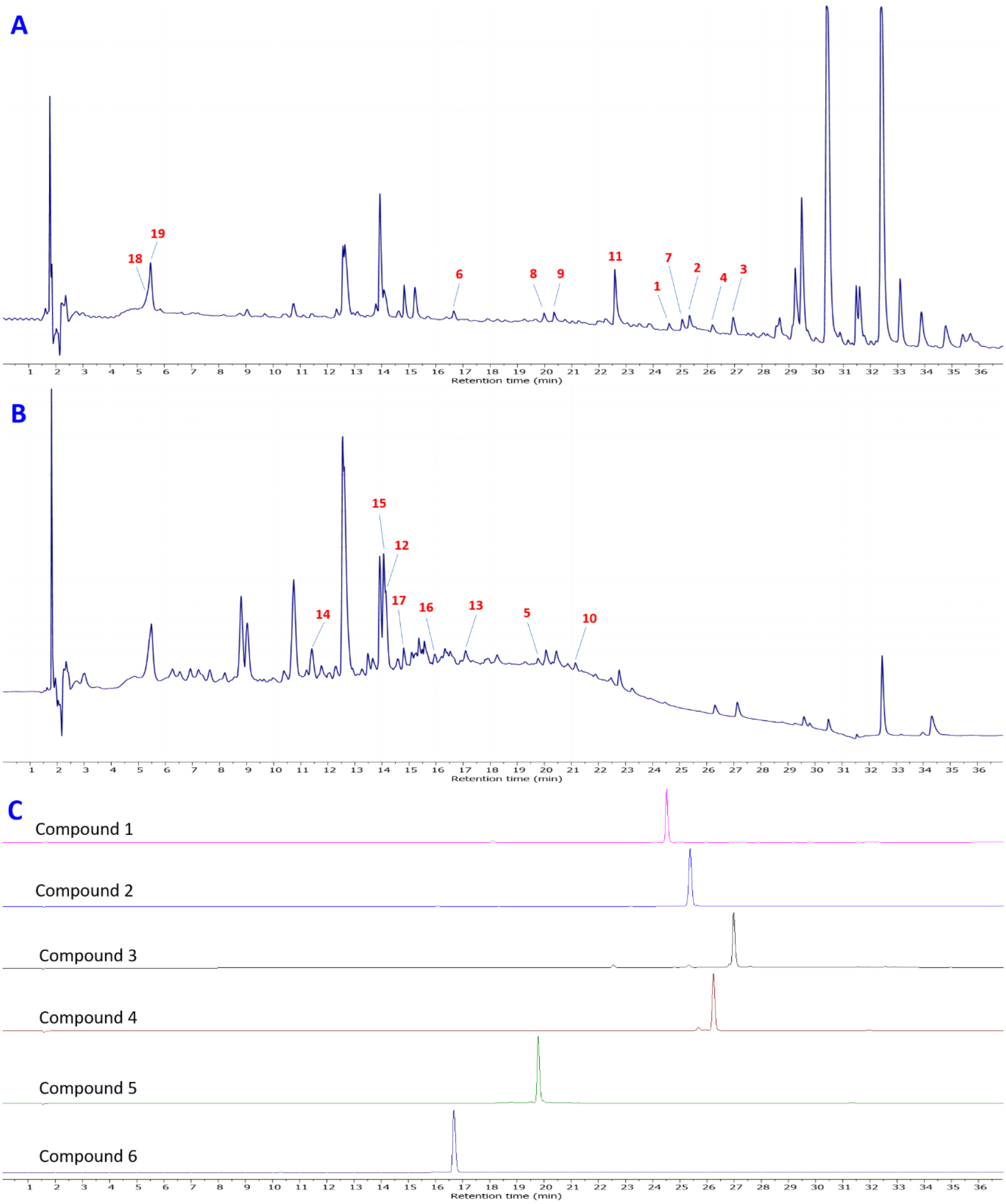

Fig. S48. The HPLC-PDA profiles (200 nm) of the *n*-hexane layer (A), EtOAc layer (B), and six new compounds (C)

Table S1. Molecular docking interactions of compound **6**, **10**, **11**, **12**, 1400W, and BAY 11-7082 with iNOS and IκBα/NF-κB proteins

Ligand	iNOS<br>(PDB ID: 1NSI)	IκBα/NF-κB complex<br>(PDB ID: 1IKN)
**Compound 6**	H-bond: Arg-A199, Gly-A202<br>π-σ: Gly-A202<br>π-sulfur: Met-A355, Met-A434<br>π-π stacking: Phe-A369, Trp-A372<br>π-alkyl: Ala-A197, Cys-A200, Pro-A350<br>π-anion: Glu-A377<br>van der Waals: Trp-A194, Ile-A201, Ile-A204, Gln-A205, Val-A352, Met-A374, Tyr-A373, Arg-381, Trp-A463, Pro-A467, Tyr-A489, Tyr-A491	H-bond: Asp-D226, Leu-D227, Gly-A237, Ser-A238<br>π-cation: Arg-D260<br>π-alkyl: Arg-A236, Arg-A253, Pro-A255, Pro-A256<br>van der Waals: Gly-A209, Gln-D228, Asn-D229, Pro-D230, Phe-A239, Pro-D261
**Compound 10**	H-bond: Arg-A199, Ile-A462, Trp-A463<br>π-cation: Arg-A381<br>π-alkyl: Cys-A200, Ile-A201, Pro-A467<br>van der Waals: Ala-A197, Gly-A202, Phe-A369, Met-A374, Glu-A377, Asp-A382, Arg-A388, Leu-A464, Val-A465, Pro-A466, Trp-B461, Phe-A476	H-bond: Glu-A49, Arg-A50, Arg-D260<br>π-π stacking: Gly-D259<br>π-alkyl: Arg-A50, Arg-A236<br>van der Waals: Ser-A51, Thr-A52, Lys-A221, Glu-A222, Ile-A224, Glu-A225, Gly-A237, Ser-A238, Phe-A239, Ser-A240, Gln-A241, Val-A244, Trp-D258, Pro-D261
**Compound 11**	H-bond: Glu-A377, Ser-A422<br>π-σ: Trp-A194, Ile-A201, Gly-A371<br>π-sulfur: Cys-A200, Met-A434<br>π-anion: Glu-A377<br>π-alkyl: Cys-A200, Met-A374, Ala-A439<br>van der Waals: Arg-A199, Gly-A202, Ile-A204, Gln-A205, Ser-A242, Pro-A350, Val-A352, Phe-A369, Tyr-A373, Trp-A463	H-bond: Asp-A53, Ile-A224, Gly-A237, Arg-A273<br>π-anion: Glu-A225<br>π-π stacking: Phe-A239<br>π-σ: Thr-A52<br>π-alkyl: Lys-A28, Arg-A50, Pro-D261, Pro-A275<br>van der Waals: Ser-A51, Lys-A221, Glu-A222, Asp-A223, Val-A226, Arg-A236, Ser-A238, Gln-A241, Gly-D259, Arg-D260, Ser-A276
**Compound 12**	H-bond: Arg-A199, Gly-A202, Glu-A377<br>π-σ: Ile-A201<br>π-sulfur: Cys-A200<br>π-π stacking: Trp-A194, Trp-A372<br>π-alkyl: Pro-A350, Val-A352, Met-A374, Met-A434, Ala-A439<br>van der Waals: Ile-A204, Gln-A205, Ser-A242, Ala-A351, Phe-A369, Asn-A370, Gly-A371, Trp-A463, Leu-A464	H-bond: Asp-A223, Gly-A237, Gly-D259, Arg-D260<br>π-anion: Glu-A225<br>π-π stacking: Gly-A237<br>π-alkyl: Arg-A50, Arg-A236, Phe-A239, Pro-A275, Pro-D261<br>van der Waals: Lys-A28, Thr-A52, Lys-A221, Glu-A222, Ile-A224, Val-A226, Ser-A238, Gln-A241, Ser-A276,
**1400W**	H-bond: Pro-A350, Phe-A369, Trp-A372, Glu-A377<br>π-σ: Gly-A371<br>π-alkyl: Cys-A200, Val-A352<br>van der Waals: Trp-A194, Gly-A202, Ala-A351, Asn-A370, Tyr-A373, Met-A374	-
**BAY 11-7082**	-	H-bond: His-A245, Arg-A246, Tyr-D251, Asp-D290<br>π-alkyl: Lys-A221, Pro-D281<br>van der Waals: Ala-A242, Val-A244, Gln-D249, Glu-D282, Ser-D283, Glu-D287, Ser-D288, Tyr-D289, Thr-D291,
